# Nonuniform Chiralization of Metal–Organic Frameworks Using Imine Chemistry

**DOI:** 10.1021/acsmaterialsau.4c00139

**Published:** 2025-02-04

**Authors:** Balázs Álmos Novotny, Sauradeep Majumdar, Andres Ortega-Guerrero, Kevin Maik Jablonka, Elias Moubarak, Natalia Gasilova, Nency P. Domingues, Raluca-Ana Kessler, Emad Oveisi, Fatmah Mish Ebrahim, Berend Smit

**Affiliations:** † Laboratory of Molecular Simulation (LSMO), Institut des Sciences et Ingénierie Chimiques, Valais École Polytechnique Fédérale de Lausanne (EPFL), Rue de l’Industrie 17, Sion, Valais CH-1951, Switzerland; ‡ Mass Spectrometry and Elemental Analysis Platform (MSEAP), Institut des Sciences et Ingénierie Chimiques, Valais École Polytechnique Fédérale de Lausanne (EPFL), Rue de l’Industrie 17, Sion, Valais CH-1951, Switzerland; § Interdisciplinary Centre for Electron Microscopy (CIME), École Polytechnique Fédérale de Lausanne (EPFL), Lausanne CH-1015, Switzerland

**Keywords:** postsynthetic chiralization, statistical covalent MOF modification, combinatorial orthogonality, MOF color, MOF sterics

## Abstract

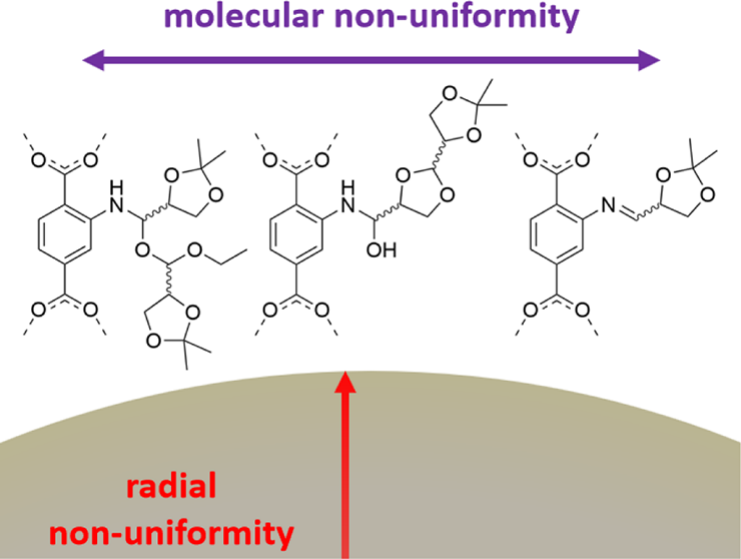

Homochiral metal–organic frameworks (MOFs) are exceptional media for heterogeneous enantiodifferentiation processes. Modifying available achiral structure-bearing MOF scaffolds is a preferred method to extend this class of materials. Reported postsynthetic covalent chiralizations generally lead to uniform, site-specific modifications. The use of chemically versatile modifying agents, like aldehydes, may instead result in the statistical formation of chemically nonuniform anchored products. In addition, the use of such modifying agents gives rise to spatial nonuniformities in the radial direction, due to prohibited diffusion through the MOF bulk. The advantageous grain structure formation plus molecular nonuniformity greatly increase the complexity of such systems. The use of such modifying agents, therefore, necessitates a broader holistic characterization. The present work explores the adaptation of imine chemistry for postsynthetic chiralization. A chiral aldehyde and a chiral ketone are probed on two amine-functionalized MOF substrates—MIL-125 NH_2_ and UiO-66 NH_2_. The UiO-66 NH_2_ modified with the natural product-derived (*R*)-2,2-dimethyl-1,3-dioxolane-4-carboxaldehyde ((*R*)-**1** aldehyde) is found to have the best performance in terms of reactivity and MOF stability. A comprehensive toolbox of methods was demonstrated to robustly characterize the obtained material. This includes high-resolution accurate mass electrospray ionization mass spectrometry (HRAM-ESI-MS) to reveal the competing reactions that yield a set of oligomer-rich structures. *In silico* modeling correctly predicts the localization of the modification. The modification is found to be covalent and chiral and mainly proceeds through imine formation, resulting in a surface enantioselector display formation. Restricted diffusion lengths in the solid phase infer good retention of resolving power in ascending van Deemter régimes in chromatography. Meeting this criterion makes the yielding material a promising potential stationary phase candidate for performant chromatographic enantioseparations.

## Introduction

Homochiral metal–organic frameworks are well-established media for heterogeneous enantiodifferentiation processes.^1−3^ To deliver more such materials, postsynthetic covalent chiralization is generally favored.^4,5^ This avoids the harsh reaction conditions of MOF synthesis, which are feared to result in the solvolysis and racemization of enantioselector moieties. Considering that such modification chemistries are dominated by a couple of highly analogous methods (i.e., predominantly by acylations^4^), diversification of the modes of chiralization merits attention. Enriching postsynthetic methods with covalent linkages new to chiralization would allow for a wider choice of enantioselectors with more relaxed chemical constraints.

A survey of covalent-organic postsynthetic modification strategies outside the scope of chiralization reveals two pertinent ligations: phosgene^6^ and imine^7^ chemistries. More marginal examples are cycloadditions beyond click chemistry^8^ and the use of an epoxidized MOF as an alkylating matrix.^9^ Phosgene and thiophosgene may produce potent isocyanate and isothiocyanate acylating groups from amino groups on either the substrate^10−12^ or the modifying agent.^13−17^ This versatile system may yield activated crystalline substrates that readily covalently trap nucleophilic agents in the pores. Highly pronounced safety concerns pertaining to gaseous carcinogens and the necessity of strictly anhydrous and otherwise nucleophile-free systems, however, deter further exploration.

Like phosgene chemistry, postsynthetic covalent imine appending has also been used with the reactive functionalities on either the substrate or modifying agent. Aldehyde-bearing substrates, both a dicarboxylate ligand^18^ and ZIF-90,^19^ have been submitted to this reaction. Amine-bearing substrates have been reportedly modified with salicylic aldehyde,^20−25^ aldehyde-bearing pyridine derivatives,^26,27^ aldehyde-bearing imidazole,^28,29^ 8-hydroxy-2-quinolinecarboxaldehyde,^30^ aldehyde-bearing pyrene,^31^ 2,3,4-trihydroxybenzaldehyde,^32^ and formaldehyde.^33^ Many such modifications have culminated in postsynthetic metalation, introducing new coordination sites.^20−23,25−28,30^ Imine functionalization with a ketone was reported in the context of a direct reaction with an acetylacetone metal complex, yielding covalent modification and postsynthetic metalation in a single step.^34^ Interestingly, achiral modification via imine formation has also been performed on aminoacyl chiralized MOFs.^35,36^

Imine formation involves linking amino groups with oxo compounds in warm ethanolous media. Considering the convenience of this reaction, which has not been reported for MOF chiralization to date, to the best of our knowledge, its application for chiralization should be pursued.

In this work, we adapt imine chemistry for postsynthetic MOF chiralization as follows. Considering the poor stability of aldehydes and the modest accessibility of aldehyde-bearing MOF substrates, the MOF is assigned to bear the amino precursor of the Schiff-base linkage. Given the ample literature on and broad precursor availability for aminoterephthalate MOFs in general,^37,38^ and aminoterephthalate analogs of MIL-125^39^ and UiO-66 in particular,^40^ these two structures were chosen. For the oxo compounds, simple archetypal chiral natural products were targeted. Glyceraldehyde, the simplest aldose, was chosen in the form of its acetonide-protected derivative. This may be conveniently derived via the periodate cleavage of mannitol diacetonide.^41,42^ Less reactive oxo compounds, ketones, are used to assess the reactivity limitations. For the ketone, a rigid, configurationally locked, sterically confined camphor was chosen. The resulting two-by-two combinatorial space was probed to assess the stated reactivity limitations, substrate stability, steric confinement, and reagent penetration. Acetaldehyde and acetone were added as positive controls for reactivity, as they are more permissive with regard to steric exclusion and diffusion rates, considering their size. Ease of handling was also considered.

The primary question is whether and when compositional change can be achieved while the backbone of the MOF substrate is structurally and solvolytically intact. This is termed reactive orthogonality. Second, we confirm that the modification is indeed a covalent chiralization via imine formation. Finally, we investigate what sorption-based separation such modification allows based on the resulting grain and pore structures.

We use a breadth of characterization techniques, thoroughly studying the modification from bulk composition to the molecular level. These include recent advances in MOF color analysis to track imine formation,^43^*in silico* methods to aid material evaluation within chiral functionalization, and pore analysis to study the distribution of the modification. The versatility of aldehyde chemistry results in chemically nonuniform modifications that challenge characterization efforts. Postsynthetic covalent surface modifications of MOFs have previously been reported with highly elaborate architectures.^44,45^ Regardless of the resulting nonuniform radial distribution, reported postsynthetic covalent chemistries characteristically lead to chemically uniform, site-specific modifications. Showcasing the use of HRAM-ESI-MS to discern statistical product formation in the context of postsynthetic covalent MOF chiralization therefore became the highlight of our contribution.

**Figure 1:**
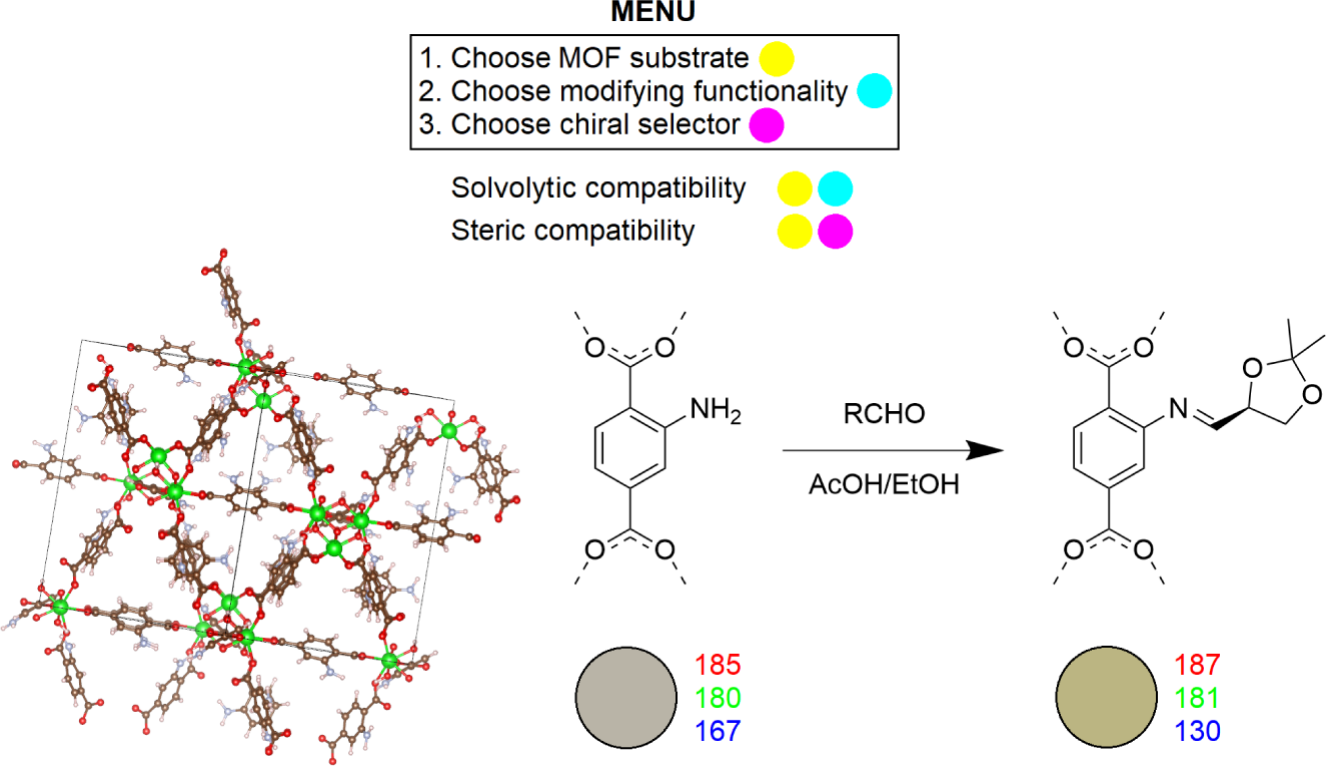
A color-coded scheme illustrates the compatibility criteria probed to find the flagship MOF substrate and chiral modifier combination. The structure of the UiO-66 NH_2_ MOF substrate and the formal imine modification yielding from the (*R*)-**1** aldehyde treatment are, thereafter, shown. This formal modification was revealed to be a representative but nonexclusive product of the treatment.

Our results demonstrate the applicability of covalent imine linkage^7^ to chiralization. Screening of the MOF substrate and modifier pairs reveals which combinations of the necessary reaction conditions the MOF substrate can withstand without degradation. The number of viable experimental combinations is, therefore, limited by the previously defined reactive orthogonality criterion. (*R*)-**1** aldehyde-modified UiO-66 NH_2_ was identified as the flagship combination and studied further. In Figure 1, the flagship combination, along with the formal imine product of the modification, is shown.

Pore analysis reveals that the modifier may not access the MOF pores, even as a monomer, suggesting a dense enantioselector display on the grain surface. This implies short diffusion lengths to reach the chiral selectors, which is favorable in chromatographic separation processes.^5,46^

## Methods

### Preparative Methods

Starting MOF substrates MIL-125 NH_2_^47^ and UiO-66 NH_2_^48^ were previously synthesized according to the referenced procedures. Further materials were obtained and used as described in Section S1.1. Modifications were carried out in warm ethanolous media using acid catalysts as described in Section S1.2. Homogeneous model experiments were carried out to assess reactivity without the kinetic limitations of heterogeneous systems. These experiments were performed in deuterated media, under deoligomerization conditions and conditions replicating solid-phase treatment. Homogeneous model experiments are described in detail in Section S1.3. Solid isolates were digested for analysis in nucleophilic, ammonia-free, aqueous solution, or a pH-neutral aqueous media. Digestion methods are described in detail in Section S1.4.

### Characterization Techniques

Thermogravimetric analysis (TGA) traces were obtained on a NETZSCH TGA209 F1 Libra instrument. Elemental analyses were performed on an Elementar UNICUBE instrument with samples measured in triplicate. Powder X-ray diffractograms (PXRD) were accumulated on Bruker D8 Advance diffractometers. Scanning electron microscopy (SEM) images were acquired on a Thermo Fisher Scientific Teneo system. Photography was performed in an illuminated ML-4030 LED Maxi Light Box with a Datacolor Spyder CHECKR 24 color correction card, using the main camera of a Tecno Camon 17 cell phone. White point color correction was done according to the reported procedure.^43^ Ultraviolet–visible (UV–Vis) diffuse reflectance spectra were obtained by using a PerkinElmer Lambda 850 UVUV/Vis Spectrometer. Fourier-transform infrared spectroscopy (FT-IR) was performed on Spectrum Two from PerkinElmer, with background subtractions, using direct measurement from treated MOF powders. All nuclear magnetic resonance (NMR) experiments were performed with a Bruker (AV-III) spectrometer equipped with a 5 mm BBO probe head capable of producing magnetic field pulse gradients in the *z*-direction of 54 G·cm^–1^. High-resolution accurate mass electrospray ionization mass spectra (HRAM-ESI-MS) were accumulated using an automated chip-based nanoelectrospray device (Triversa Nanomate, Advion, Ithaca, USA) coupled to an Orbitrap Exploris 240 FT-MS instrument. Circular dichroism (CD) spectra were obtained on a Chirascan V100 spectropolarimeter, with samples being held in a quartz cuvette of 1 mm path length. Necessary dilutions were performed with the respective neat solvents. Further details on all experimental techniques are described in Section S1.5.

## Results and Discussion

Results and discussion are organized according to three main approaches. Analyses of the bulk composition reveal quantitative aspects of the modification attempts and key reactivity trends. The molecular-level inquiry lines up evidence from organic chemistry characterization techniques. Computational structural modeling reveals the sterics of the system to help find a comprehensive interpretation of these two groups of experimental results.

### Compositional Change in Light of Solvothermal Stability of Substrate

Here, we use a combination of experimental techniques to show how the bulk composition of our samples evolves upon an attempted modification. In particular, we used TGA to probe the thermolabile mass fractions. Elemental analysis is used as a complementary technique to ascertain the nature of mass incorporations. Solution color and yield are further considered as qualitative indicators for the solvolytic substrate dissolution. PXRD is used to probe the crystallinity of solid isolates. SEM is used to confirm that the overall morphology of the particles and specifically their crystal habit do not change and to confirm their solvolytic degradation.

#### TGA: Bulk Modification

The measurement method for thermogravimetric traces, described in Section S1.5, allows for the quantitative removal of organic components. Assuming complete sample calcination, the composition is referenced to stoichiometry by normalizing thermogravimetric traces to the residual mass. This results in a decay curve that reveals the relative thermolabile mass. Subtracting the normalized traces of the modifying agent-free control from that of an attempted modification reveals the decay profile of thermolabile relative mass increase that is creditable to the modifier. Assuming full conversion, the expected thermolabile mass ratios for all traces are found, and realized conversion is inferred. The expected thermolabile mass ratios are shown in Tables S1 and S2.

**Figure 2:**
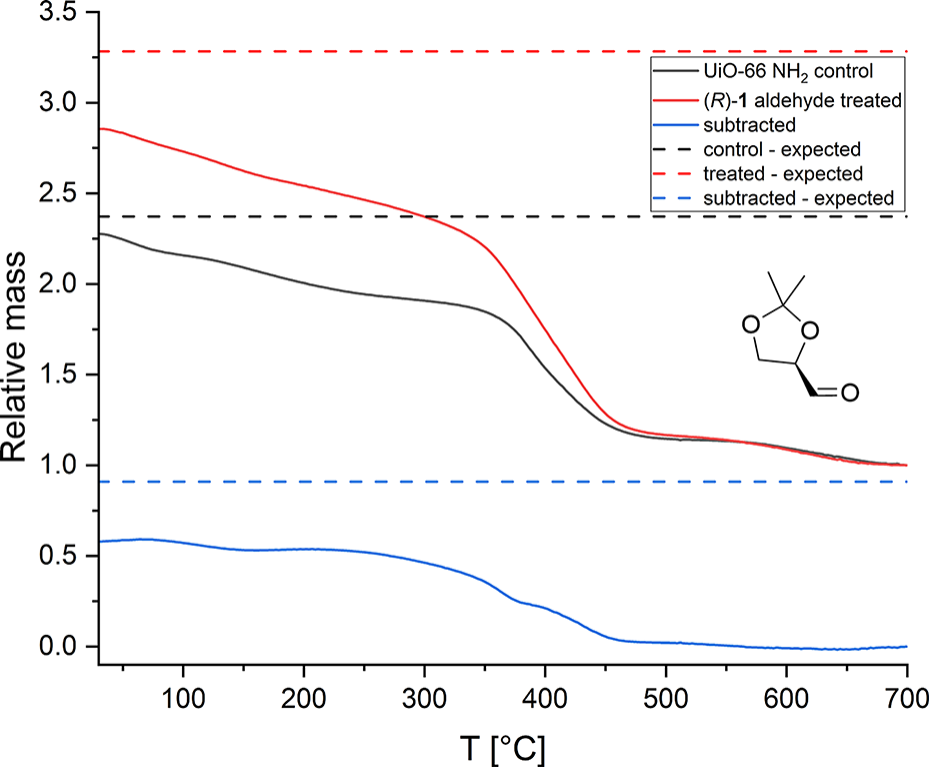
Thermogravimetric traces normalized on the calcined residues. Respective traces for the treated substrate, control, and their difference are shown. Corresponding expected thermolabile relative masses, based on the stoichiometry of full conversion and complete desorption of potential guest molecules, are projected across the temperature series. The plot pertains to the UiO-66 NH_2_ substrate treated with oxo compound (*R*)-**1** aldehyde.

Both aminoterephthalate MOFs, MIL-125 NH_2_ and UiO-66 NH_2_, were analyzed with four oxo compounds: acetaldehyde, acetone, the chiral aldehyde, and D-camphor. As negative controls, (i) the untreated MOFs and (ii) modifier-free controls, i.e., MOFs treated under matching conditions in the absence of oxo compounds, are also analyzed. The analysis is detailed in Section S2.1. All thermogravimetric traces of concern, plotted in Figures 2 and S1–S7, reach a thermostable plateau, confirming quantitative calcination.

The MIL-125 NH_2_ modifier-free control, plotted in Figures S1, S3, S4, and S6, falls short of expectations on thermolabile relative mass, prompting concerns about solvolysis. The moderation of such a decrease for the analogous UiO-66 NH_2_ probes, plotted in Figures 2, S2, S5, and S7, makes a strong case for its superior stability.

Probes with acetaldehyde, plotted in Figures S1 (MIL-125 NH_2_) and S2 (UiO-66 NH_2_), exhibit a relative organic mass incorporation increase that exceeds expectations in both MOFs. This may be credited to a change in interface polarity improving interactions with solvent molecules. The temperature profile, showing a considerable slope in low-temperature régimes, is consistent with the desorption of physisorbed solvents. Relative increases for probes with the chiral (*R*)-**1** aldehyde, on the other hand, plotted in Figures S3 and 2, fit within expectations. The low-temperature sloping of their profiles, however, draws caution for a deeper interpretation. Probes with acetone, plotted in Figures S4 and S5, exhibit profiles reflecting thermal stability despite expectations of a poorer extent of covalent attachment. Disconcerted thermal events complicate the interpretation of traces for the d-camphor-treated UiO-66 NH_2_, plotted in Figure S7. Notably, the harsh conditions for reacting d-camphor significantly reduce the thermolabile relative mass for MIL-125 NH_2_ (see Figure S6), suggesting solvolytic ligand leaching.

The following observations supported thermogravimetric findings. Solution color inferred partial MOF dissolution. Trends in solvolytic substrate stability were confirmed by the presence of the imine chromophore in reaction mixtures. The UiO-66 NH_2_ substrate was determined to perform considerably better with regard to ligand retention under relevant reaction conditions, as already proven by the quantitative evidence provided by TGA. Observations of solution color are described in Section S2.3. The mass of the isolates also inferred partial MOF dissolution. Given how desired modifications are incorporations, the mass of the isolated solid may unequivocally reveal substrate solubilization if it is inferior to the starting amount. Observations have strongly confirmed the already established trends in solvolytic substrate stability, as discussed in Section S2.4.

These findings stress the limitations in the compatibility of the surveyed chemical space. The present study, consequently, prioritizes the substrate with better solvolytic stability and the oxo modifier with better reactivity. The MIL-125 NH_2_ D-camphor system is thus excluded from further inquiry. The achiral positive controls were not used for further analysis with the exception of the FT-IR study.

#### Elemental Analysis: Bulk Modification

Elemental analysis adds further detail concerning the composition of the surveyed solids. Given the modest number of channels in CHN analysis and the diversity of species the isolates have encountered from the MOF syntheses, results are limited to generalized interpretation. The N channel boasts specificity, as it may indicate only N,N-dimethylformamide (DMF) retained from the syntheses, apart from the aminoterephthalate ligand. As such, if the presence of DMF is not revealed in controls, the N content of the ligand serves as an internal standard to reference the CH content. From the CH channels, the degrees of unsaturation for incorporated species may be inferred. This serves as a basis for holistic analysis: higher H content is associated with solvent-rich isolates, and higher C content is associated with modifier-rich isolates.

Results are shown in Section S2.2, separately, for substrates MIL-125 NH_2_ and UiO-66 NH_2_. A comparison of expected results for the negative controls and hypothetical bulk modifications with experimental data is presented in Tables S3–S8, pairwise for the respective substrates. Comparisons are plotted in Figures S8 and S9 for the respective substrates. The MIL-125 NH_2_ system was found to retain DMF from the solvothermal synthesis, based on its N content, while for UiO-66 NH_2_, the results were in line with the theoretical ligand N contribution. The three surveyed chiralization attempts all resulted in a carbon-dominant increase in the organic content. Such an increase consistently fell short of the theoretical values for uniform modifications. It is worth noting, however, that unlike TGA, insight from elemental analysis is provided only for the room temperature isolates, leaving no room to exclude contribution from physisorbed species. In summary, findings support the success of modifier incorporation into recovered solids and demonstrate that quantitative appending of structural ligands may not have been obtained.

Given that the calcined residues are known to be titania and zirconia, TGA also serves as an elemental analysis technique for Ti and Zr content, respectively.

#### PXRD: Retention of Backbone Crystallinity

**Figure 3:**
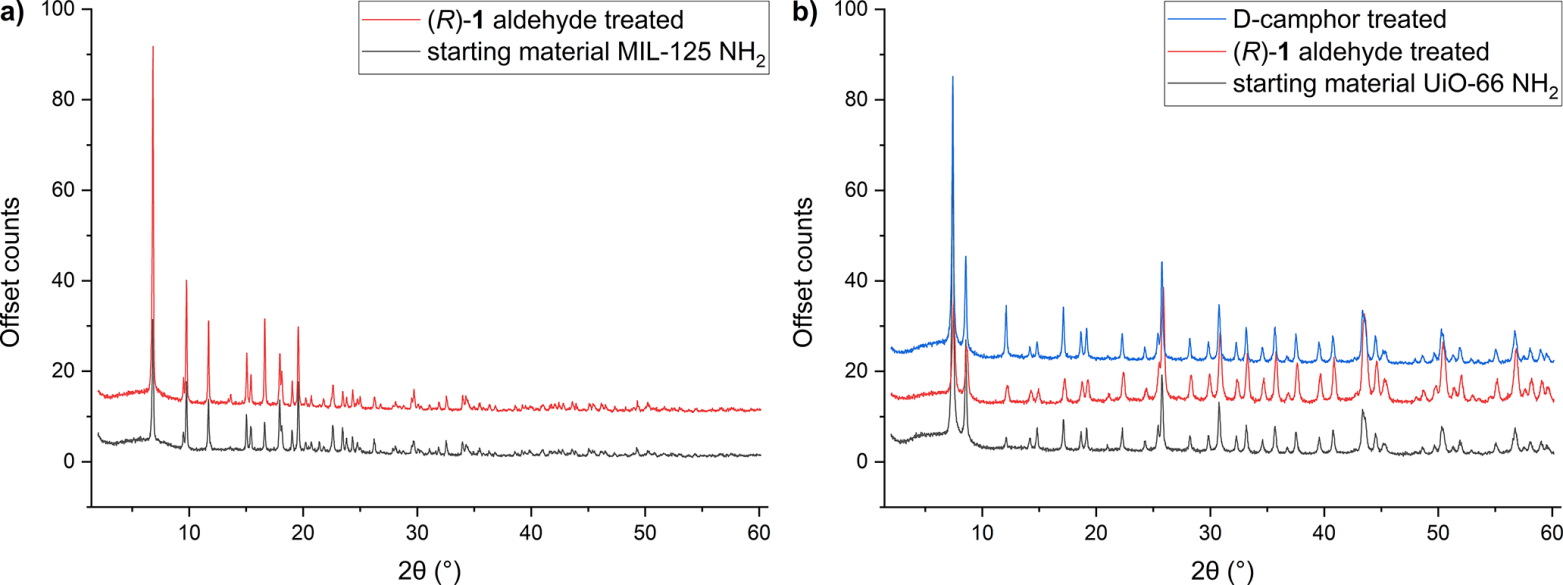
Pattern retention of the powder X-ray diffractograms for MIL-125 NH_2_ (a) and UiO-66 NH_2_ (b) substrates. The plots stack diffractograms of the untreated and treated substrates.

The crystallinity of the solid isolates was probed by PXRD, with diffraction patterns closely matching those of the respective starting substrates, as demonstrated in Figure 3. Reflections in PXRD patterns correspond to distances inherent to respective sets of parallel planes. The size of the electron shell of the concerned atoms determines the intensity of such reflections. Consequently, in the present study, PXRD primarily reveals the relative position of metal nodes. This outcome reveals that the structural backbone of the framework, spacing, and relative angular position of the nodes have been largely left intact in the recovered solids. Whatever modification may have thereby been performed can be, therefore, considered orthogonal to the backbone structure. This infers that the modification is either restricted to the surface or does not sterically induce distortion in the bulk.

#### SEM: Retention of Morphology

Scanning electron microscopy (SEM) images reveal the studied MOF particles’ overall morphology and surface features. In the context of postsynthetic MOF modification, it is an important tool for assessing changes upon treatment. It can reveal whether the crystal morphology has changed. It can also reveal signs of solvolytic degradation upon treatment. For this study, we imaged both MIL-125 NH_2_ and UiO-66 NH_2_ starting substrates and respective isolates from negative control treatments. Furthermore, we imaged the UiO-66 NH_2_ substrate treated with oxo compound (*R*)-**1** aldehyde. The overall morphology of the particles and, specifically, their crystal habit does not change for either of the three treated isolates, in concert with the found retention of PXRD patterns. Signs of solvolytic degradation are clear on the crystals’ surface for all three treated isolates, in concert with TGA and elemental analysis results. For the MIL-125 NH_2_ substrate, solvolytic degradation is exhibited by forming a rougher surface on the rounded platelets. The UiO-66 NH_2_ substrate is exhibited by rounding of the edges of the intergrown crystals with hexagonal faces. Interestingly, the (*R*)-**1** aldehyde-treated UiO-66 NH_2_ isolate exhibited less solvolytic degradation than the corresponding isolate from the negative control experiment. This shows that surface functionalization can moderate the solvolytic degradation. Detailed analysis is described in Section S2.5.

### Covalent and Chiral Nature of Modification

Here, we use a combination of experimental techniques to study how the molecular-level composition of the samples evolves upon the attempted modification. We assess the color change and use FT-IR spectroscopy to probe the formation of the imine chromophore. We use NMR to study components of the solution state model systems. Importantly, HRAM-ESI-MS is applied to elucidate the composition and formation route of solubilized product mixtures. Enantiomeric excess is probed by using CD spectroscopy.

#### Color Change: Covalent Modification in Shell

The characteristic color of the imine chromophore may be exploited as an immediate indicator, not only in solution but also on treated MOFs. This is seen in photographs of the starting materials and isolates from attempted chiralizations, taken where the yield was sufficient for the latter. The color-calibrated photos are shown in Figures S17–S21.

A comparison of calibrated colors reveals a shift that is statistically significant in all of the photographs that were studied. A pronounced color shift is visually observed in all cases for the attempted modifications involving aldehydes. For MIL-125 NH_2_, it changes from ripe yellow to a dark orangish-brown color, while for UiO-66 NH_2_, it changes from light cream to a grayish dark brown color. However, no visually unequivocally interpretable color shift was seen with acetone. As with the d-camphor, under the harsher conditions, the color shift for the MIL-125 NH_2_ was pronounced, while for UiO-66 NH_2_, it was less apparent.

**Figure 4:**
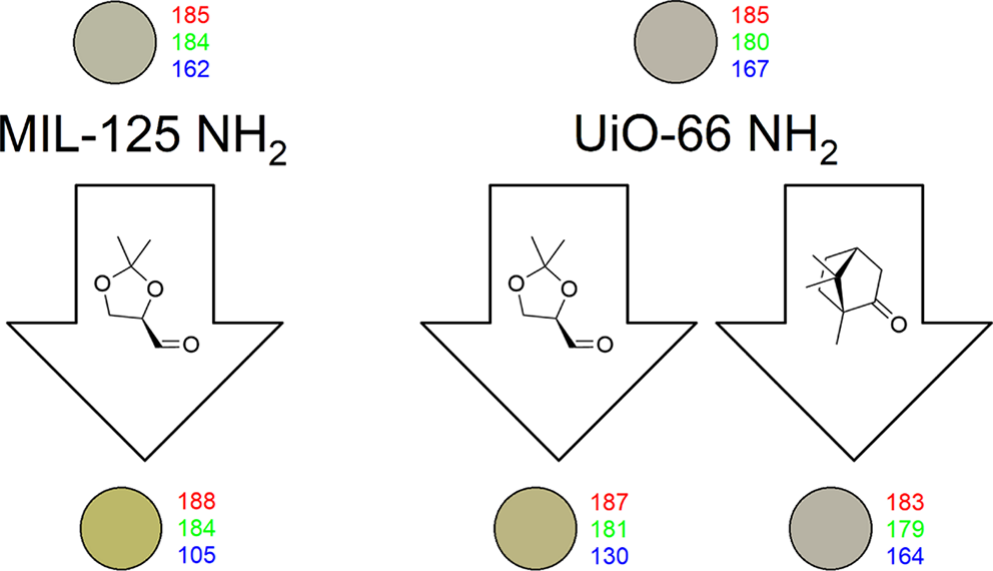
White point corrected colors of untreated and treated substrates are compared with the probe for the orangish color shift attributable to the imine chromophore. A representative average of each color is sampled and described with the corresponding RGB code. Substrates (top) and chiral modifiers (arrows) are indicated.

The outcome gives strong direct evidence that all aldehyde treatments result in the formation of imine in the solid phase. Bleak evidence of the same in the case of ketone treatments further discourages pursuing half of the chemical space. While it is a direct indicator of the covalent modification on the solid, it is important to note that isolate color does not reveal the radial distribution of the modification. A detailed analysis is described in Section S3.1 and summarized in Table S9. In Figure 4, the calibrated colors are shown, as disclosed in Table S10.

The light absorption of MIL-125 NH_2_ and UiO-66 NH_2_ starting materials and their (*R*)-**1** aldehyde-treated isolated samples is further evaluated by collecting diffuse reflectance UV–Vis spectra. From their absorption spectra, the CIE 1931 color coordinates (x, y) are determined and mapped onto a chromaticity diagram.^49^ The obtained colors are consistent with the photographic analysis described in Section S3.1. For numerical comparison with the photographic analysis, the RGB color codes are also converted to (x, y) CIE 1931 color coordinates.^50−52^ The yielding color coordinates calculated from the visible absorption spectra in diffuse reflectance and the photographic analysis are also in good agreement, validating the photographic method. Detailed analysis is described in Section S3.2.

#### FT-IR: Covalent Modification in Shell

The FT-IR spectra infer the underlying functional group chemistry, much like the appearance of a visually observable chromophore. Spectral evidence of covalent modification was found where expected. The spectra are described in Section S3.3 and shown in Figures S24 and S25. While this spectroscopic method is considerably superior to photography in spectral resolution and breadth, it shares similar limitations regarding grain shell penetration. These findings, therefore, only back color-based evidence of modification on the surface. Conclusive primary evidence of the chemical nature of the products is provided by HRAM-ESI-MS.

#### NMR: Mixture Formation

NMR is a powerful tool to ascertain the molecular structure of a species in solution; however, its applicability in the present work is indirect. Model 1 experiment was constructed to elucidate the characteristic and distinct chemical shifts of the target product. The experiment was tracked by one-dimensional NMR techniques, and the yielding matter was studied by two-dimensional NMR techniques. Spectra are described in Section S3.4. One-dimensional spectra are shown in Figures S26–S30, S32–S34, and S36, and two-dimensional spectra are shown in Figures S35 and S37–S39. Molecular motifs of reagents are assigned to distinguishable chemical shift ranges, as shown in Figure S31. A considerable number of resolvable signals within all of these ranges were seen. Findings suggest that a large number of distinct species are formed, presumably in a statistical manner, owing to the versatility of aldehyde chemistry. A multitude of signals in each chemical shift range suggests that all introduced reagent moieties are incorporated into more than one underlying species. Considering sample size and purity requirements, the applicability of postdigestion NMR was deemed poor in light of this outcome. While solid-state NMR may allow for direct study of modified MOF, it would require a more robust *ab initio* knowledge of the system. Therefore, NMR techniques were deemed prohibitively challenging for further inquiry in the present study. Other analyses were therefore pursued.

#### HRAM-ESI-MS: Dominant Ligand Modifications

Analysis by HRAM-ESI-MS is considered an apt tool for characterizing mixtures, especially if statistical product formation is involved. Key probed systems were chosen to be Model 2, and Digestion method 1 was applied to both Model 2 and the (*R*)-**1** aldehyde-treated UiO-66 NH_2_ product. Results are shown in Section S3.5.

Model 2 was chosen due to identical MOF modification conditions. An excess of unmodified ligands is added, representing the presumed excess in the postdigestion matrix. This presumption is based on partial conversion, found by bulk analysis methods. The literature inspired the digestion of zirconium MOF using aqueous carbonate solutions.^53^ The competitive coordination of carbonates allows for framework solubilization at hydrolytically milder pH régimes. Digestion method 1 was preferred, as Digestion method 2 was feared to release nucleophilic ammonia. Cesium cations were chosen to moderate matrix ionizability.

The 10 most intense signals were analyzed with primary, laxed, chemical bias. Corresponding hits are listed in Tables S12–S17, pairwise for the three respective systems. Corresponding hits are plotted in Figures S40–S42 for the three respective systems. The one hundred most intense signals were cross-referenced with masses conforming to a statistical set. Corresponding hits are listed in Tables S20–S25, pairwise for the three respective systems. These hits are referenced to the statistical set in Tables S26–S29. Referenced hits are plotted in Figures S45–S47 for the three respective systems. The statistical set was generated by acetonide hydrolysis, acetal, and oxomethylene chain formation, with hemiaminal adducts on imine permitted as applicable. Building blocks of the statistical set are listed in Table S18 and plotted in Figure S43. Constraints for building the statistical set are shown in Table S19, and the resulting set is plotted in Figure S44.

**Figure 5:**
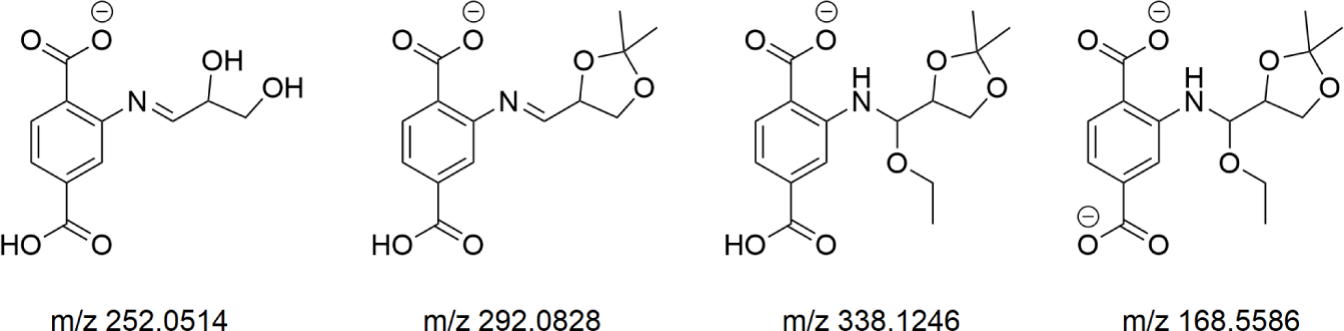
Ions, conforming to the constrained statistical product set, have been directly detected among the 100 strongest ESI-MS signals of the Digestion method 1 solution of the (*R*)-**1** aldehyde-treated UiO-66 NH_2_ substrate. This proves chemical nonuniformity.

Alongside the expected condensed imines, hemiaminals were found to be common in all mixtures. The ethanolous Model 2 solution was richer in dimeric adducts of the modifier, tethered predominantly via an oxomethylene backbone. The corresponding aqueous mock digestion revealed a shift to deoligomerization upon exposure to a new medium. Deacetonidation was, in turn, found to be the most prevalent in the MOF digestion product. The latter also had a distinctive prevalence of N-formylated substrate when analyzed without the constraints of the set. In Figure 5, representative species recovered directly from MOF digestion are shown.

Mass accuracy has allowed elucidation of the elemental composition upon the imposing of primary chemical constraints. In light of the stellar sensitivity of the method, cautious assessment is required. Potential external contaminants may not conform to the imposed primary constraints. Consequently, only interpretation of the most intense signals is warranted. As for signal intensity, the ionizability of species varies, although such may be presumed to be moderated by chemical similarity. In the present context, however, expected coordinative oligoether interactions with alkaline cations drew further caution.^54^ Analysis, therefore, required reserve with regard to prevalence to intensity correlativity. Positing which reactions govern presumed statistical product formation introduced further chemical bias. Representativeness of the model therefore needed to be validated.

The N-formylated substrate, found in the MOF digestion, may be assigned to transacylation from DMF during the MOF synthesis. This deactivation of the functionalization is a further limitation of the desired orthogonality in reactivities. However, the extent of the phenomena was assessed to be moderately consequential. Findings have validated the chemical model that arose from a preconception regarding dominant reaction types, as removing such bias reflected a coherent interpretation. Furthermore, the prevalence of the underlying reaction types was correlated to reaction conditions of interest. Importantly, analysis of the digestion sample gave direct evidence that the target species, the formal imine adduct, is characteristic of the modification of the MOF.

#### CD: Retention of Enantiomeric Excess

Solution CD was considered applicable, given that the species of interest can be solubilized. The technique relies on the differential absorption of circularly polarized light by an asymmetric pool of chromophore-bearing molecules. The presence of a chromophore is confirmed by absorbance for all three ethanolous model solutions. The cesium carbonate-containing Digestion method 1 medium has amply solubilized chromophores, while incomplete solubilization with the ammonium carbonate-containing Digestion method 2 yields a low chromophore concentration. As shown in Section S3.6, the presence of enantiomeric excess is clearly ascertained in Model 1 and Model 3 solutions but not for Model 2. Corresponding spectra are shown in Figures S48, S49, and S52, respectively.

A key limitation of the method was the required proximity of the chromophore to the chiral moiety, necessitating a positive control to validate the method. Furthermore, for successful measurement, absorbance needs to be fine-tuned with dilutions to optimize the signal-to-noise ratio, as both overdilution and poor transmission may be limiting. High absorbance backgrounds therefore may also have impeded the detection of enantiomeric excess. Detection of enantiomeric excess in the case of Model 1 and Model 3 experiments has demonstrated sufficient proximity of the chromophore to the chiral moiety. Given how Model 3 reaction conditions tightly match those of the MOF modification, the pertaining environment allowed for the retention of the enantiomeric excess. This can be interpreted as indirect evidence that modification of MOF does introduce an enantiomeric excess-bearing pool of appending auxiliaries.

Indication of enantiomeric excess, however, has not been deemed significant for any of the MOF digestion and mock digestion experiments. Corresponding spectra are disclosed for transparency in Figures S50, S51, and S53. As an enantiomeric excess was not clearly detected in any digestion experiments, direct evidence of chiralization was not obtained. Reflecting on the negative CD outcome of the Model 2 solution, the presumed culprit is a high absorbance background attributable to a large excess of the unmodified achiral ligand. Similarly, in digestion experiments, modest conversions would translate to such excess when modified MOFs are solubilized. Carbonate species-rich media may furthermore worsen background absorbance. The high pH of cesium carbonate solutions may also induce ulterior racemization upon incubation. Oligomerization upon modifier excess, paired with digestive deoligomerization, may also gauge CD outcome clarity via the Horeau effect.^55^

### Sterics of Modification, Grain, and Pore Structures

Here, we use a combination of computational techniques to model the attempted modifications. Hypothetical structures are built to address potential steric clashes in bulk-modified products and support their viability. Pore analysis assesses whether the bulk modification is accessible via a postsynthetic modifier penetration route.

#### *In Silico* Modeling: Stability of Bulk-Modified Products

Uniform appending of incorporated ligands is potentially limited by the steric confinement of the framework pores. Building *in silico* models with the modified ligands to address steric clashes filters out whether desired solid structures fall through on this criterion. The ligands, modified with either of the two chiral agents, have been incorporated into both frameworks *in silico*, with quantitative modifications. The modeling method is described in Section S4.1.

The structure was optimized using density functional theory (DFT) for UiO-66 NH_2_ modified with (*R*)-**1** aldehyde, further demonstrating that steric clashes do not destabilize the system. The optimization method is described in Section S4.2. It was found that this criterion may not rule out the desired uniform bulk modifications. However, observing the apparent hefty pore occupation of the introduced modifications urged the assessment of postsynthetic modifier penetration. Such is necessary as the permitted stability of the end product provides little to support the viability of the experimental route to obtain it.

#### Pore Analysis: Bulk Modification Postsynthetically Not Accessible

*In silico* pore analysis can reveal whether steric constraints prohibit the incorporation and internal displacement of a guest species within crystalline media. This is relevant for postsynthetic MOF modification, as contrasting the characteristic modifier radius and the largest free sphere radius within the substrate reflects the penetration of the former. Therefore, surface, shell, or bulk modification patterns may become discernible.

**Figure 6:**
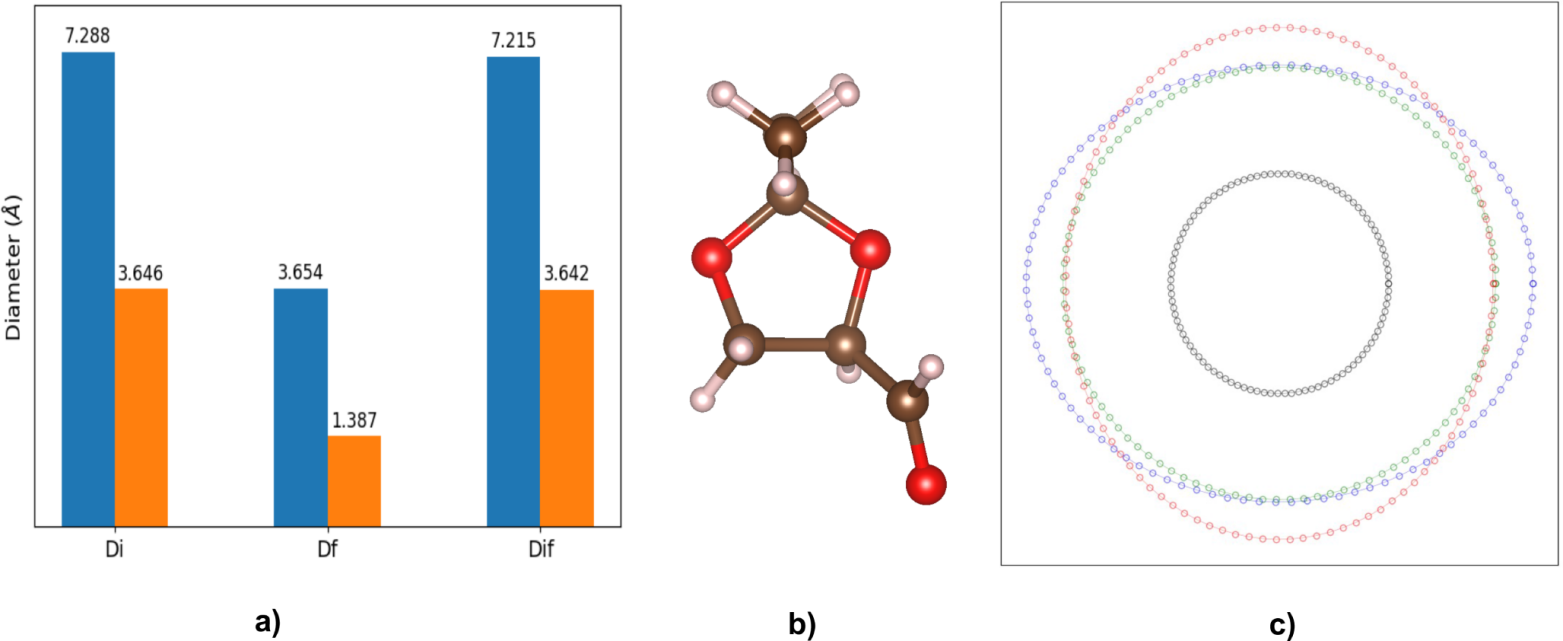
(a) Comparison of the pore diameters of the unmodified (blue) and modified (orange) MOFs. (b) Structure of the most stable conformer of the (*R*)-**1** aldehyde. (c) Visualization of the pore aperture of the unmodified MOF (black sphere) and axes of an ellipse encompassing the chiral molecule in different directions: *x* (blue), *y* (green), and *z* (red).

To gauge modifier penetration, pore analysis *in silico* was pursued for the flagship system, assessing both modified and unmodified UiO-66 NH_2_ substrates. The pore analysis method is described in Section S4.3, and the yielding diameters are shown in Table S30. The characteristic lengths of the (*R*)-**1** aldehyde modifying agent were ascertained for its most stable conformer, obtained from DFT optimization, as described in Section S4.2. The maximum length of the agent in three directions was computed and is 4.604 (*x*-direction), 5.656 (*y*-direction), and 4.689 Å (*z*-direction) (Figure 6). Since the agent is not spherical, drawing inspiration from the work of Ongari et al.,^56^ we considered drawing an ellipse with its major and minor axes being the plane in which it is studied. For example, if we look into the *x*-direction or *yz* plane, the ellipse will have its major axis as 5.656 Å and minor axis as 4.689 Å. Given the fact that the pore limiting diameter (Df) of the unmodified and modified substrate is 3.654 and 1.387 Å, respectively, we can say that even the unmodified substrate cannot fit the said conformer of the chiralizing agent. While dynamic equilibrium of conformers under reaction conditions is assumed, the extent of the misfit renders the results unequivocal.

Findings thus suggest that the obtained modification is predominantly relegated to the grain surface. Even defect-enabled self-limiting shell modification is presumably marginal, if at all relevant, considering how reaction speed may relate to that of Knudsen diffusion.^57^

Experimental results on the extent of relative organic mass incorporation and on the propensity of the modifier to oligomerize point to the formation of surface oligomers. Furthermore, given the size range of chiral analytes, the pore analysis’ findings translate to steric surface barrier formation in the context of prospective application in enantioseparation. This, in turn, limits diastereomeric interactions to the chiral oligomers on the surface, yielding an adsorption-based differentiation. In chromatographic separations, diffusion lengths within the stationary phase are highly consequential, as they determine the slope of the van Deemter curve in the ascending régime.^5,46^ Restricting interactions to the surface allows for a favorable separation speed to resolving-power trade-off in the parameter threshold relevant for applications.

## Conclusion

Chiralization of two amine-functionalized MOF substrates was undertaken through treatment with a chiral aldehyde and a chiral ketone. Respective controls with achiral agents were also performed. Thermogravimetric analyses of the relative organic mass have shown instances of incorporation upon these treatments. Elemental analyses have revealed these to be carbon-dominant. However, as for MIL-125 NH_2_, solvolytic substrate degradation was pronounced and even prohibitive at harsher reaction conditions. Ligand leaching was observed for MIL-125 NH_2_ in the presence of the free chromophore in the reaction solution. Noticeable yield reductions have stressed the extent of solvolytic degradation for this substrate. PXRD analyses confirmed, however, the retention of the structural backbone in studied solids. In conclusion, compositional analyses revealed a stability–reactivity window, constraining the accessibility of the surveyed chemical space. These findings gauge the scope applicability of this modification method, which is new to MOF chiralization. The UiO-66 NH_2_ (*R*)-**1** aldehyde system was therefore ascertained to be favorable in this study and became the focus of further inquiry. Other chiral oxo compounds for future work should be limited to aldehydes.

Different parameters can influence the covalent modification process. We studied the impact of the acidity of the catalyst, the nature of the carbonyl group, and the excess of the modifier. Increased acidity favors conversion but is undesirable for solvolytic stability and green chemistry. A more reactive carbonyl group allowed for milder reaction conditions. Excess modifier favors oligomerization. The explored reaction parameters give ample starting knowledge for future work to discover analogous materials and their more optimal and scaled synthesis. The reaction conditions identified hereby meet multiple criteria of green chemistry by being a mild catalytic process using natural-product-based modifiers in a green solvent.

Photographic, diffuse reflectance UV–Vis, and FT-IR spectral evidence of grain surfaces provided direct evidence of covalent modification. Exploratory NMR experiments, however, have alluded to a diverse set of resulting species. The HRAM-ESI-MS experiments detailed the underlying statistical product formation and demonstrated that the target modification is, nevertheless, characteristic. The CD measurements provided indirect evidence of enantiomeric excess in isolates, showing a retention of enantiomeric excess under process conditions. Target modification was, therefore, concluded to be achieved on the molecular level with the retention of enantiomeric excess. These findings are consequential as a proof of concept for the feasibility of this novel modification. Furthermore, a detailed description of this chemistry is key for the design of analogous systems.

Computational modeling has revealed that the product yielded from uniform bulk modification of the structure would be sterically stable. In *in silico*, pore analysis has shown the modifier’s pore penetration to be marginal, pointing to the dominance of surface modification, nuanced by highly self-limiting pore modification. Distribution of the modification was concluded to be predominantly limited to the surface, forming surface barriers. Reflecting on compositional and molecular-level experimental findings in light of pore analysis, we found that surface modification was rich in oligomers. These conclusions suggest that the obtained material is a favorable candidate for use as a stationary phase in chromatographic enantioseparation processes. These findings call for the prospective application of the new material in enantioseparations. Exploratory use of the material in a chromatographic setting is therefore suggested for subsequent future work. Furthermore, they serve as a systematic guide for the future discovery of similar materials. The present work can also serve as reference material for studying nonuniform modifications and their radial distribution in MOF.

## Supporting Information Available

Materials, methods, processed experimental data, and computational methods supporting this article have been included. Methods for MOF modification, homogeneous model experiments, digestion experiments, and a detailed description of characterization techniques. Processed experimental data: TGA, elemental analyses, SEM, photography, UV–Vis, FT-IR, NMR, HRAM-ESI-MS, and CD. Computational methods: models of prospective chiralized structures, the DFT optimization of structures and ligands, and pore analyses. The data for this article are available at Zenodo at 10.5281/zenodo.13305905 (PDF)
