# Supplementary material for: Nonuniform Chiralization of Metal–Organic Frameworks Using Imine Chemistry
Source: ACS Mater Au. 2025 Feb 4;5(3):491–501. doi: 10.1021/acsmaterialsau.4c00139 (PMC12082358; doi:10.1021/acsmaterialsau.4c00139)
Supplement: Supplementary file 1 — mg4c00139_si_001.pdf [file mg4c00139_si_001.pdf]

Supporting Information: Non-Uniform Chiralization of Metal-Organic Frameworks Using Imine Chemistry

Balázs Álmos Novotny,† Sauradeep Majumdar,† Andres Ortega-Guerrero,† Kevin Maik Jablonka,† Elias Moubarak,† Natalia Gasilova,‡ Nency P. Domingues,† Raluca-Ana Kessler,† Emad Oveisi,¶ Fatmah Mish Ebrahim,† and Berend Smit*,†

†*Laboratory of Molecular Simulation (LSMO), Institut des Sciences et Ingénierie Chimiques, Valais École Polytechnique Fédérale de Lausanne (EPFL), Rue de l'Industrie 17, CH-1951 Sion, Valais, Switzerland*

‡*Mass Spectrometry and Elemental Analysis Platform (MSEAP), Institut des Sciences et Ingénierie Chimiques, Valais École Polytechnique Fédérale de Lausanne (EPFL), Rue de l'Industrie 17, CH-1951 Sion, Valais, Switzerland*

¶*Interdisciplinary Center for Electron Microscopy (CIME), École Polytechnique Fédérale de Lausanne (EPFL), CH-1015 Lausanne, Switzerland*

E-mail: berend.smit@epfl.ch

Contents

1 Materials and methods **S3**

1.1 Materials S3

1.2 Methods for MOF modification S3

1.3 Homogenous model experiments S4

1.4 Digestion experiments S5

1.5 Characterization techniques S6

2 Bulk compositional inquiry **S8**

2.1 Expected thermolabile relative masses S8

2.2 Elemental analyses S19

2.3 Solution color: partial MOF dissolution S24

2.4 Yield: partial MOF dissolution S24

2.5 Scanning electron microscopy analyses S25

3 Molecular-level inquiry **S32**

3.1 Photographic analyses S32

3.2 Ultraviolet–visible spectroscopic analyses S37

3.3 Infrared spectroscopic analyses S40

3.4 Nuclear magnetic resonance analyses S43

3.5 High resolution accurate mass MS analyses S57

3.6 Circular dichroism analyses S76

4 Computational methods **S82**

4.1 Models of prospective chiralized structures S82

4.2 The DFT optimization of structures and ligands S82

4.3 Pore analyses S83

References **S84**

1 Materials and methods

1.1 Materials

For modification experiments, (R)-2,2-dimethyl-1,3-dioxolane-4-carboxaldehyde ((R)-**1** aldehyde) (50% in methylene chloride, 95%, 5 g) was purchased from Chemie Brunschwig AG, D-camphor (95%) from Fluorochem, acetaldehyde (puriss. p.a., anhydrous, 99.5% GC) from Sigma-Aldrich, acetone (p.a.) from Sigma-Aldrich, acetic acid (glacial, p.a.) from Merck Millipore, trifluoroacetic acid (99%) from SIAL, ethanol (p.a., absolute) from Fisher Chemical, and 2-aminoterephthalic acid (99%) from Sigma-Aldrich. For model experiments, deuterated methylene chloride (99.8% D) was purchased from Cambridge Isotope Laboratories, deuterated methanol (99.8% D, glass ampoule) from Chemie Brunschwig AG, dimethyl 2-aminoterephthalate (97%) from Fluorochem, and dichloromethane (anhydrous, 99.8%, contains 40-150 ppm amylene as stabilizer) from Sigma-Aldrich. For digestion experiments, cesium carbonate (99.9000% trace metals basis) was purchased from Sigma-Aldrich, and ammonium bicarbonate (99%) from SIAL. All chemicals were used as received, without further purification. The water for all experiments, Milli−Q purity, was obtained from Integral 5, Elix Technology machine by Merck Millipore. Compressed, on tap, nitrogen gas stemmed from liquid nitrogen (≥99.9%), supplied by Carbagas AG.

1.2 Methods for MOF modification

Each sample was treated in a plastic-capped 12 mL glass vial. For positive and negative controls, 10 mg of respective MIL-125 NH_2 or UiO-66 NH_2 MOF substrate was used. As for attempted chiralization, a 20 mg sample per vial was used. To each vial, 5 mL ethanol and 150 µL of glacial acetic acid were added. No modifying reagent was added for negative controls, while for positive controls, 300 µL of acetaldehyde, or 400 µL of acetone was added per vial. As for chiralization 300 mg of (R)-**1** aldehyde 50% solution, or 400 mg of D-camphor was added. For reaction with D-camphor, a free control, containing 30 mg of 2-aminoterephthalic

acid instead of the MOF was included. Ease of handling was reflected in weighing accuracy for reagents. All vials were capped thoroughly with parafilm under cap, and were then heated to 80 °C for 24 h. All samples containing D-camphor were supplemented with 150 µL of TFA when cold, following initial treatment, and were then recapped and returned for a second treatment for 24 h, at 100 °C this time. All thermal treatments were performed in a Carbolite programmable oven, inside a ventilated fume hood, model experiments included. Uniformly, heating rates of 2.0 °C min^{-1} and cooling rates of 0.2 °C min^{-1} were used, with a forced ramp not guaranteed upon cooling, for model experiments alike. Solids were washed once with 8 mL of ethanol per vial for positive and negative controls. When a chiral modifying agent was present, treated solids were washed with four times 10 mL of ethanol each, over the course of 3 days. Solids were aspirated upon washing and were collected by gravitational settling in original reaction vials, with supernatants removed by pipette. A minimum of 4-day ambient drying was ensured for each sample before analyses.

1.3 Homogenous model experiments

1.3.1 Model 1

Objective: Reproducing reaction in deuterated medium To an NMR tube, 15 mg of dimethyl 2-aminoterephthalate was added. A 750 µL ampoule of D4-methanol was taken with a glass pipette and flushed over (*R*)-**1** aldehyde 50% viscous solution, then transferred into the NMR tube. The capped NMR sample was treated at 70 °C for 20 h. Yielding condensed residue was redissolved in an identical, matching amount of fresh solvent. The obtained clear solution was stored at 4 °C.

1.3.2 Model 2

Objective: Reproducing reaction under deoligomerization favoring conditions To a 12 mL glass vial, 500 mg of 2-aminoterephthalic acid and 8 mL of ethanol were added. A 500 µL of methylene chloride was taken with a glass pipette and flushed over (*R*)-**1** aldehyde 50%

viscous solution cold, then transferred into the vial. The plastic cap was fit tight without parafilm, and the mixture was treated at 80 °C for 24 h. A sample of the resulting clear solution was taken using a glass pipette without disturbing the settled-out solid and stored at 4 °C.

1.3.3 Model 3

Objective: Reproducing reaction under the conditions of solid-phase treatment To a 12 mL glass vial, 20 mg of dimethyl 2-aminoterephthalate, 5 mL of ethanol, and 150 µL of glacial acetic acid were added. A 300 µL of (R)-**1** aldehyde 50% was transferred into the vial. The plastic cap was fit tight using parafilm, and the mixture was treated at 80 °C for 24 h. A sample of the clear solution was drawn using a glass pipette, and stored at 4 °C.

1.4 Digestion experiments

1.4.1 Digestion method 1

Objective: Solubilization in nucleophilic ammonia-free aqueous medium Solid analyte consisted of either 10 mg of treated MOF or a 400 µL residue of Model 2 solution obtained by drying with on-tap nitrogen gas flow. For each sample, digestion solutions were prepared by dissolving 100 mg of cesium carbonate in 650 µL of water in a glass vial. The digestion media were pipetted to respective analytes, and samples were mixed gently by tilting in capped glass vials. After some minutes, another 350 µL fresh digestion solution of identical composition was pipetted to each sample. Gentle mixing continued for another 0.5 h, and a minimum total digestion time of 24 h elapsed before analyses. Pertaining drying and digestion took place at ambient temperature. The completion of digestion was verified before analyses by checking the solution clarity and for lack of sedimentation.

1.4.2 Digestion method 2

Objective: Solubilization in pH-neutral aqueous medium Treated MOF analyte, 5 mg, was suspended in 700 µL of water for easy handling by pipette. A near-saturated aqueous ammonium bicarbonate solution was freshly prepared from the solid salt. Yielding liquids were mixed in various ratios and were vigorously aspirated for up to 10 min. The remaining solids were collected by 1 min of centrifugation at 2374 RCF in GmCLab Gilson Capsulfuge PMC-880, rendering digestion partial. Clear supernatants were removed by pipette and were subjected to immediate measurements. The procedure was performed at ambient temperature using plastic labware.

1.5 Characterization techniques

Weighing was done on a Mettler Toledo XP205 balance. Thermogravimetric analysis (TGA) traces were obtained on NETZSCH TGA209 F1 Libra instrument. A 10 mL min^{-1} airflow and a 20 mL min^{-1} protective nitrogen gas flow were employed. Samples were measured from ambient temperature to 700 °C, using a ramp speed of 5 °C/min. Alumina crucibles, 85 µL, NETZSCH GB399972, were used without lid, filled up to 15 mg of solid analyte, and calcined before use. Elemental analyses were performed on an Elementar Unicube instrument, employing an 1150 °C furnace and direct Temperature Programmed desorption. Samples being measured in triplicate. Powder X-ray diffractograms (PXRD) were accumulated on Bruker D8 Advance diffractometers, from 1.000° to 60.145° 2θ angle, using 2883 times 0.021° steps, 0.50 s per step. Measured at 25 °C, with 5.00 min^{-1} sample rotation. Generator with settings 40.0 kV, 40.0 mA, with Cu anode, and 0.2 µm Ni low $\kappa\beta$ filter used. The detector was LYNXEYE XE, 2.948° opening, ranges between 0.202 V and 0.232 V, with fixed slits, with both primary and secondary Soller slit at 2.500° limit. Low background Si sample holders from Bruker were used with 51.5 mm and 24.5 mm respective support and Si diameters. Scanning electron microscopy (SEM) images were acquired on a Thermo Fisher Scientific Teneo system at a beam energy of 1 keV and a beam current of 25 pA for the MIL-125 NH_2

figures, and a beam current of 50 pA for the UiO-66 NH_2 figures. Images were captured using the Trinity detector. Photography was performed in an illuminated ML-4030 Led Maxi Light Box with Datacolor Spyder CHECKR 24 color correction card, using the main camera of a Techno Camon 17 cell phone. White point color correction was done according to the reported procedure.[1] Ultraviolet–visible (UV-Vis) diffuse reflectance spectra were obtained using a PerkinElmer Lambda 850 UV/Vis Spectrometer. The diffuse reflectance was collected by compacting the powders into an appropriate sample holder. Fourier-transform infrared spectroscopy (FT-IR) was performed on Spectrum Two™ from PerkinElmer, with background subtractions, using direct measurement from treated MOF powders. All nuclear magnetic resonance (NMR) experiments were performed with a Bruker (AV-III) spectrometer equipped with a 5 mm BBO probe head capable of producing magnetic field pulse gradients in the z-direction of 54 G·cm^{-1}. Frequencies are 400.13 MHz for ^{1}H NMR and 100.62 MHz for ^{13}C NMR experiments. Standard Bruker parameters set were used for magnitude mode COSY (cosygpppqf), edited HSQC (hsqcedetgpissp2.3), and HMBC (hmbcgplpndqf). The Bruker DOSY pulse sequence (ledbpgp2s) was used to measure diffusion coefficients. The DOSY diffusion time interval (d20) and gradient pulse length (p30) were set at 1000 µs and 60 ms, respectively, with a recycle delay (d1) of 3 s. Each 1D free induction decay had 4K complex points with 32 scans averaged. The diffusion gradients were ramped from 2% to 98% at linear increments to generate 32 increments in the diffusion dimension. All experiments were performed at 298 K temperature, each taking about 50 min. DOSY NMR data processing was performed using MestreNova software. High-resolution accurate mass electrospray ionization mass spectra (HRAM-ESI-MS) were accumulated using an automated chip-based nanoelectrospray device (Triversa Nanomate, Advion, Ithaca, U.S.A.) coupled to Orbitrap Exploris 240 FT-MS instrument. Following calibration, samples were measured using direct injection in acetonitrile dilution in negative mode, and 0.1 V/V% formic acid containing acetonitrile dilution for positive mode. Mass spectra accumulated from m/z of 100 to m/z of 1000. The tandem mass spectrometric (ESI-MS/MS) experiment was performed in the same

setup with HCD fragmentation from m/z of 50 to m/z of 520. An internal calibration option EASY-IC was activated to achieve the highest possible mass accuracy (< 1 ppm). Circular dichroism (CD) spectra were obtained on a Chirascan V100 spectropolarimeter, with samples being held in a quart cuvette of 1 mm path length. Necessary dilutions were performed with respective neat solvents. Data yielding from the here described characterization techniques was organized and in part analyzed using the eln.epfl.ch platform.[2]

2 Bulk compositional inquiry

2.1 Expected thermolabile relative masses

$$m_{r,j} = \frac{M_{structure,j}}{M_{calcined,j}} \tag{1}$$

A relative mass for $\forall j$ may be defined by the ratio of the molar mass for the unit formula, to that of the corresponding calcined metal oxide, as shown in Equation (1).

$$\Delta m_{r,j} = \frac{M_{modified,j} - M_{unmodified,j}}{M_{calcined,j}} = \frac{M_{modification,j}}{M_{calcined,j}} \tag{2}$$

A relative mass increment for $\forall j$ may be defined using the molar mass increment pertaining to the modification, as shown in Equation (2).

$$M_{structure,j}(700\,^{\circ}C) = M_{calcined,j} \tag{3}$$

At 700 °C Equation (3) is considered valid for $\forall j$, in the presence of O_2 as only the calcined metal-oxide is retained.

$$m_{r,j,ex} = m_{r,j}(30\,^{\circ}C) \tag{4}$$

$$\Delta m_{r,j,ex} = \Delta m_{r,j}(30\,^{\circ}C) \tag{5}$$

The expected values for $m_{r,j}$ and $\Delta m_{r,j}$ may be calculated at the near ambient starting temperature of the thermogravimetric measurement, as shown in Equation (4) and Equation (5) respectively. Such posits that the formulae are representative at this temperature, which is limited by the underlying *ab initio* assumption of full conversion.

$$m_{r,j}(T) = \frac{m_j(T)}{m_j(700\,^{\circ}C)} \tag{6}$$

The experimental temperature dependence of $m_{r,j}$ may be found, as shown in Equation (6).

$$m_r(700\,^{\circ}C) = 1 \tag{7}$$

$$\Delta m_r(700\,^{\circ}C) = 0 \tag{8}$$

The endpoints of $m_r(T)$ and $\Delta m_r(T)$ curves are thereby fixed, as shown in Equation (7) and Equation (8) respectively.

Table S1: Determination of expected relative masses for MIL-125 NH_2 derived quantitatively imine modified structures and respective increments pertaining to each modification. For each structure and increment, a unit formula and a therefrom calculated molar mass are shown. Thereby obtained molar masses are normalized on that of the calcined metal oxide, to obtain expected relative masses according to Equation (4) and Equation (5) respectively.

	Formula	M [g/mol]	$m_{r,j,ex}$ [ ]
calcined metal oxide	O_8Ti_4	319.46	1.00
MIL-125 NH_2	$C_{24}H_{17}N_3O_{18}Ti_4$	826.87	2.59
acetaldehyde treated	$C_{30}H_{23}N_3O_{18}Ti_4$	904.98	2.83
(*R*)-**1** aldehyde treated	$C_{42}H_{41}N_3O_{24}Ti_4$	1163.25	3.64
acetone treated	$C_{33}H_{29}N_3O_{18}Ti_4$	947.06	2.96
D-camphor treated	$C_{54}H_{59}N_3O_{18}Ti_4$	1229.52	3.85
	Formula	M [g/mol]	$\Delta m_{r,j,ex}$ [ ]
acetaldehyde increment	C_6H_6	78.11	0.24
(*R*)-**1** aldehyde increment	$C_{18}H_{24}O_6$	336.38	1.05
acetone increment	C_9H_{12}	120.19	0.38
D-camphor increment	$C_{30}H_{42}$	402.65	1.26

Table S2: Determination of expected relative masses for UiO-66 NH_2 derived quantitatively imine modified structures and respective increments pertaining to each modification. For each structure and increment, a unit formula and a therefrom calculated molar mass are shown. Thereby obtained molar masses are normalized on that of the calcined metal oxide, to obtain expected relative masses according to Equation (4) and Equation (5) respectively.

	Formula	M [g/mol]	$m_{r,j,ex}$ [ ]
calcined metal oxide	$O_{12}Zr_6$	739.34	1.00
UiO66 NH_2	$C_{48}H_{34}N_6O_{32}Zr_6$	1754.15	2.37
acetaldehyde treated	$C_{60}H_{46}N_6O_{32}Zr_6$	1910.37	2.58
(*R*)-**1** aldehyde treated	$C_{84}H_{82}N_6O_{44}Zr_6$	2426.9	3.28
acetone treated	$C_{66}H_{58}N_6O_{32}Zr_6$	1994.53	2.70
D-camphor treated	$C_{108}H_{118}N_6O_{32}Zr_6$	2559.45	3.46
	Formula	M [g/mol]	$\Delta m_{r,j,ex}$ [ ]
acetaldehyde increment	$C_{12}H_{12}$	156.22	0.21
(*R*)-**1** aldehyde increment	$C_{36}H_{48}O_{12}$	672.76	0.91
acetone increment	$C_{18}H_{24}$	240.38	0.33
D-camphor increment	$C_{60}H_{84}$	805.31	1.09

Resulting plots are shown in the following order. Figures S1, S3, S4, and S6 pertain to MIL-125 NH_2, while Figures S2, S5, and S7 to UiO-66 NH_2 substrate. The treating oxo compounds were acetaldehyde for Figures S1, and S2, (R)-**1** aldehyde for Figure S3, acetone for Figures S4, and S5, and D-camphor for Figures S6, and S7.

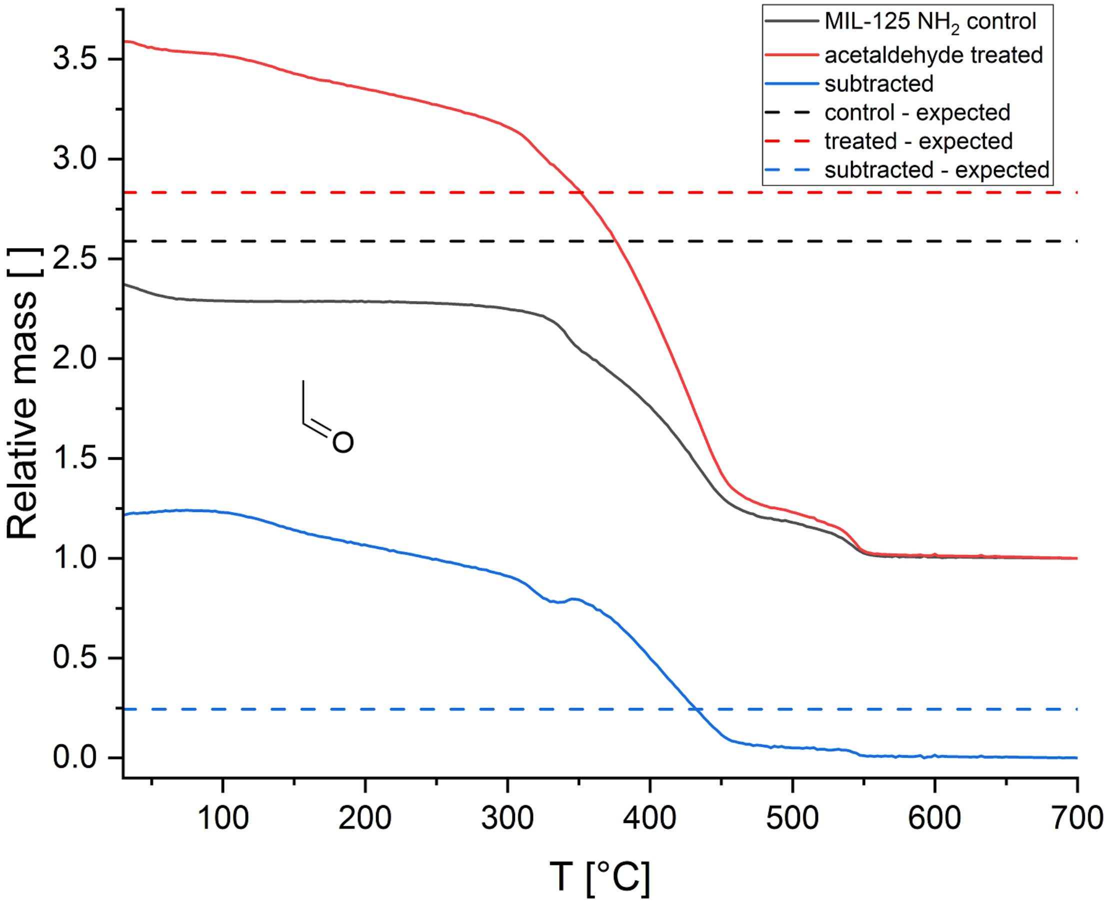

Figure S1: The thermogravimetric traces normalized on the calcined residues. Respective traces for treated substrate, control, and their difference are shown. Corresponding ***expected*** thermolabile relative masses, based on the stoichiometry of full conversion and complete desorption of potential guest molecules, are projected across the temperature series. Plot pertains to MIL-125 NH_2 substrate treated with oxo compound acetaldehyde.

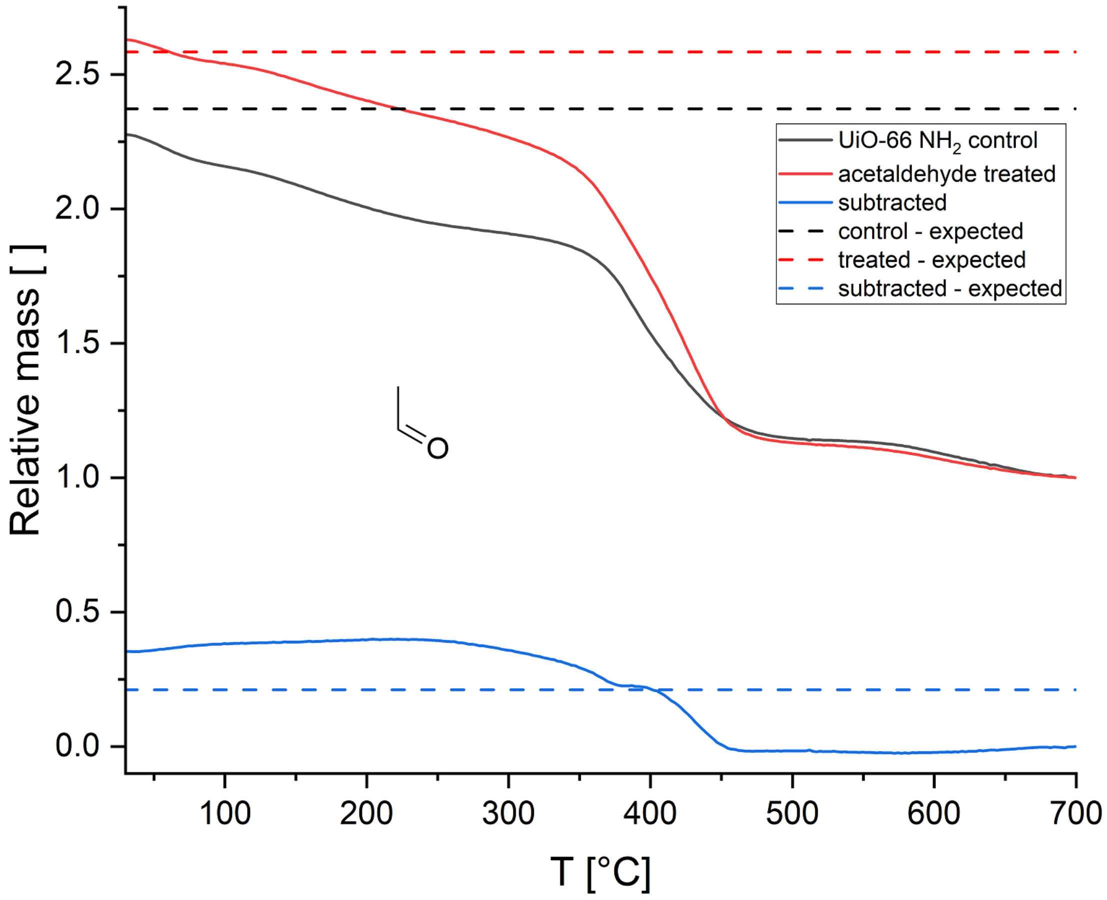

Figure S2: The thermogravimetric traces normalized on the calcined residues. Respective traces for treated substrate, control, and their difference are shown. Corresponding ***expected*** thermolabile relative masses, based on the stoichiometry of full conversion and complete desorption of potential guest molecules, are projected across the temperature series. Plot pertains to UiO-66 NH_2 substrate treated with oxo compound acetaldehyde.

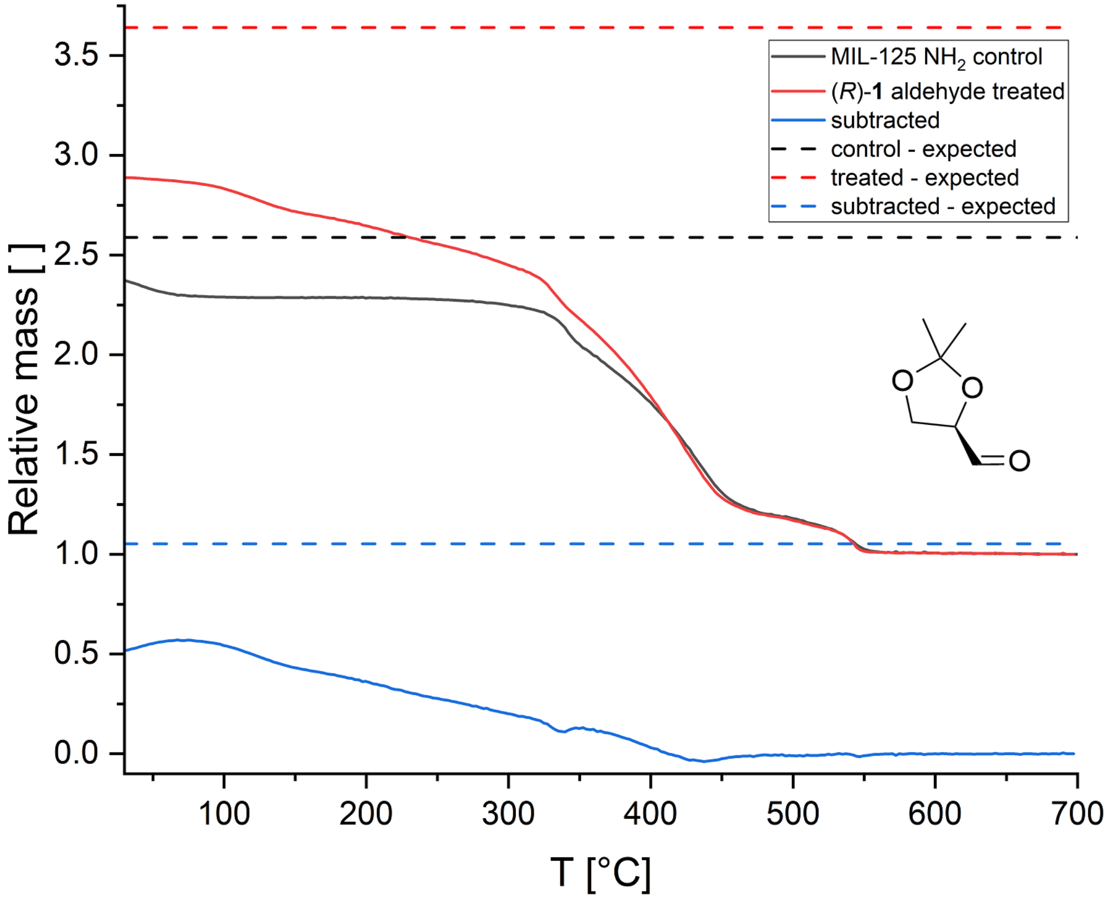

Figure S3: The thermogravimetric traces normalized on the calcined residues. Respective traces for treated substrate, control, and their difference are shown. Corresponding ***expected*** thermolabile relative masses, based on the stoichiometry of full conversion and complete desorption of potential guest molecules, are projected across the temperature series. Plot pertains to MIL-125 NH_2 substrate treated with oxo compound (*R*)-**1** aldehyde.

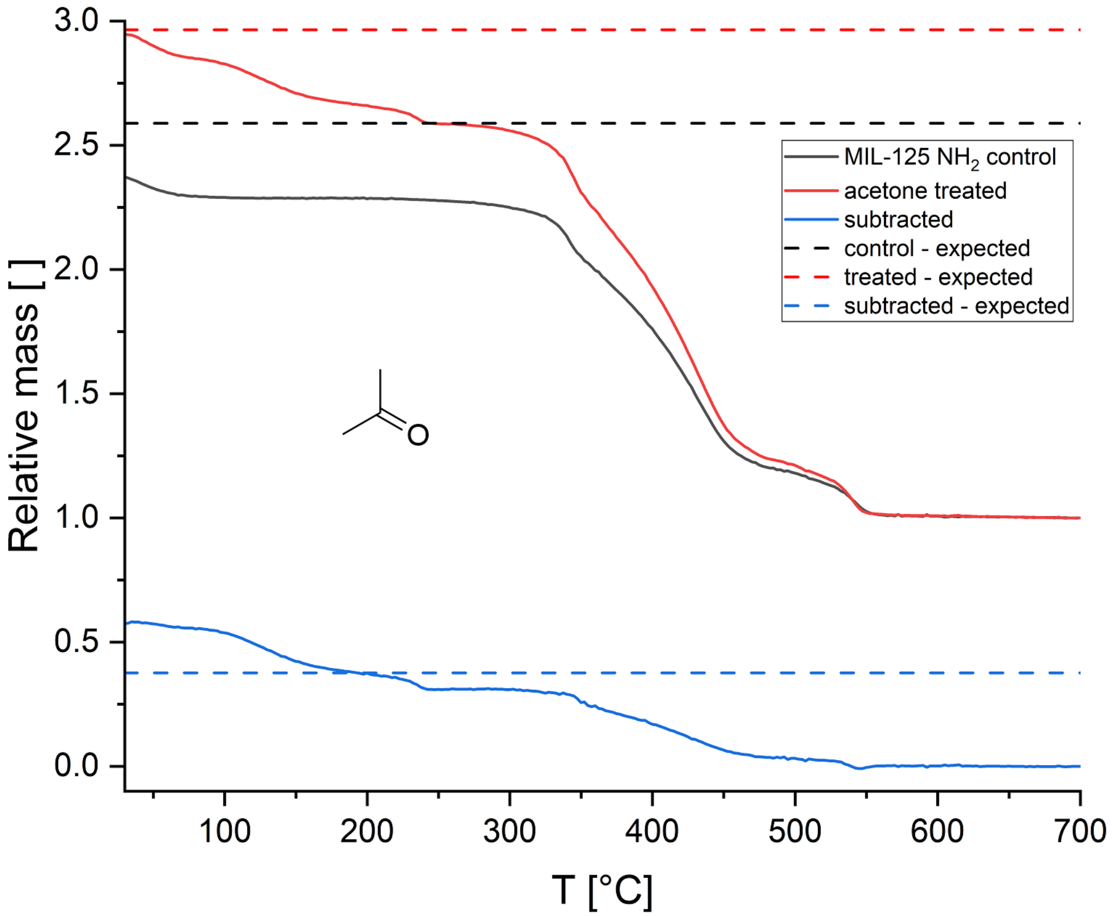

Figure S4: The thermogravimetric traces normalized on the calcined residues. Respective traces for treated substrate, control, and their difference are shown. Corresponding ***expected*** thermolabile relative masses, based on the stoichiometry of full conversion and complete desorption of potential guest molecules, are projected across the temperature series. Plot pertains to MIL-125 NH_2 substrate treated with oxo compound acetone.

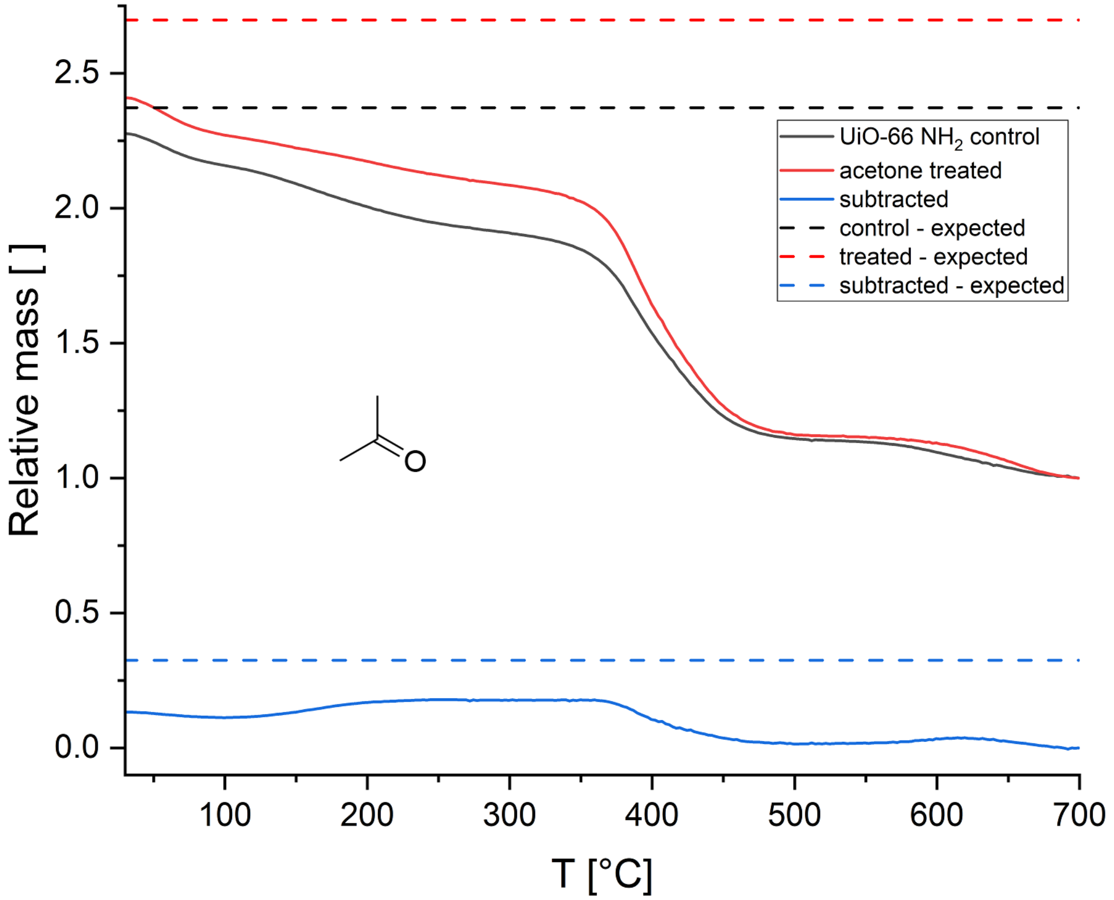

Figure S5: The thermogravimetric traces normalized on the calcined residues. Respective traces for treated substrate, control, and their difference are shown. Corresponding ***expected*** thermolabile relative masses, based on the stoichiometry of full conversion and complete desorption of potential guest molecules, are projected across the temperature series. Plot pertains to UiO-66 NH_2 substrate treated with oxo compound acetone.

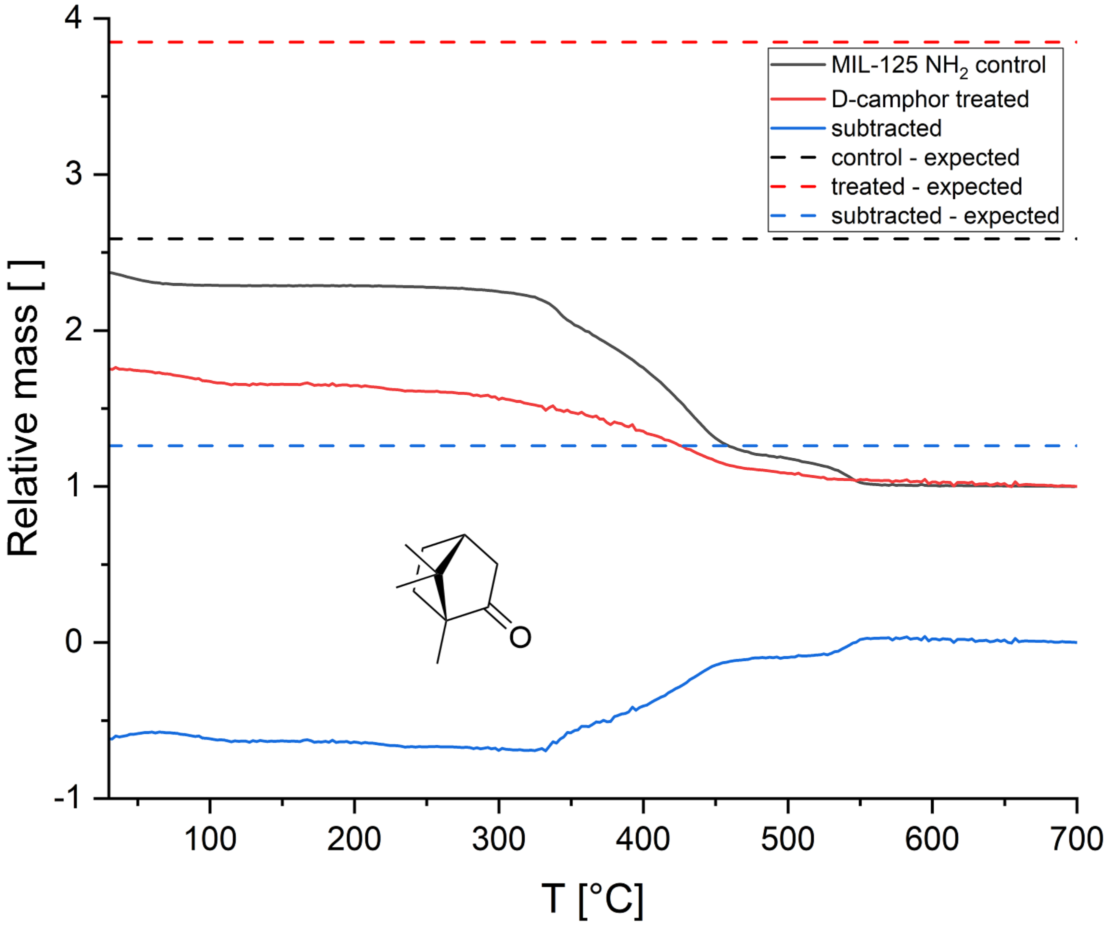

Figure S6: The thermogravimetric traces normalized on the calcined residues. Respective traces for treated substrate, control, and their difference are shown. Corresponding ***expected*** thermolabile relative masses, based on the stoichiometry of full conversion and complete desorption of potential guest molecules, are projected across the temperature series. Plot pertains to MIL-125 NH_2 substrate treated with oxo compound D-camphor.

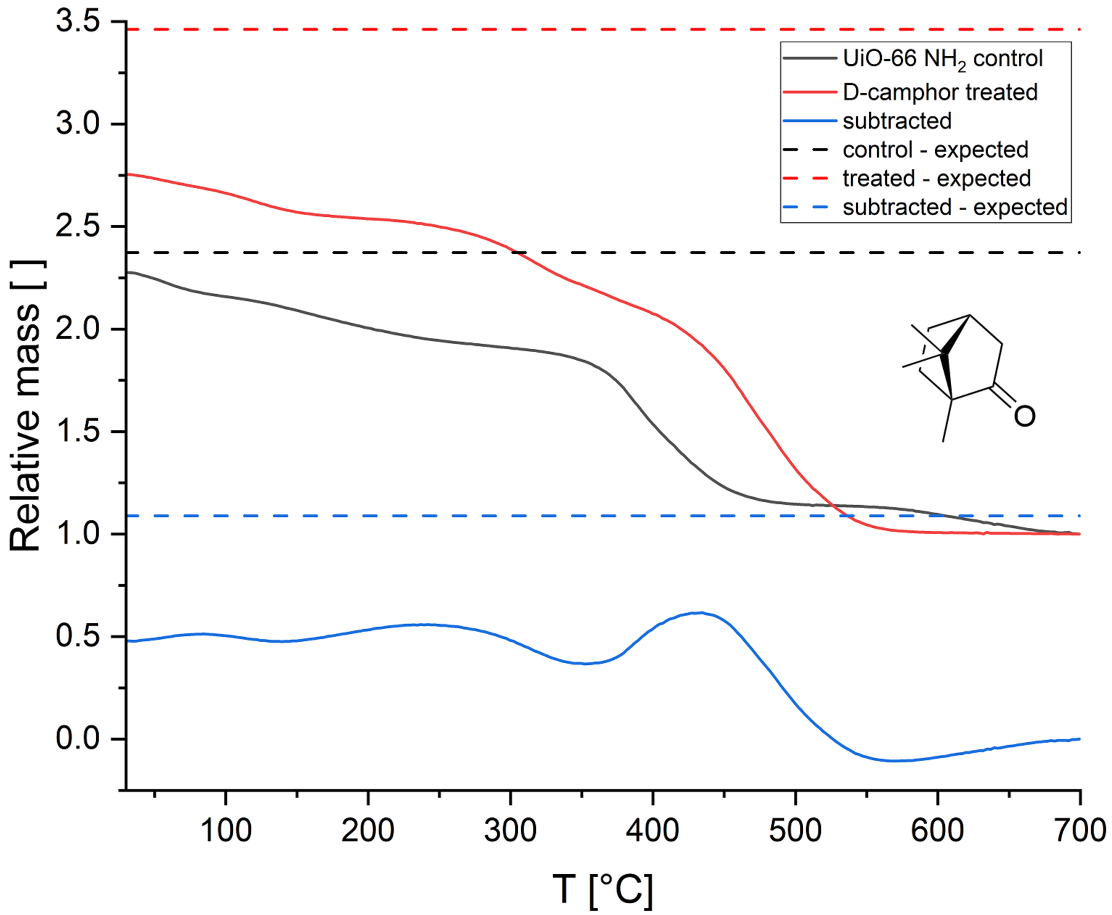

Figure S7: The thermogravimetric traces normalized on the calcined residues. Respective traces for treated substrate, control, and their difference are shown. Corresponding ***expected*** thermolabile relative masses, based on the stoichiometry of full conversion and complete desorption of potential guest molecules, are projected across the temperature series. Plot pertains to UiO-66 NH_2 substrate treated with oxo compound D-camphor.

A potential caveat to the initially outlined facile assessment may be the solvolytic degradation of MOF, leading to a simultaneous decrease of thermolabile relative mass via organic ligand leaching. Assuming that solvolytic degradation under otherwise identical reaction conditions is largely independent of the modifier present, such is accounted for by subtracting the traces of controls. Adding a further layer of complexity is the expected presence of physisorbed solvent for controls and attempted modifications. The thermolabile relative mass decay profile may be telling when it comes to discerning physisorbed solvents from covalent modifications. While the former is expected to be gradually released at lower temperatures, the latter is expected to be retained until the oxidative collapse of the framework. Despite such elaborations, the method has key limitations. The physisorbed solvent retention may improve considerably if the modification results in pore closure in the grain shell. Interface modification may also greatly impact physisorbed solvent uptake and retention. Furthermore, as the method only infers the strength of the interaction, incorporating the extended modifier as a guest may not be discernible from covalent binding.

2.2 Elemental analyses

Table S3: The calculated CHN elemental composition of MIL-125 NH_2 starting material and derived quantitatively imine modified expected structure. Established unit formulae, and monoelemental subformulae, therefrom calculated molar masses, and pertaining elemental mass fractions are shown.

	MIL-125 NH_2 starting material	C	H	N
Formula	$C_{24}H_{17}N_3O_{18}Ti_4$	C_{24}	H_{17}	N_3
M [g/mol]	826.87	288.26	17.13	42.02
Calculated (m/m%)		34.86	2.07	5.08
	(*R*)-**1** aldehyde treated	C	H	N
Formula	$C_{42}H_{41}N_3O_{24}Ti_4$	C_{42}	H_{41}	N_3
M [g/mol]	1163.25	504.45	41.32	42.02
Calculated (m/m%)		43.37	3.55	3.61

Table S4: The found CHN elemental composition of MIL-125 NH_2 starting material and derived modified structure. Results for three samples each and their averages are shown.

MIL-125 NH_2 starting material<br>Found (m/m%)	C	H	N
Sample 1	38.46	5.28	9.82
Sample 2	38.50	5.30	9.82
Sample 3	38.50	5.29	9.77
Average	38.49	5.29	9.80
(*R*)-**1** aldehyde treated<br>Found (m/m%)	C	H	N
Sample 1	39.71	4.33	4.43
Sample 2	39.49	4.36	4.42
Sample 3	39.55	4.36	4.41
Average	39.58	4.35	4.42

Table S5: Comparison of the calculated and average found CHN elemental composition of MIL-125 NH_2 starting material and derived modified structure.

		C	H	N
MIL-125 NH_2 starting material	Calculated (m/m%)	34.86	2.07	5.08
	Found (m/m%)	38.49	5.29	9.80
(*R*)-**1** aldehyde treated	Calculated (m/m%)	43.37	3.55	3.61
	Found (m/m%)	39.58	4.35	4.42

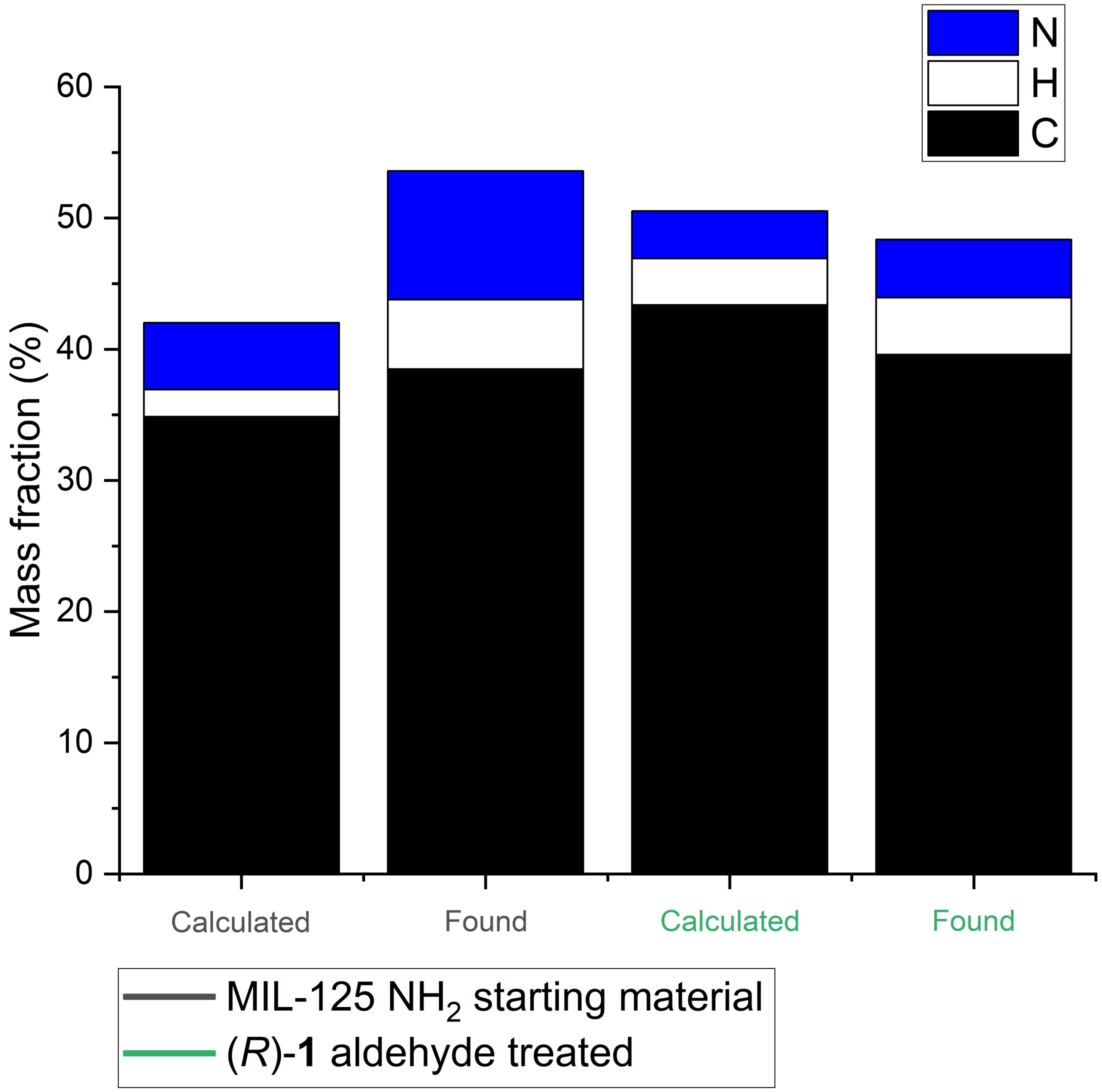

Figure S8: Graphical comparison of the calculated and average found CHN elemental composition of MIL-125 NH_2 starting material and derived modified structure.

Table S6: The calculated CHN elemental composition of UiO-66 NH_2 starting material and derived quantitatively imine modified expected structures. Established unit formulae, and monoelemental subformulae, therefrom calculated molar masses, and pertaining elemental mass fractions are shown.

	UiO-66 NH_2 starting material	C	H	N
Formula	$C_{48}H_{34}N_6O_{32}Zr_6$	C_{48}	H_{34}	N_6
M [g/mol]	1754.15	576.51	34.27	84.04
Calculated (m/m%)		32.87	1.95	4.79
	(R)-**1** aldehyde treated	C	H	N
Formula	$C_{84}H_{82}N_6O_{44}Zr_6$	C_{84}	H_{82}	N_6
M [g/mol]	2426.9	1008.9	82.65	84.04
Calculated (m/m%)		41.57	3.41	3.46
	D-camphor treated	C	H	N
Formula	$C_{108}H_{118}N_6O_{32}Zr_6$	C_{108}	H_{118}	N_6
M [g/mol]	2559.45	1297.16	118.93	84.04
Calculated (m/m%)		50.68	4.65	3.28

Table S7: The found CHN elemental composition of UiO-66 NH_2 starting material and derived modified structures. Results for three samples each and their averages are shown.

UiO-66 NH_2 starting material			
Found (m/m%)	C	H	N
Sample 1	25.93	4.05	3.91
Sample 2	25.97	3.99	3.92
Sample 3	25.91	4.01	3.94
Average	25.94	4.02	3.92
(R)-**1** aldehyde treated			
Found (m/m%)	C	H	N
Sample 1	30.92	3.57	3.20
Sample 2	30.97	3.59	3.21
Sample 3	30.76	3.62	3.19
Average	30.88	3.59	3.20
D-camphor treated			
Found (m/m%)	C	H	N
Sample 1	31.11	3.35	3.03
Sample 2	31.26	3.37	3.01
Sample 3	31.17	3.37	3.05
Average	31.18	3.36	3.03

Table S8: Comparison of the calculated and average found CHN elemental composition of UiO-66 NH_2 starting material and derived modified structures.

		C	H	N
UiO-66 NH_2 starting material	Calculated (m/m%)	32.87	1.95	4.79
	Found (m/m%)	25.94	4.02	3.92
(*R*)-**1** aldehyde treated	Calculated (m/m%)	41.57	3.41	3.46
	Found (m/m%)	30.88	3.59	3.20
D-camphor treated	Calculated (m/m%)	50.68	4.65	3.28
	Found (m/m%)	31.18	3.36	3.03

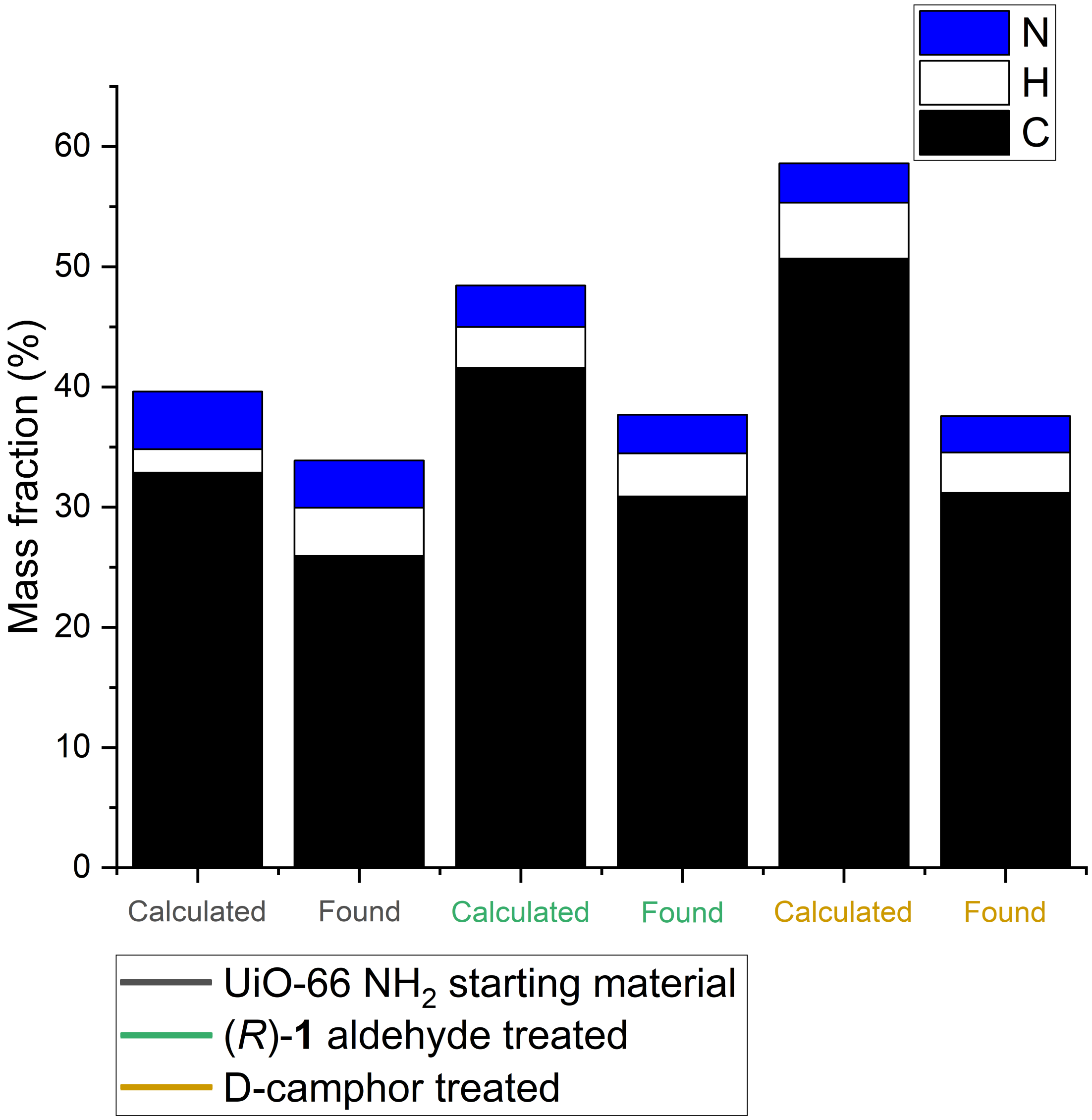

Figure S9: Graphical comparison of the calculated and average found CHN elemental composition of UiO-66 NH_2 starting material and derived modified structures.

Metal content ascertained via TGA may be used to assign unaccounted mass fraction to O content. Such analysis was, however, eluded due to inherent uncertainty regarding elemental exclusivity and methodological coherence.

2.3 Solution color: partial MOF dissolution

The orange color shift associated with the expected imine chromophore is an exceptional analytical asset, as it may be evaluated promptly without instrumentation. For UiO-66 NH_2, microcrystalline substrate settled slowly from solution. Nevertheless, the solution did not retain an orange tint upon eventual clearing. For MIL-125 NH_2, however, the orange coloration of particulate-free reaction mixtures was highly evident. Solution color may not only reveal the presence but also the localization of the product. Released MOF ligands in solution have an advantage for imine formation over bounded ones due to steric accessibility. Therefore, the reaction solution's orange tint is a robust indicator for ligand leaching. Earlier findings on solvolytic substrate stability were confirmed by the presence of the iminie chromophore, and thereby the presence of the leached ligand, in reaction mixtures. The UiO-66 NH_2 substrate was determined to perform considerably better with regard to ligand retention under relevant reaction conditions.

2.4 Yield: partial MOF dissolution

While the aforementioned analyses were discussed to reveal solvolytic substrate degradation, mass recovery may be regarded as the most direct indicator. For UiO-66 NH_2, isolated solids measured up, at minimum, to the mass of the starting substrate. However, for MIL-125 NH_2, solid masses were halved when subjected to modification conditions and decimated under the harsher conditions used with D-camphor. Given how desired modifications are incorporations, the mass of the isolated solid may unequivocally reveal substrate solubilization if it is inferior to the starting amount. Such observations have strongly confirmed and quantitatively nuanced the already established trends in solvolytic substrate stability.

2.5 Scanning electron microscopy analyses

2.5.1 Morphology of MIL-125 NH_2 samples

The SEM analysis was done to study the crystal morphology of MIL-125 NH_2 starting substrate and MIL-125 NH_2 negative control experiment isolate and see the impact of the reaction conditions without a modifier. As it can be seen in Figures S10 and S11 the crystals of MIL-125 NH_2 starting substrate have a rounded platelets morphology. Comparison of MIL-125 NH_2 starting substrate and MIL-125 NH_2 negative control experiment isolate (see Figures S12 and S13) reveals that while the overall crystal morphology remains the same, signs of solvolytic degradation on the surface appear. For MIL-125 NH_2 starting substrate the crystals have a smooth surface, which becomes rougher for MIL-125 NH_2 negative control experiment isolate. This observation is in line with found solvolytic degradation discussed in SI Section 2.1.

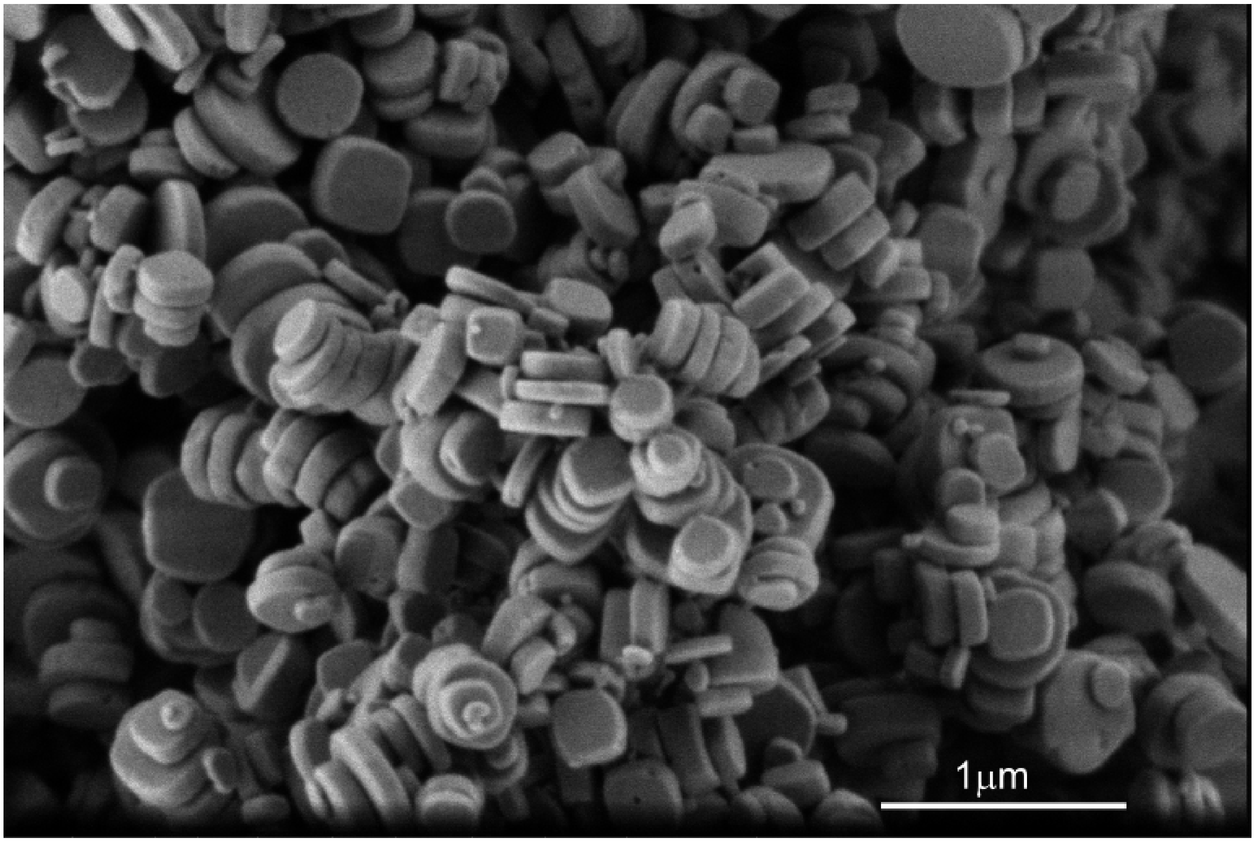

Figure S10: SEM image of MIL-125 NH_2 starting substrate.

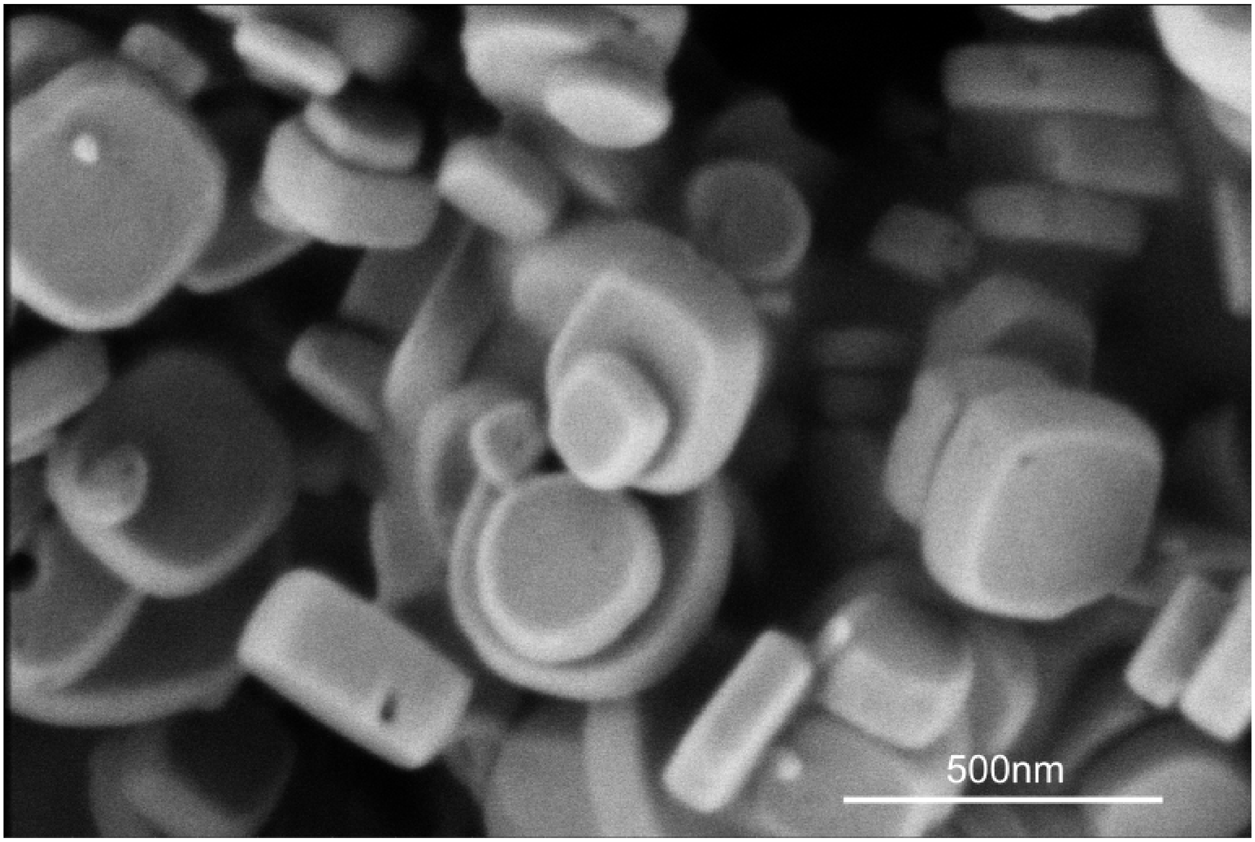

Figure S11: SEM image of MIL-125 NH_2 starting substrate.

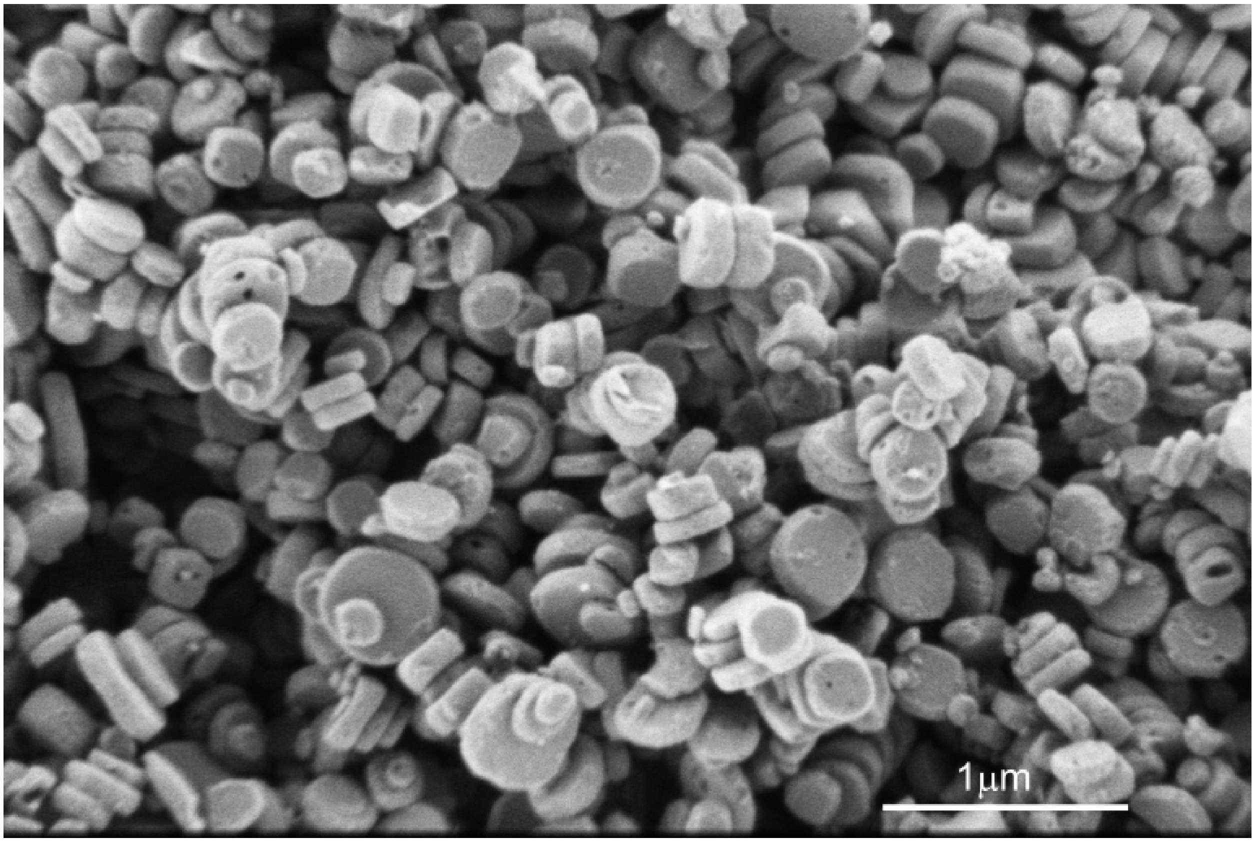

Figure S12: SEM image of MIL-125 NH_2 negative control experiment isolate.

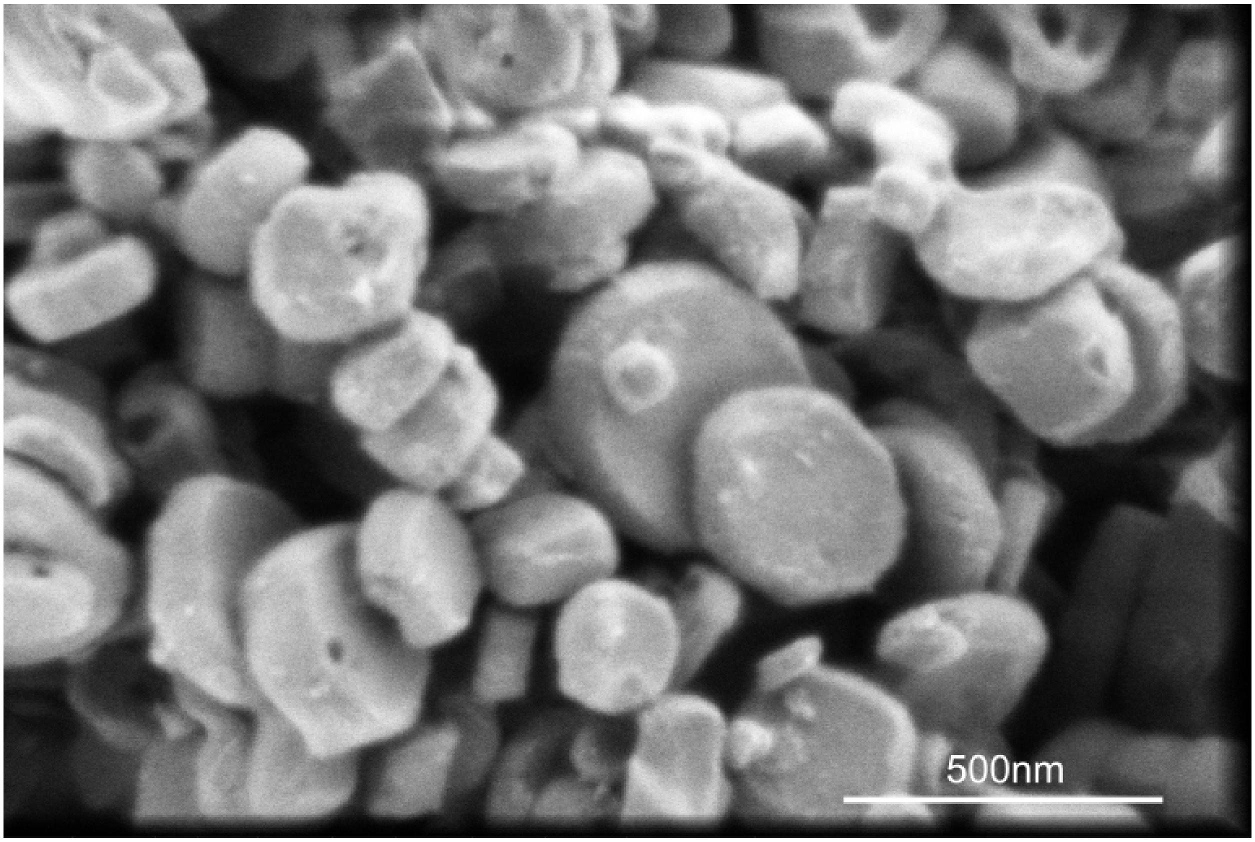

Figure S13: SEM image of MIL-125 NH_2 negative control experiment isolate.

2.5.2 Morphology of UiO-66 NH_2 samples

In this part, SEM was used to examine the morphology of UiO-66 NH_2 starting substrate, UiO-66 NH_2 negative control experiment isolate, and (R)-**1** aldehyde treated UiO-66 NH_2 isolate to study the effect of the reaction conditions in the absence and in the presence of a modifier. It can be observed in Figures S14, S15, and S16, that all samples show a matching intergrown crystals with hexagonal faces. Crystals of UiO-66 NH_2 negative control experiment isolates in Figure S15, appeared to be considerably affected by the reaction conditions in the absence of a modifier. For this sample, the edges of the crystals get rounded, attributable to pronounced solvolytic degradation. The SEM image of (R)-**1** aldehyde UiO-66 NH_2 isolate (Figure S16), however, shows more moderate signs of solvolytic degradation. These difference between the two samples can be explained by the surface modification doting resistance to the surface against solvolytic degradation.

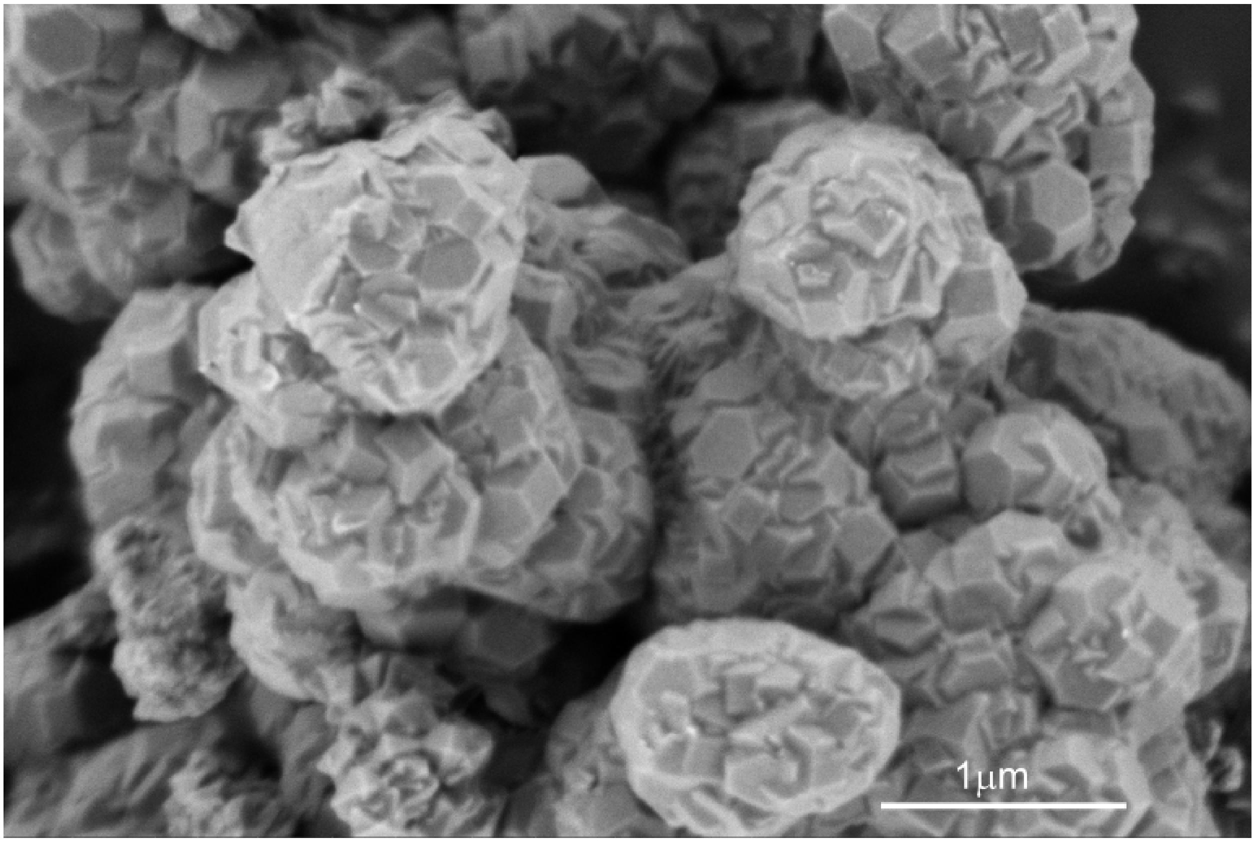

Figure S14: SEM image of UiO-66 NH_2 starting substrate.

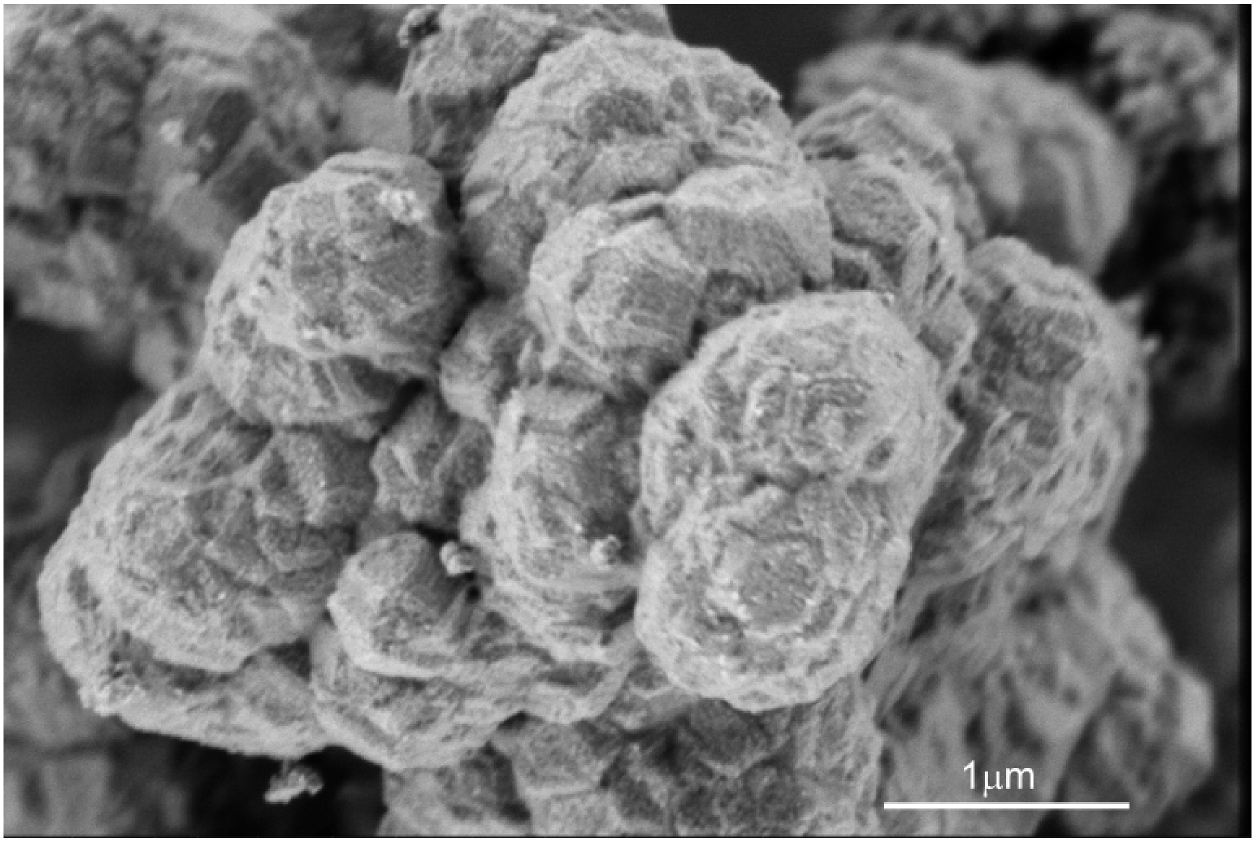

Figure S15: SEM image of UiO-66 NH_2 negative control experiment isolate.

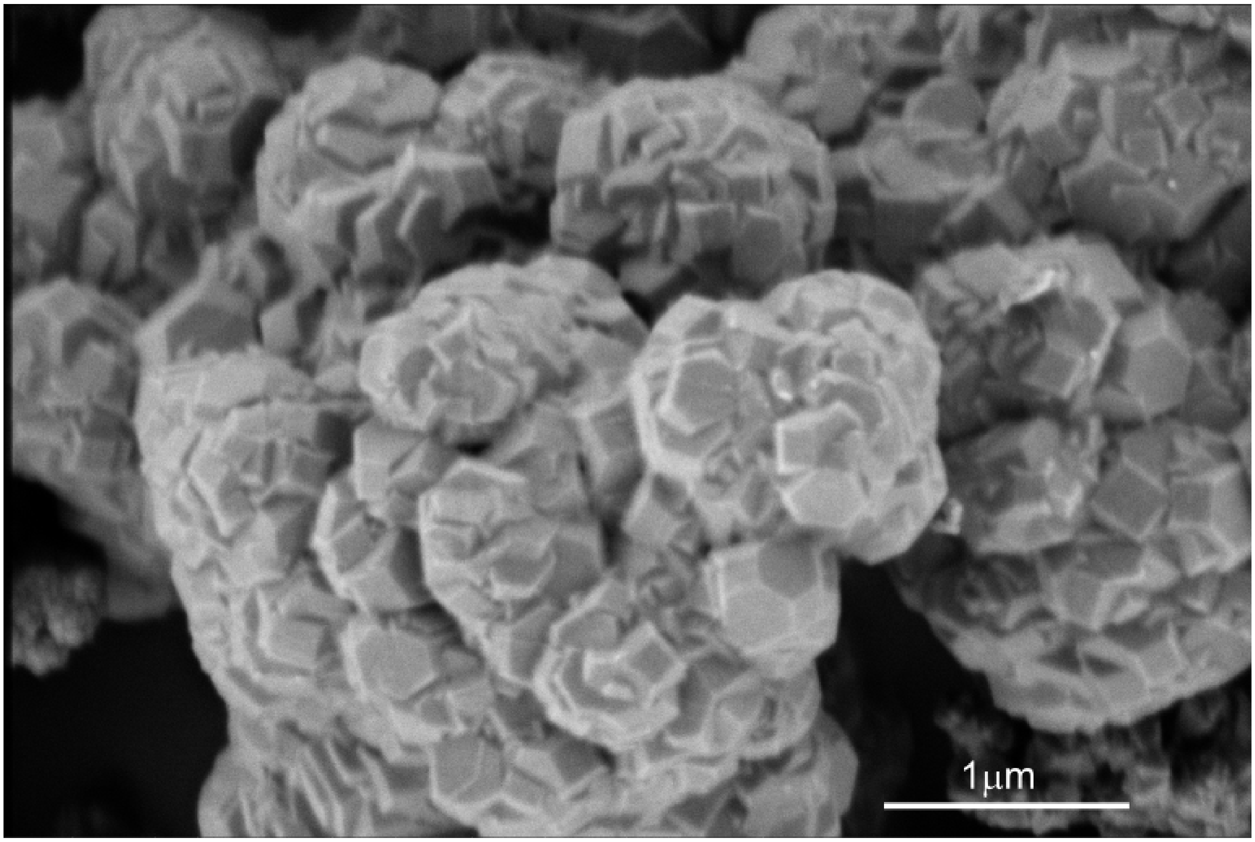

Figure S16: SEM image of (*R*)-**1** aldehyde treated UiO-66 NH_2 isolate.

3 Molecular-level inquiry

3.1 Photographic analyses

To validate the success of our syntheses, we also measured the color change between the starting materials and the Schiff-base product. To do so, we recorded photos with a color rendition chart, as reported by Jablonka et al.[1]

After gray-point correction, we measured the color distribution in a representative part of the powder sample. We then perform Hotelling t-squared tests[3,4] in both RGB and the visually more uniform LAB color space between the color distribution of the starting material and the product. We find $p = 0$ in all cases and reject the null hypothesis that both distributions have the same mean. We additionally quantify the distances using distances between histograms of RGB colors (as implemented in OpenCV[5]) shown in Table S9. The color-calibrated images are compared for MIL-125 NH_2 substrate in Figures S17, and S18, and for UiO-66 NH_2 substrate in Figures S19, S20, and S21. Figures S17, and S19 show starting materials, Figures S18, and S20 show (*R*)-**1** aldehyde treated substrates, and Figure S21 shows D-camphor treated substrate. The average rounded RGB sample colors, obtained from the color calibrated images, are shown in Table S10.

The code for the analysis can be found on GitHub (`https://github.com/kjappelbaum/chiral-mof-color-analysis`).

3.1.1 Metrics

Correlation

$$\mathrm{d}\left(\mathrm{H}_1, \mathrm{H}_2\right) = \frac{\sum_{\mathrm{I}} \left(\mathrm{H}_1(\mathrm{I}) - \overline{\mathrm{H}}_1\right)\left(\mathrm{H}_2(\mathrm{I}) - \overline{\mathrm{H}}_2\right)}{\sqrt{\Sigma_{\mathrm{I}} \left(\mathrm{H}_1(\mathrm{I}) - \overline{\mathrm{H}}_1\right)^2 \Sigma_{\mathrm{I}} \left(\mathrm{H}_2(\mathrm{I}) - \overline{\mathrm{H}}_2\right)^2}}$$

with

$$\overline{\mathrm{H}}_{\mathrm{k}} = \frac{1}{\mathrm{N}} \sum_{\mathrm{J}} \mathrm{H}_{\mathrm{k}}(\mathrm{J})$$

χ^2

$$d(H_1, H_2) = \sum_I \frac{(H_1(I) - H_2(I))^2}{H_1(I)}$$

Intersection

$$d(H_1, H_2) = \sum_I \min(H_1(I), H_2(I))$$

Bhattacharyya

$$d(H_1, H_2) = \sqrt{1 - \frac{1}{\sqrt{\bar{H}_1 \bar{H}_2}\, N^2} \sum_I \sqrt{H_1(I) \cdot H_2(I)}}$$

3.1.2 Results

Table S9: Histogram distances (see section 3.1.1 for definition of the distances) between colors sampled from images of treated and starting MOFs.

sample	correlation	χ^2	intersection	Bhattacharyya
MIL-125 NH_2 - (*R*)-**1** aldehyde	0.19	2817	117	0.85
UiO-66 NH_2 - (*R*)-**1** aldehyde	0.00	787	70	0.95
UiO-66 NH_2 - D-camphor	0.00	1102	23	0.98

Table S10: The average rounded RGB (Red-Green-Blue) sample color, obtained from withing the sampling rectangle of the respective color calibrated images.

sample	R	G	B
MIL-125 NH_2 - starting	185	184	162
MIL-125 NH_2 - (*R*)-**1** aldehyde	188	184	105
UiO-66 NH_2 - starting	185	180	167
UiO-66 NH_2 - (*R*)-**1** aldehyde	187	181	130
UiO-66 NH_2 - D-camphor	183	179	164

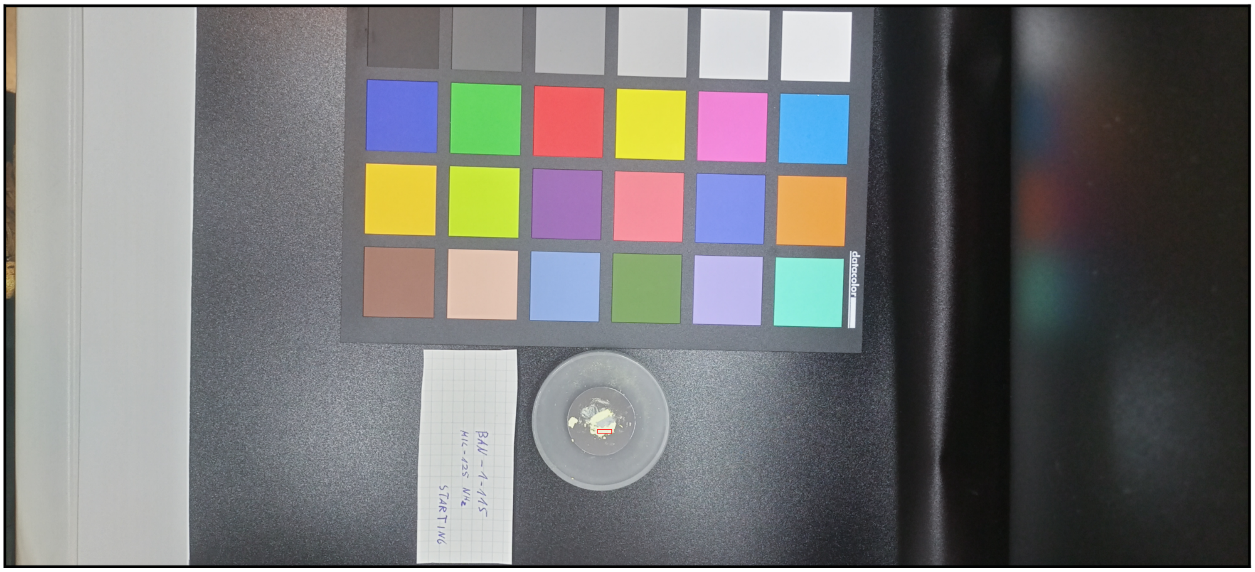

Figure S17: Color-calibrated image of MIL-125 NH_2 starting material shown. Region used for the analysis is highlighted with a red rectangle.

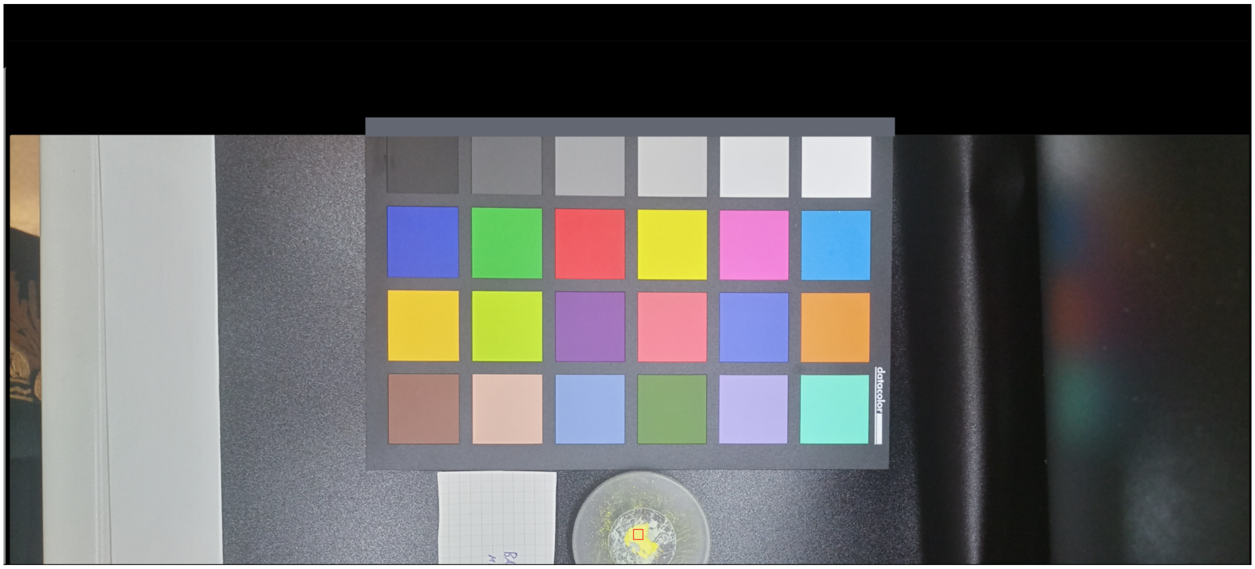

Figure S18: Color-calibrated image of (R)-**1** aldehyde treated MIL-125 NH_2 substrate shown. Region used for the analysis is highlighted with a red rectangle.

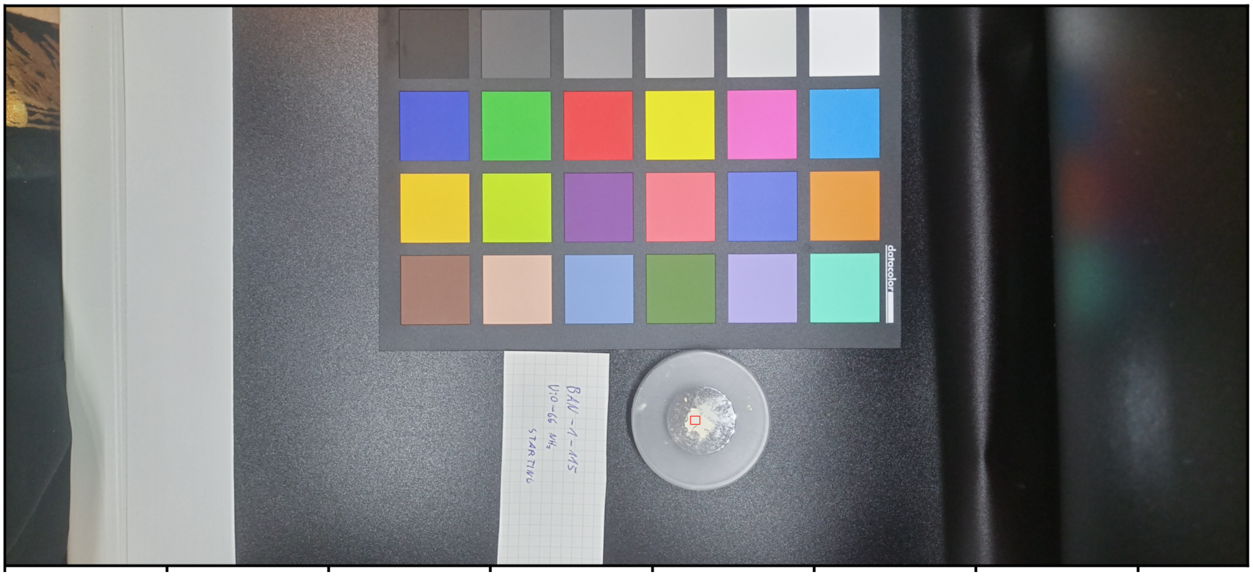

Figure S19: Color-calibrated image of UiO-66 NH_2 starting material shown. Region used for the analysis is highlighted with a red rectangle.

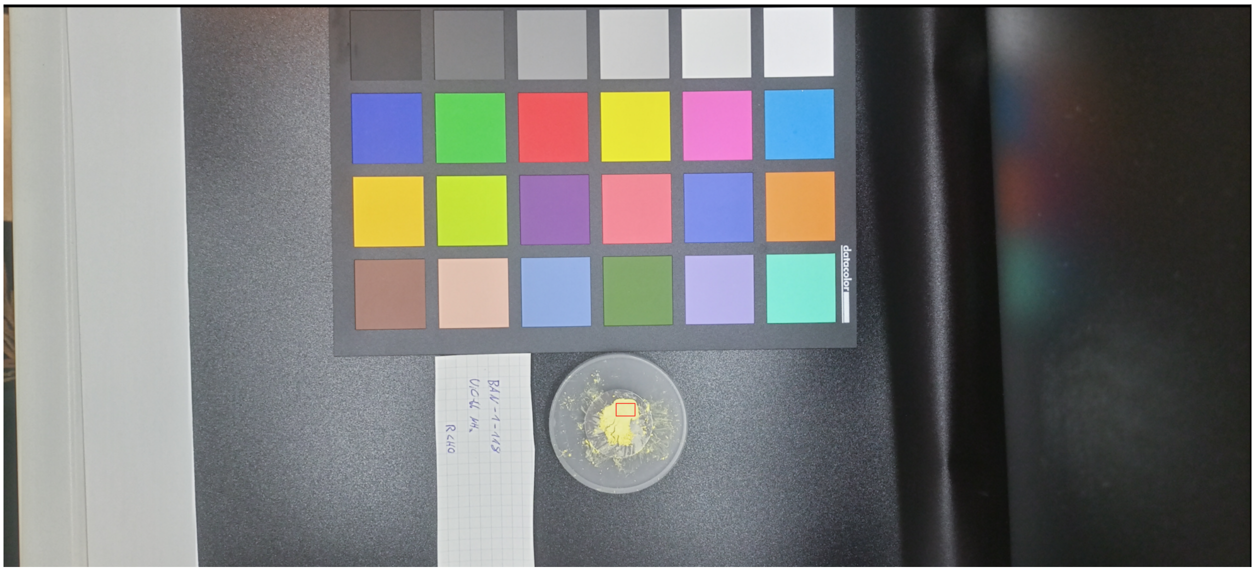

Figure S20: Color-calibrated image of (R)-**1** aldehyde treated UiO-66 NH_2 substrate shown. Region used for the analysis is highlighted with a red rectangle.

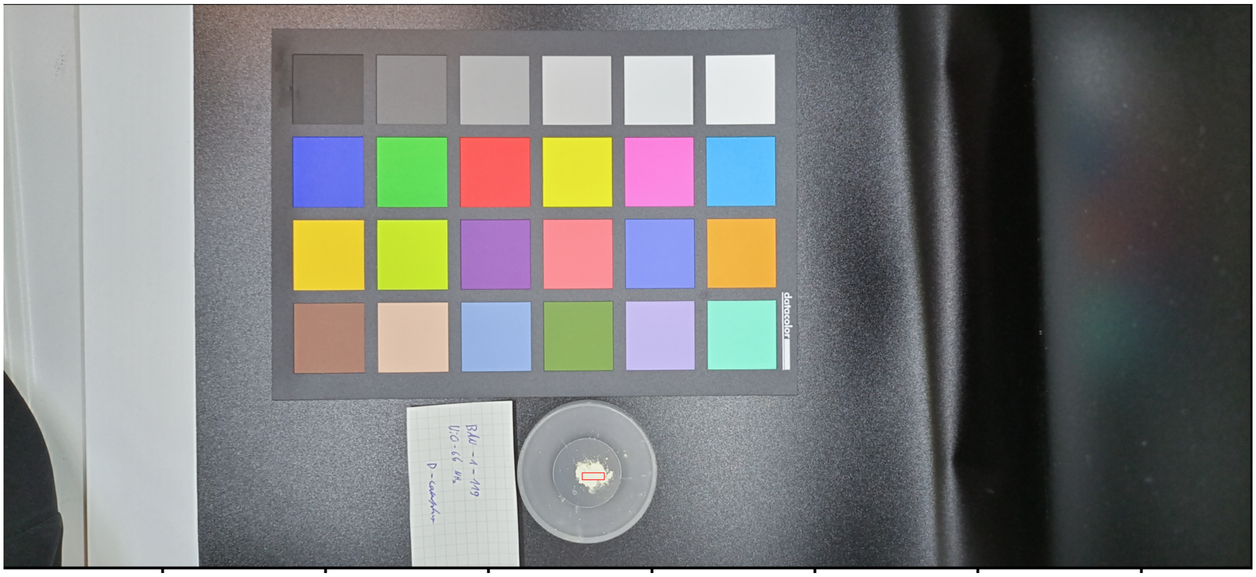

Figure S21: Color-calibrated image of D-camphor treated UiO-66 NH_2 substrate shown. Region used for the analysis is highlighted with a red rectangle.

3.2 Ultraviolet–visible spectroscopic analyses

The light absorption of the materials can be determined from the diffuse reflectance UV-Vis spectra, which were obtained using a PerkinElmer Lambda 850 UV/Vis Spectrometer. The diffuse reflectance spectra were collected by compacting the powders into an appropriate sample holder at room temperature.

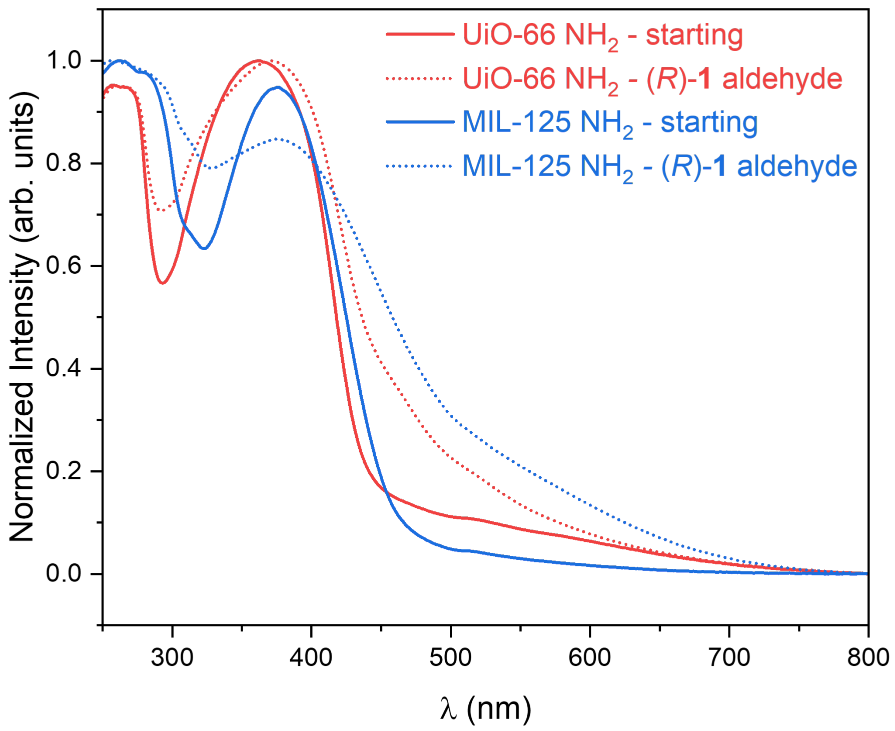

Figure S22: The UV-vis adsorption spectra in diffuse reflectance of the starting and (*R*)-**1** aldehyde treated UiO-66 NH_2 and MIL-125 NH_2.

The results show that both the starting materials and the post-synthetically modified materials exhibit a broad absorption peak in the 300 nm - 450 nm range. For UiO-66 NH_2, a slight shift towards higher wavelengths of the main absorption peak is observed following post-synthetic modification. This is further accompanied by an extension of the visible light absorption region. In contrast, for MIL-125 NH_2, the shift in the maximum absorption peak is negligible; however, the visible light absorption is further enhanced after modification.

3.2.1 Chromaticity Diagram

The chromaticity diagram was determined using the chromaticity diagram application available in the OriginPro software.[6] More specifically, the visible absorption spectra in diffuse reflectance were normalized to 1 (in the 380 nm - 700 nm range). The normalized absorption data was substracted from 1 (i.e., 1-A(λ)). Then, the OriginPro chromaticity diagram application was used to generate the CIE 1931 color coordinates (x, y). The data are available in Table S11, and these coordinates were then also mapped onto the CIE 1931 chromaticity diagram (spectral type: reflectance/transmittance, illuminant: D65 (daylight), observer: 2° standard observer) (Figure S23). For comparison with the photographic analysis, the previous RGB color codes have been converted to (x, y) CIE 1931 color coordinates using the equations provided below:[7–9]

$$\begin{aligned} X &= 0.4125 \cdot R + 0.3576 \cdot G + 0.1804 \cdot B \\ Y &= 0.2127 \cdot R + 0.7152 \cdot G + 0.0722 \cdot B \\ Z &= 0.0193 \cdot R + 0.1192 \cdot G + 0.9503 \cdot B \end{aligned} \tag{9}$$

and

$$\begin{aligned} x &= \frac{X}{X+Y+Z} \\ y &= \frac{Y}{X+Y+Z} \end{aligned} \tag{10}$$

Table S11: The (x, y) CIE 1931 color coordinates.

**Label**	**Color Coordinates Calculated from the visible spectra in Diffuse Reflectance**		**Color Coordinates Calculated from the Photographic Analysis**	
	**x**	**y**	**x**	**y**
UiO-66 NH_2 - starting	0.3255	0.3461	0.3195	0.3369
UiO-66 NH_2 - (*R*)-**1** aldehyde	0.3489	0.3750	0.3356	0.3626
MIL-125 NH_2 - starting	0.3294	0.3587	0.3212	0.3424
MIL-125 NH_2 - (*R*)-**1** aldehyde	0.3745	0.4036	0.3477	0.3838

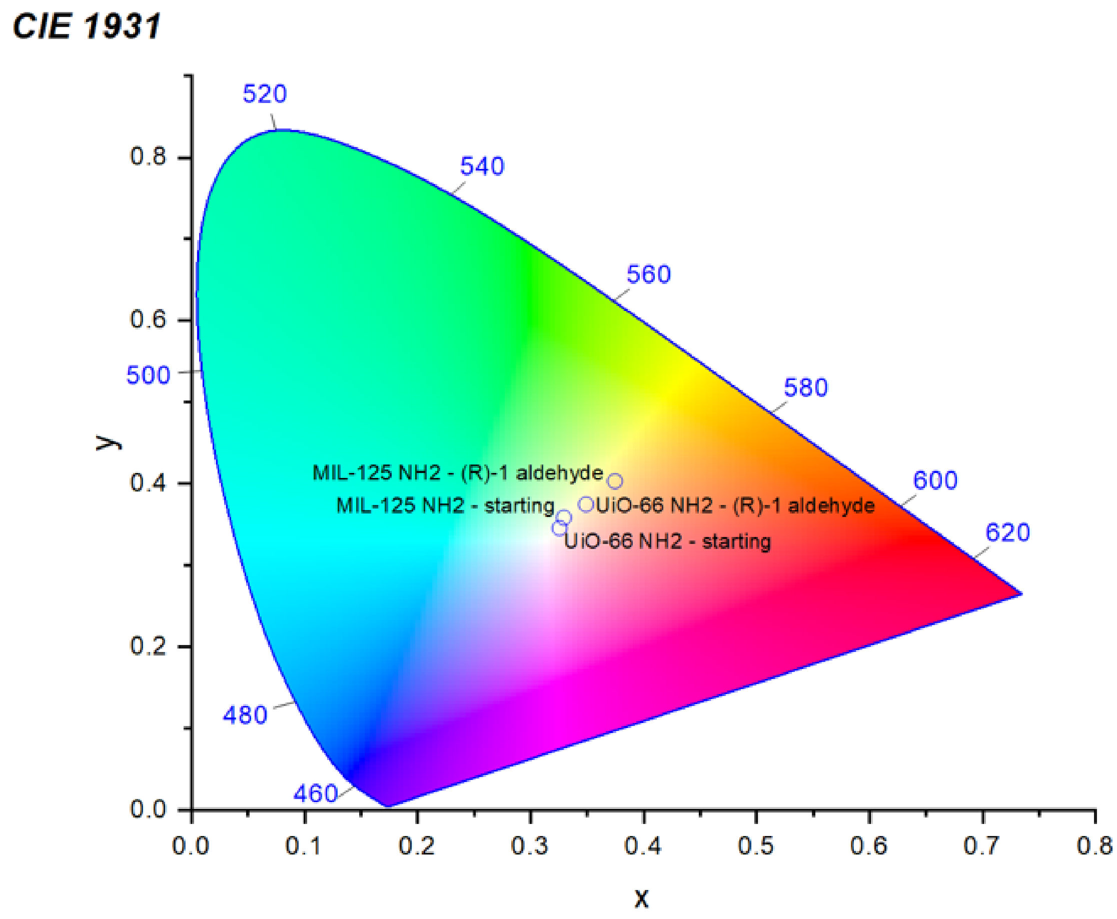

Figure S23: The CIE 1931 diagram of the starting and modified UiO-66 NH_2 and MIL-125 NH_2.

Compared to UiO-66 NH_2 and MIL-125 NH_2 starting materials, the (R)-**1** aldehyde treated samples are found more in the yellow/orange region of the plot (Figure S23), which is consistent with the photographic analysis described in SI Section 3.1. Moreover, the (x, y) color coordinates calculated from the visible absorption spectra in diffuse reflectance and the photographic analysis are also in good agreement.

3.3 Infrared spectroscopic analyses

The heterogeneity of the samples and the diversity of the species formed limits the robustness of the FT-IR analysis. Given the modest specificity of the method, identifying the imine band according to spectral ranges would boast poor robustness for the non-uniformly modified solid samples. More specifically, such would overlap with the amide band. The amide functionality was shown to be present due to transacylation during MOF synthesis, according to HRAM-ESI-MS, in SI Section 3.5. Furthermore, amide solvent DMF from MOF synthesis,[10] was also shown to be retained for MIL-125 NH_2, based on larger than expected N to C ratio, in SI Section 2.2. Consequently, a more characteristic peak was found on the IR spectra, indirectly indicative the extent of target modification formation. Using the predictive tool of cheminfo.org,[11] the peak at 1280 cm^{-1} was identified to be a vibrational mode of the conjugated N-aryl system in the imine products. Considering observed intensities, target product formation was found to be considerable for all attempted modifications with aldehydes. As with ketones, however, target product formation was found to be consistently more modest for all attempts. The spectra are plotted Figure S24 and Figure S25, grouped with regards to MIL-125 NH_2 and UiO-66 NH_2 substrates, respectively.

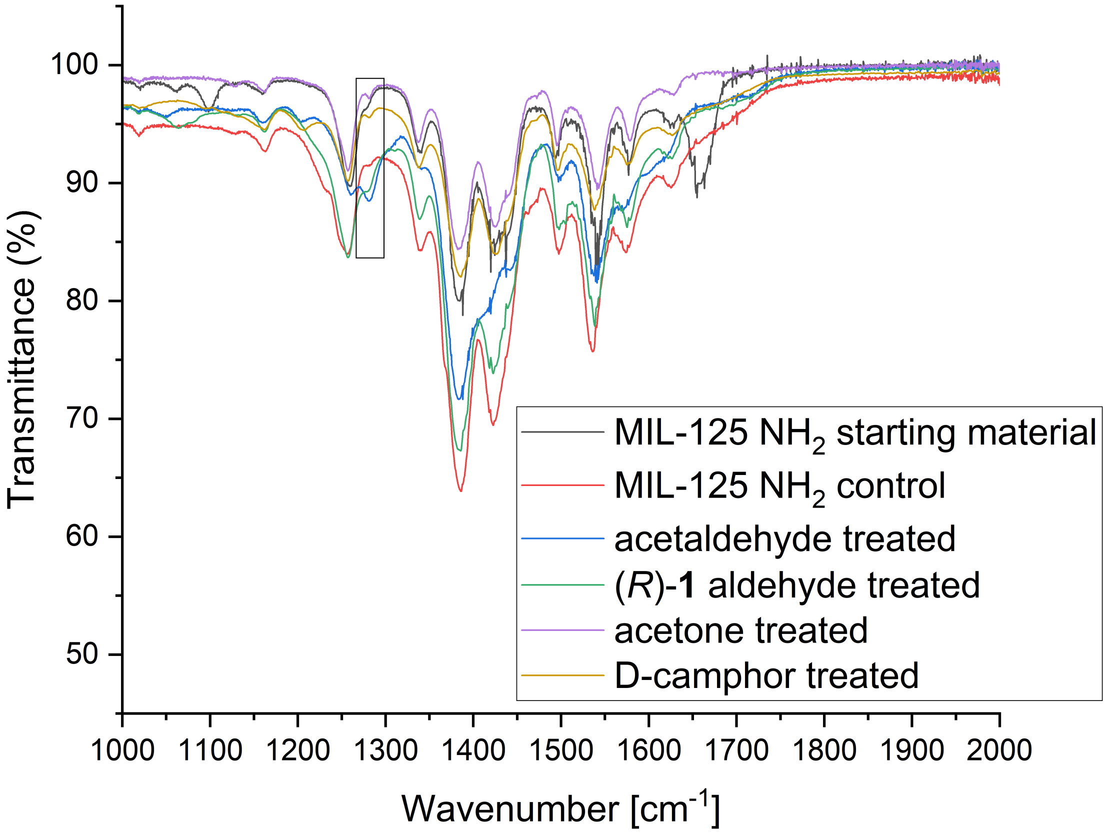

Figure S24: The infrared spectra for the starting materials and for isolates recovered from various indicated treatments, overlaid separately by amino functionalized starting substrate. Spectra pertaining to MIL-125 NH_2 substrate are shown. Spectra accumulated for 400 cm^{-1} to 4000 cm^{-1} range plotted for 1000 cm^{-1} to 2000 cm^{-1} to showcase region of interest. The boxes highlight the peak of interest that was found to be indicative of the target modification formation.

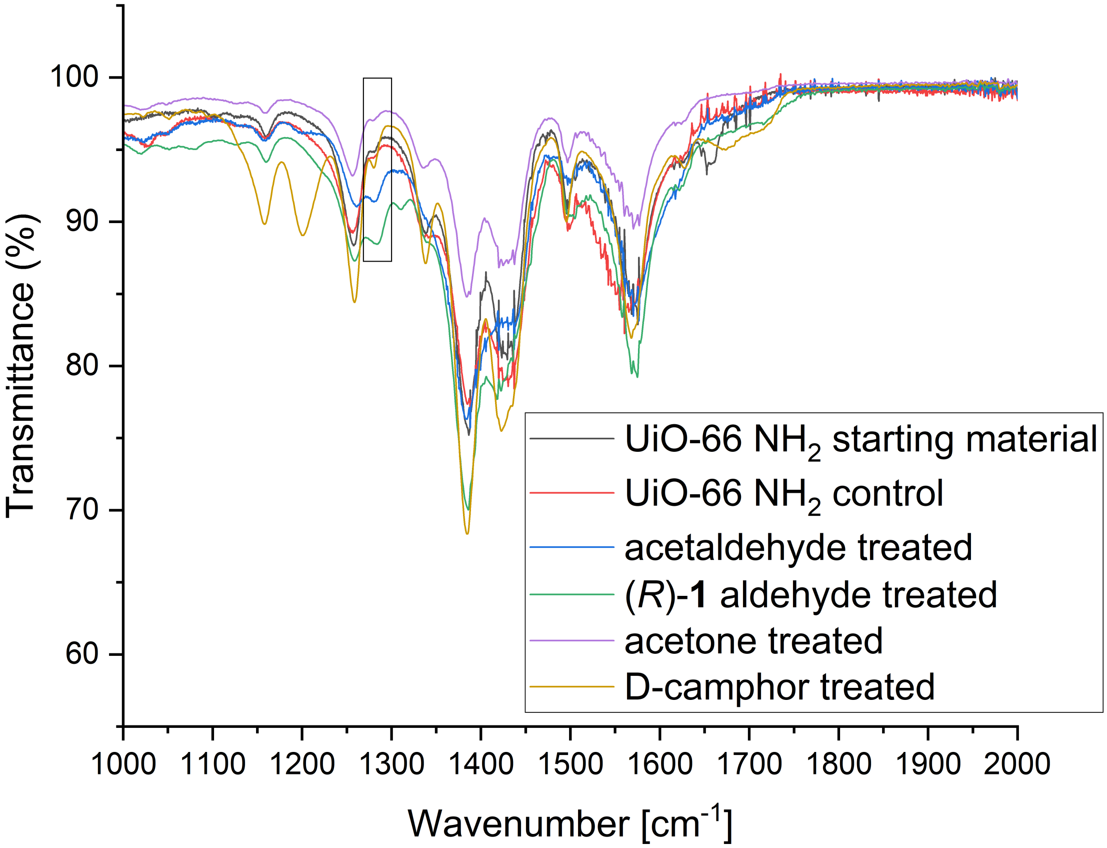

Figure S25: The infrared spectra for the starting materials and for isolates recovered from various indicated treatments, overlaid separately by amino functionalized starting substrate. Spectra pertaining to UiO-66 NH_2 substrate are shown. Spectra accumulated for 400 cm^{-1} to 4000 cm^{-1} range plotted for 1000 cm^{-1} to 2000 cm^{-1} to showcase region of interest. The boxes highlight the peak of interest that was found to be indicative of the target modification formation.

3.4 Nuclear magnetic resonance analyses

As first step of solution state NMR inquiry, precursor materials of the *Model 1* imine formation attempt were analyzed with ^{1}H NMR. Spectra of (*R*)-**1** aldehyde revealed oligomeric nature of the material, in concert with specifications. Such is considered to entail reversible polyoxymethylene chain formation along the aldehyde functionalities. In Figure S27 spectrum was taken in alcoholous-nucleophile free medium to impede deoligomerization. In Figure S28 the solution from Figure S26 was diluted in alcoholous solution to promote deoligomerization. Clearly interpretable shifting of the degree of oligomerization was not found. To rule out concerns regarding starting material degradation, a fresh sample from a new batch was studied in Figure S28 yielding a matching spectral pattern. Spectrum in Figure S30 of the dimethyl 2-aminoterephthalate was unequivocally annotated.

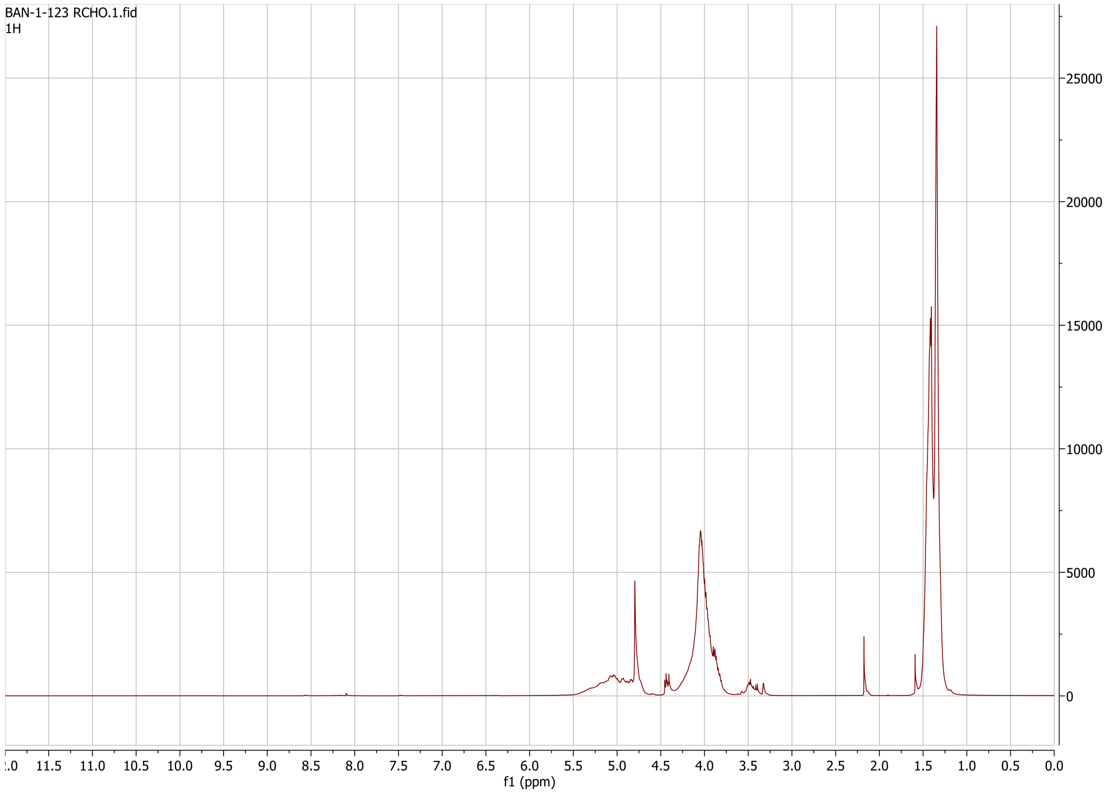

Figure S26: Solution of (*R*)-**1** aldehyde, procured as a 50 m/m% mixture in methylene chloride. ^{1}H NMR (400 MHz, D4-MeOH)

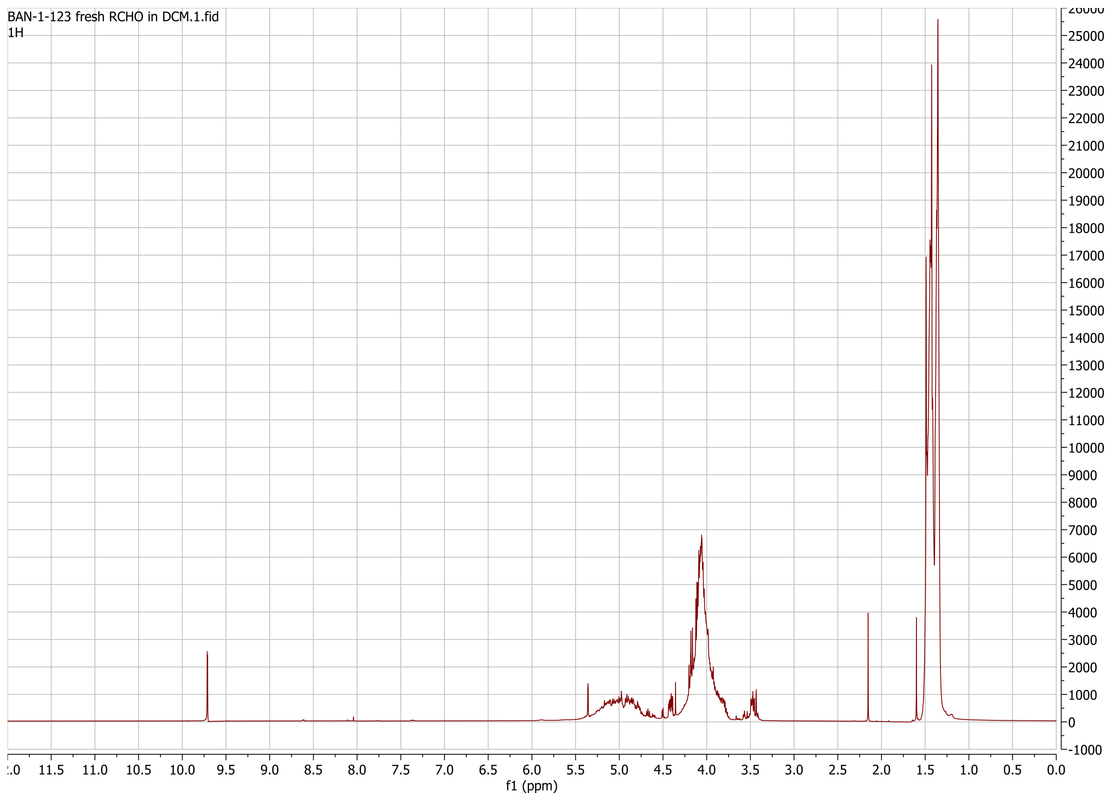

Figure S27: Solution of (R)-**1** aldehyde, procured as a 50 m/m% mixture in methylene chloride. ^{1}H NMR (400 MHz, D2-DCM)

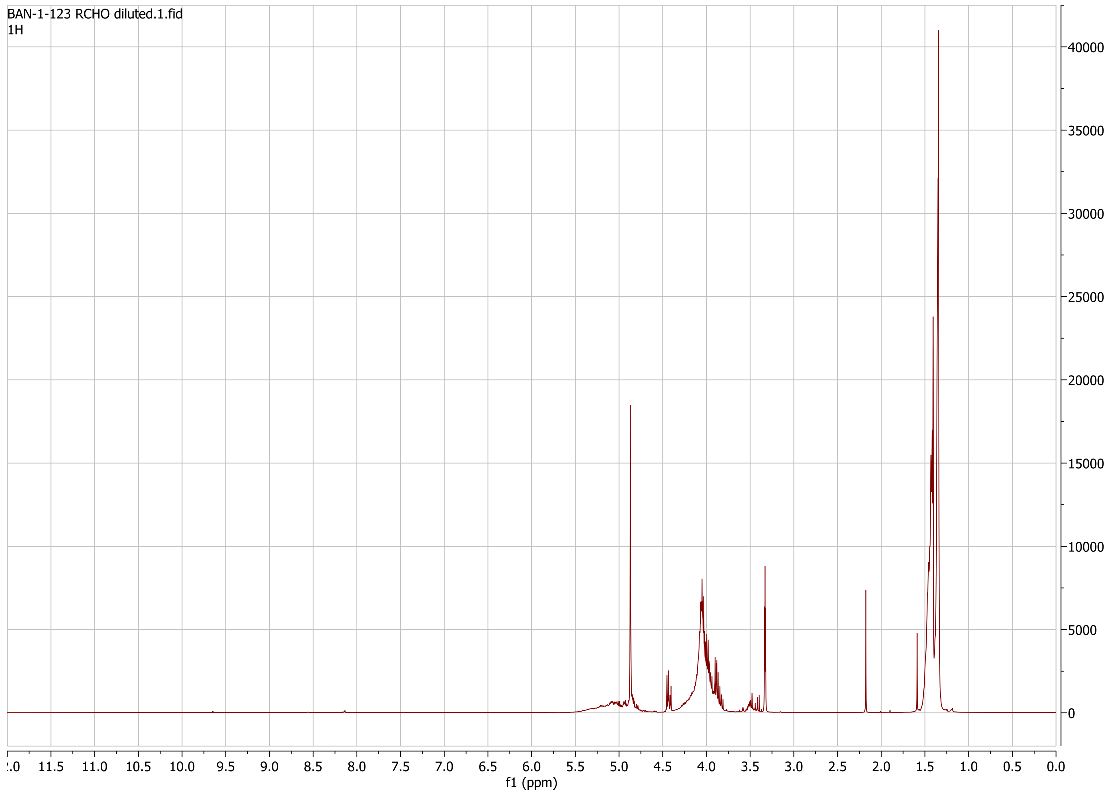

Figure S28: The roughly 10-fold dilution of the Figure S26 solution of (*R*)-**1** aldehyde, procured as a 50 m/m% mixture in methylene chloride. ^{1}H NMR (400 MHz, D4-MeOH)

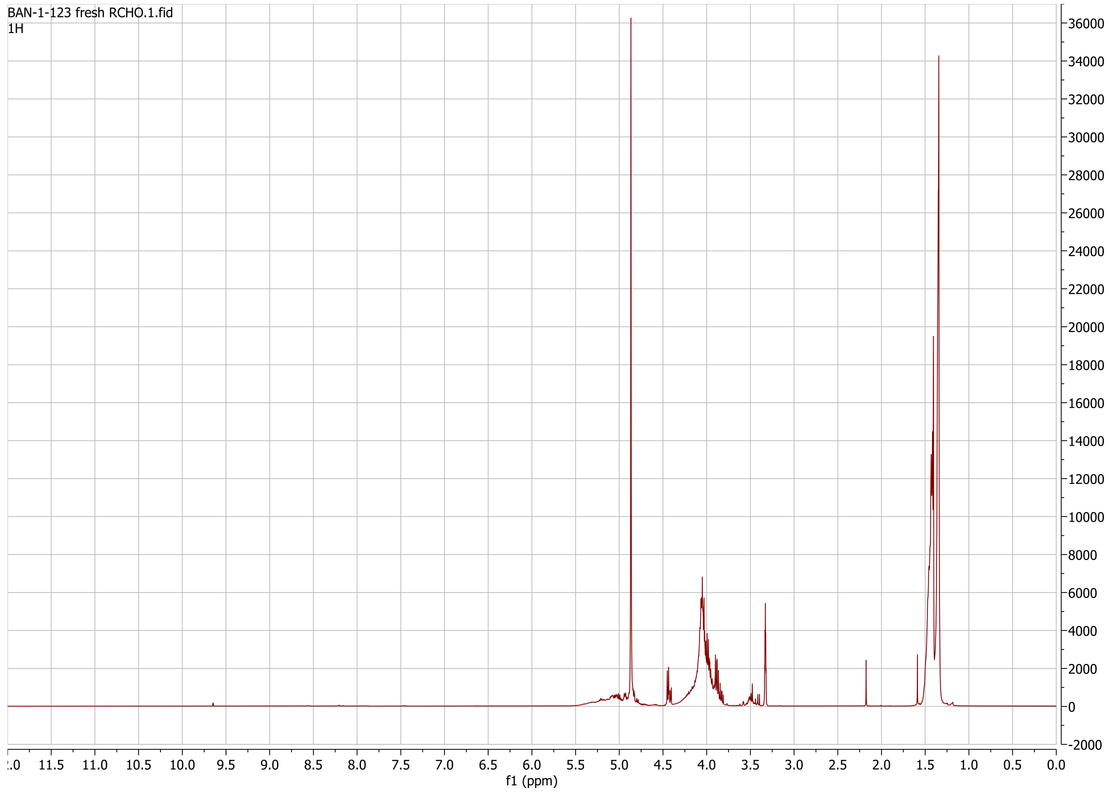

Figure S29: Solution of a new batch of (*R*)-**1** aldehyde, procured as a 50 m/m% mixture in methylene chloride. The solution was prepared promptly after receipt of chemical, fresh upon first opening of the container. ^{1}H NMR (400 MHz, D4-MeOH)

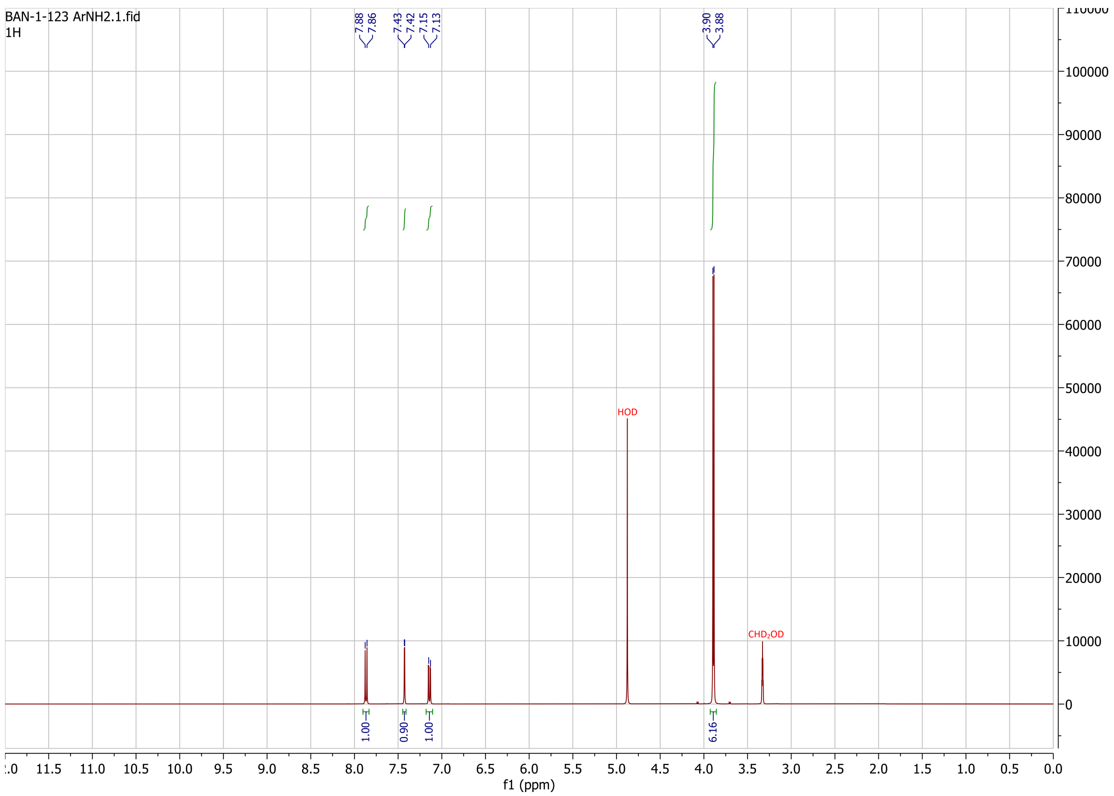

Figure S30: Solution of dimethyl 2-aminoterephthalate. ^{1}H NMR (400 MHz, D4-MeOH) δ 7.87 (d, J = 8.4 Hz, 1H), 7.43 (d, J = 1.7 Hz, 1H), 7.14 (dd, J = 8.4, 1.7 Hz, 1H), 3.90 (s, 3H), 3.88 (s, 3H).

Chemical change in the *Model 1* experiment mixture was thereafter studied by ^{1}H NMR. The spectra in Figure S32, Figure S33, and in Figure S34 were compared. Clear indication of thermally induced chemical reaction, and signs of further chemical change upon refrigerated storage were found. With the aid of ESI-MS and ESI-MS/MS, descriptive coproducts within the *Model 1* product mixture were inferred, as shown in Figure S31. Color coding to denote groups of protons and corresponding chemical shift ranges, introduced in Figure S31, was used to delineate such ranges on NMR spectra from hereon. Given how each building block contributes their signal in largely orthogonal chemical shift ranges, signal plurality in each of these ranges is highly consequential in holistic analysis of building block incorporation.

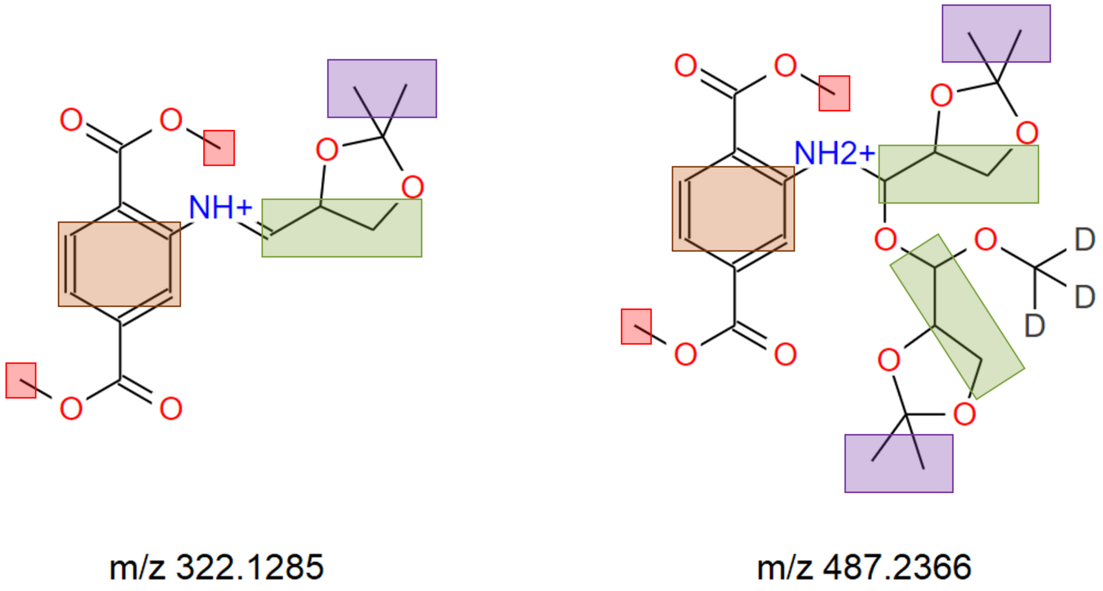

Figure S31: Using positive mode ESI-MS the *Model 1* product mixture was analyzed after prolonged storage. The protonated nominal imine product (left) was found to give a prominent signal. The most intense signal was elucidated to represent a protonated dimeric modification (right), confirmed by ESI-MS/MS. Each building block is expected to contribute protons confined to a characteristic distinguishable chemical shift range in corresponding NMR spectra. Acetonide protons in shielded aliphatic range (purple), glyceraldehyde backbone protons in deshielded aliphatic (green), therein grouped benzoic methyl ester protons (red), and aromatic protons (brown) were marked on showcased MS hits, representative of mixture.

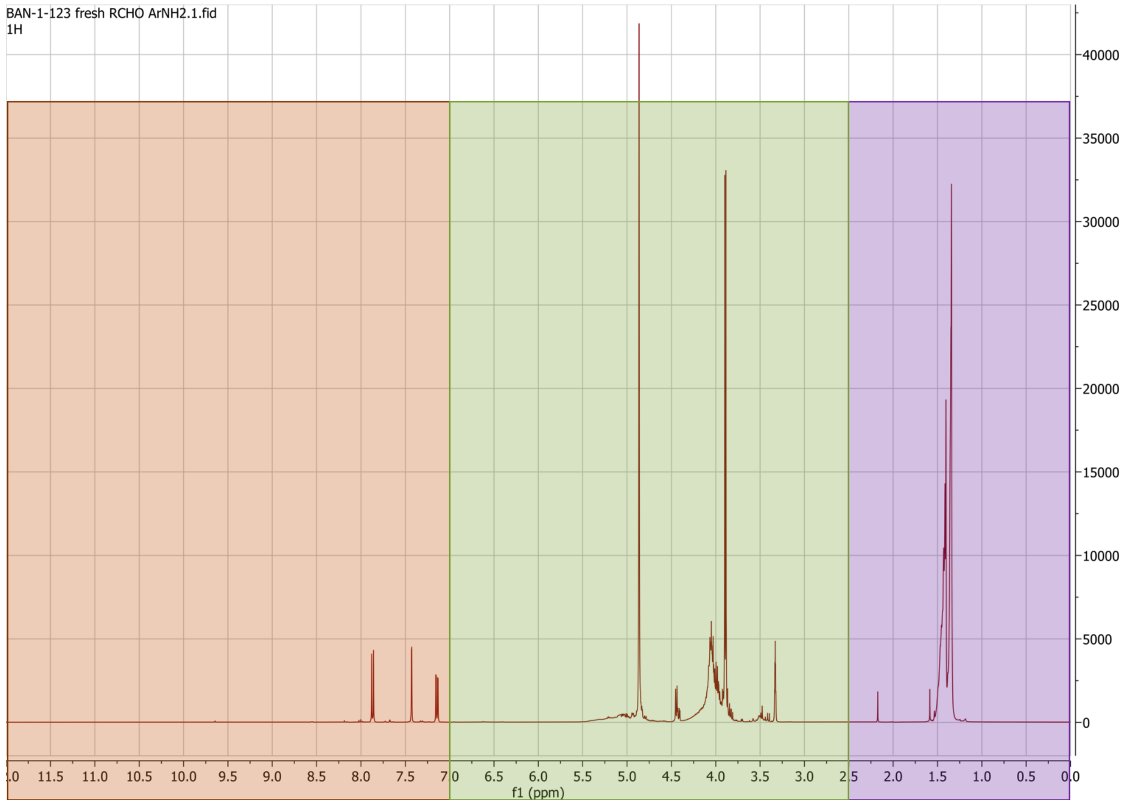

Figure S32: Spectrum of the *Model 1* experiment reaction mixture, taken directly after preparation of solution, before applying temperature program. According to color coding introduced in Figure S31, shielded aliphatic (purple), deshielded aliphatic (green), and aromatic (brown) chemical shift ranges are distinguished. ^{1}H NMR (400 MHz, D4-MeOH)

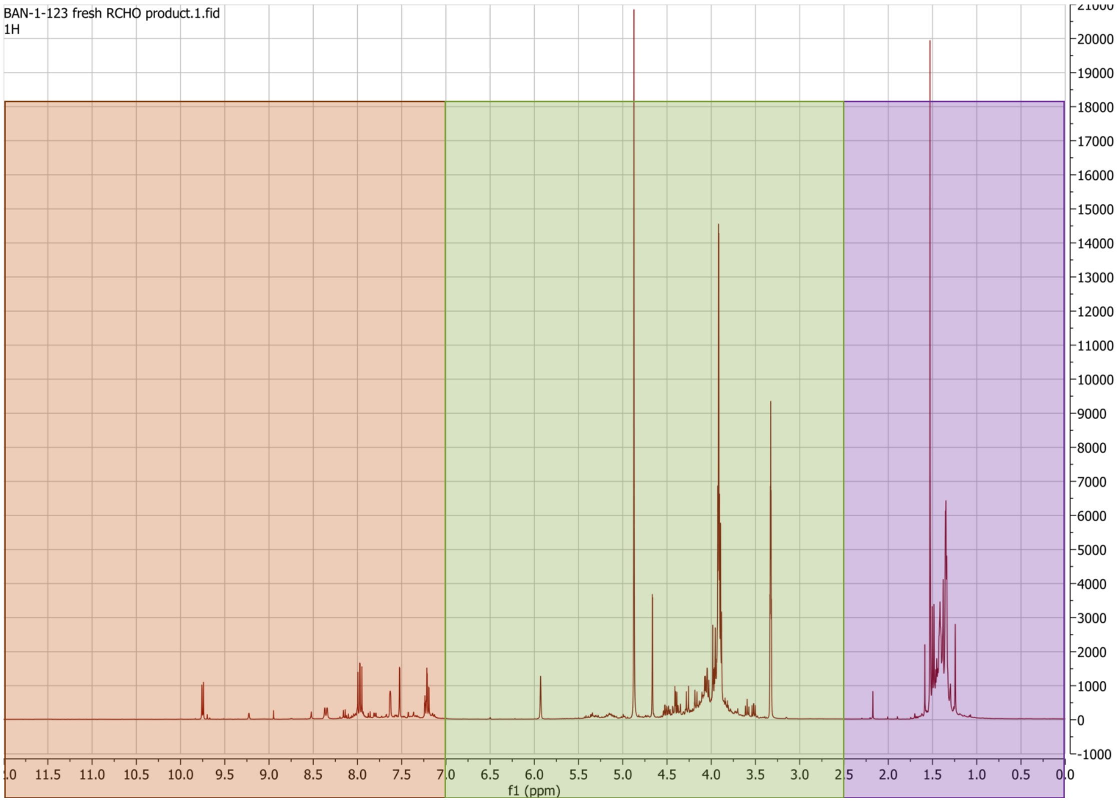

Figure S33: Spectrum of the *Model 1* experiment product, taken after redissolution of the residue obtained after the temperature program. ^{1}H NMR (400 MHz, D4-MeOH)

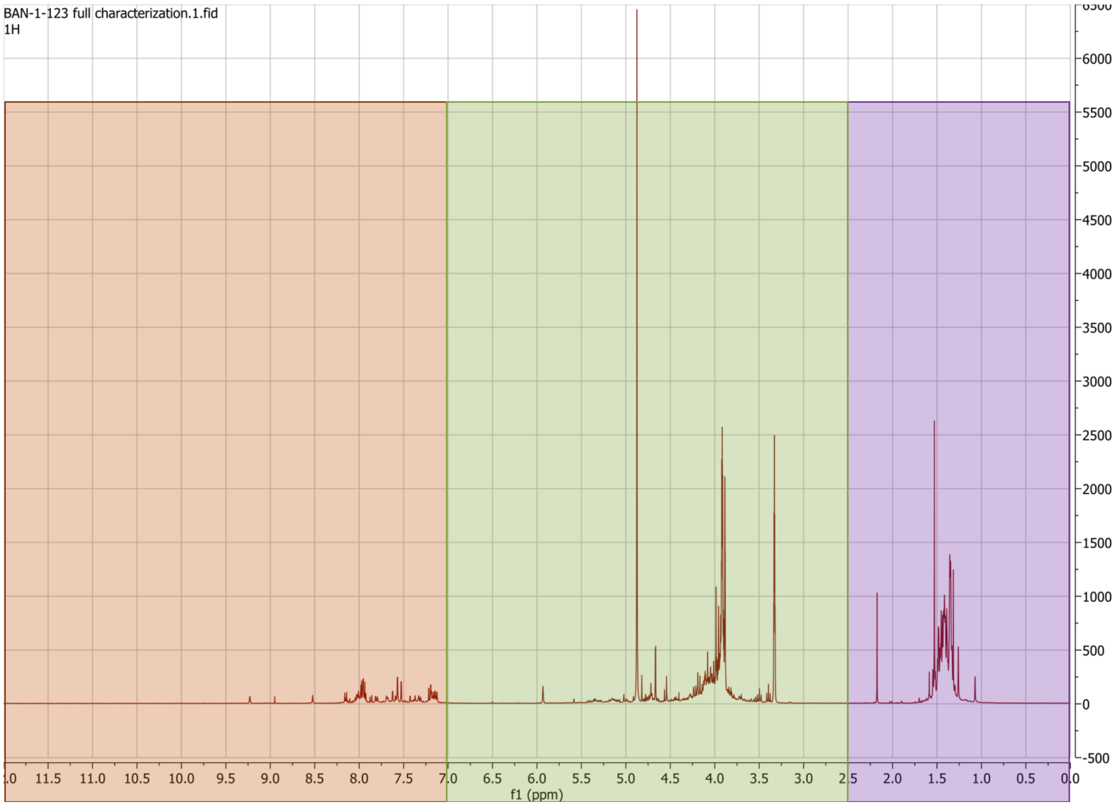

Figure S34: Spectrum of the *Model 1* experiment product, after 12 days of in solution storage at 4 °C. ^{1}H NMR (400 MHz, D4-MeOH)

Beyond the ^{1}H NMR, stored product of the *Model 1* experiment was sequentially further characterized by NMR experiments. Two-dimensional spectra help to better delineate distinct chemical shift ranges defined in Figure S31 earlier. Distinction of benzoic methyl ester (red) signals from glyceraldehyde backbone (green) signals is enabled within the deshielded aliphatic ^{1}H chemical shift range on some of these spectra. Due to the underlying mixture, reportworthy assignments were not attained.

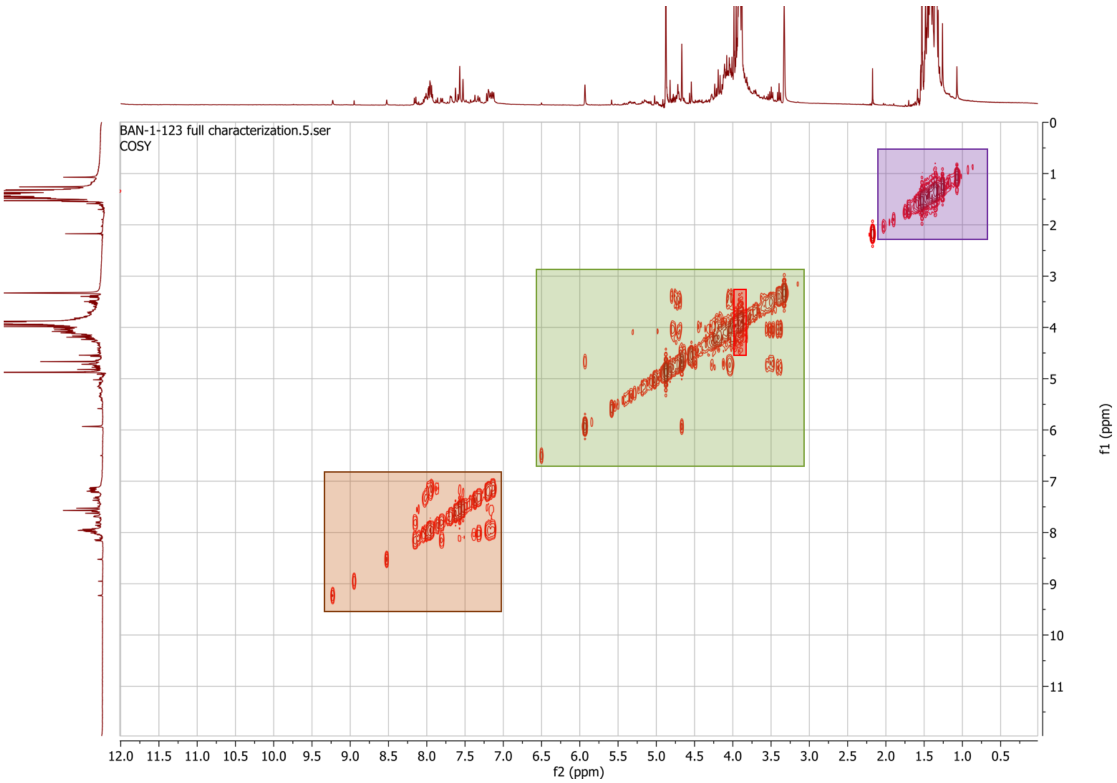

Figure S35: The COSY spectrum of the *Model 1* experiment stored product. According to color coding introduced in Figure S31, shielded aliphatic (purple), deshielded aliphatic (green), therein benzoic methyl ester (red), and aromatic (brown) chemical shift ranges are distinguished.

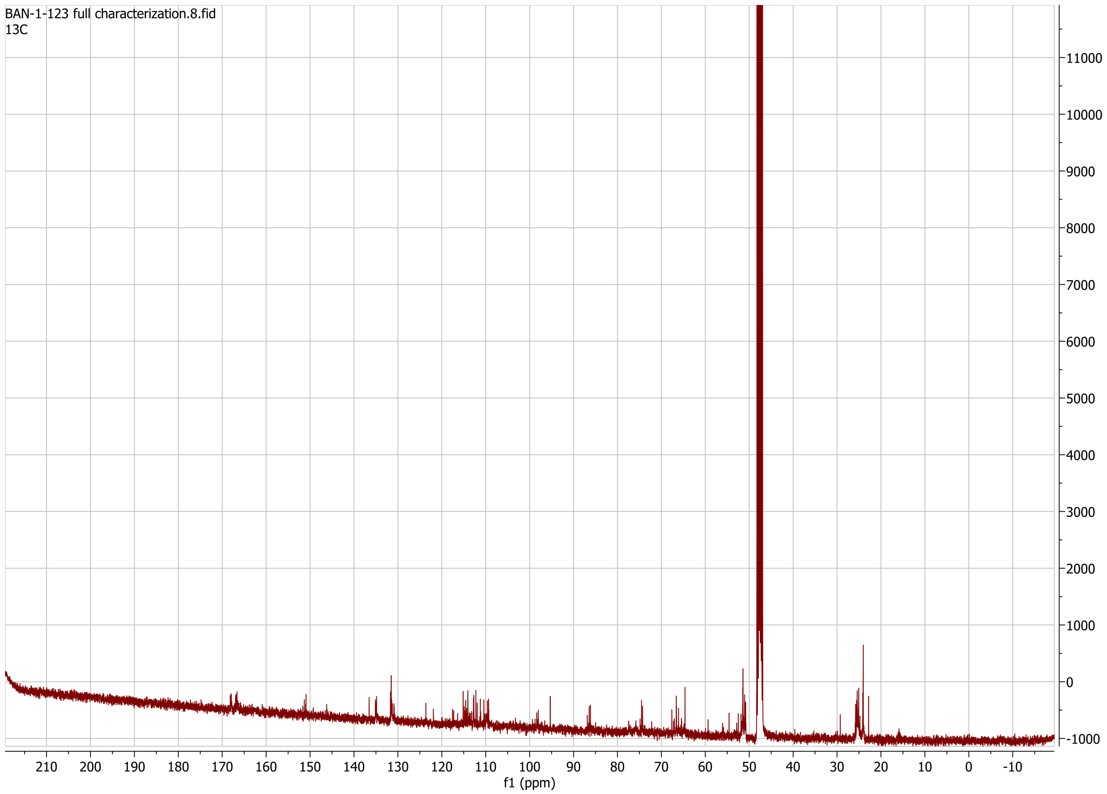

Figure S36: Spectrum of the *Model 1* experiment stored product. ^{13}C NMR (101 MHz, D4-MeOH)

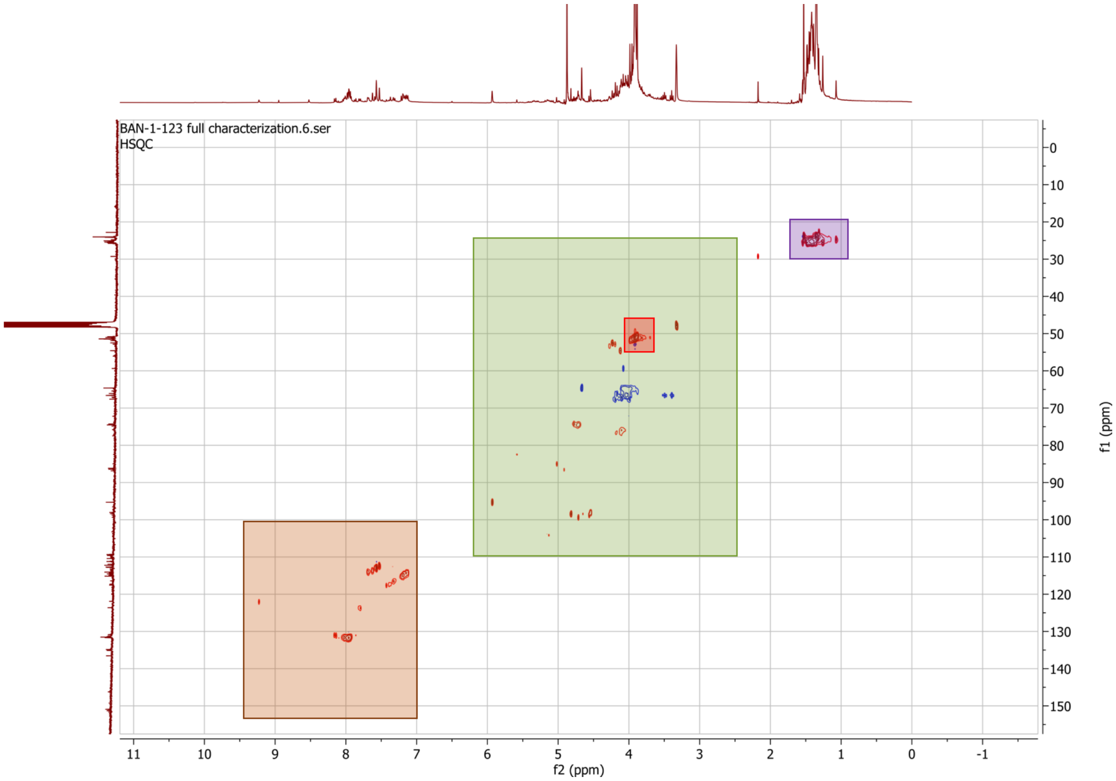

Figure S37: The HSQC spectrum of the *Model 1* experiment stored product. According to color coding introduced in Figure S31, shielded aliphatic (purple), deshielded aliphatic (green), therein benzoic methyl ester (red), and aromatic (brown) chemical shift ranges are distinguished.

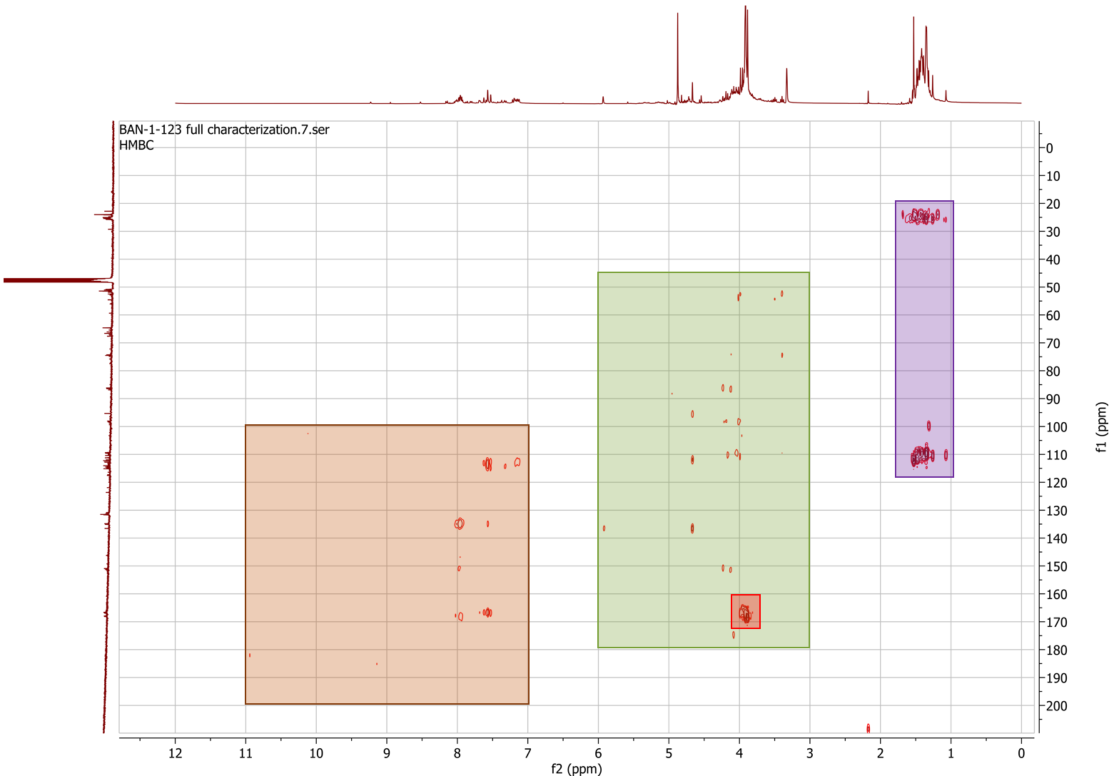

Figure S38: The HMBC spectrum of the *Model 1* experiment stored product. According to color coding introduced in Figure S31, shielded aliphatic (purple), deshielded aliphatic (green), therein benzoic methyl ester (red), and aromatic (brown) chemical shift ranges are distinguished.

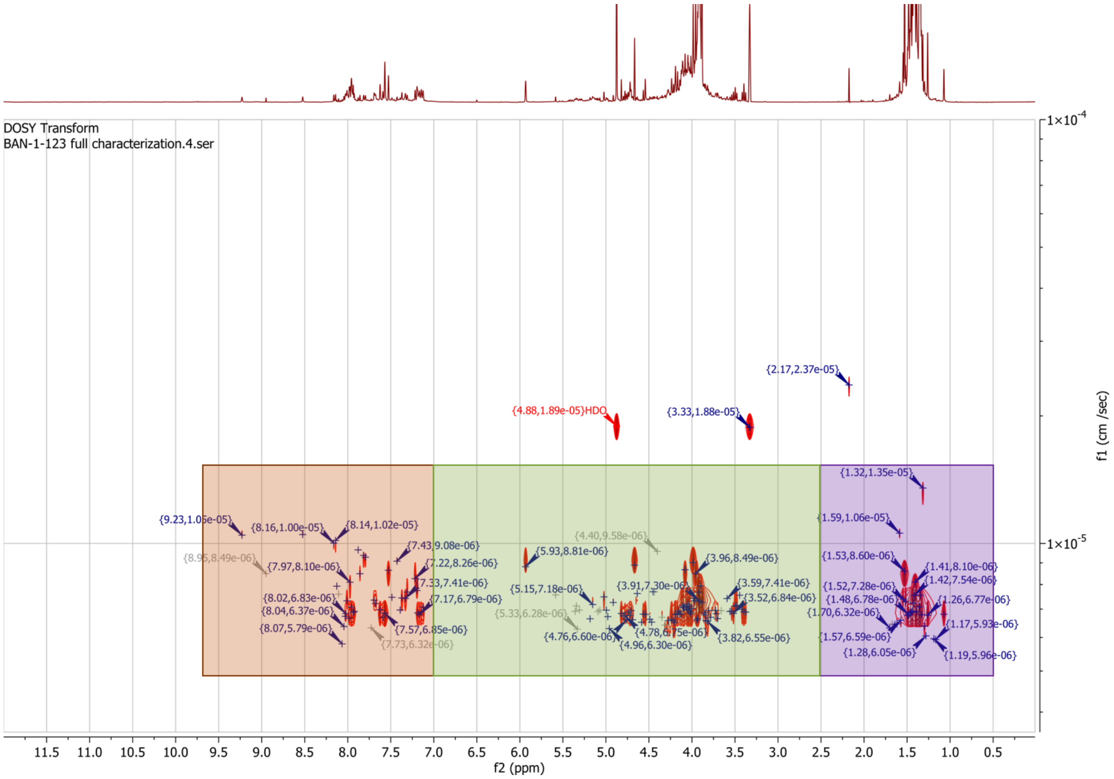

Figure S39: The DOSY spectrum of the *Model 1* experiment stored product. According to color coding introduced in Figure S31, shielded aliphatic (purple), deshielded aliphatic (green), and aromatic (brown) chemical shift ranges are distinguished.

3.5 High resolution accurate mass MS analyses

When analyzing the HRAM-ESI-MS data in positive mode, cation formation via electron loss or protonation, or coordination of Na^+, K^+, and Cs^+ cations were considered for all presented experiments. In negative mode, only deprotonation was considered, uniformly in the study.

Table S12: The 10 most intense MS signals in positive mode of the *Model 2* ethanolous solution, in descending order of intensity. Monoisotopic masses and corresponding probed formulae are shown, according to laxed chemical constraints. Composition is represented by string of integers if successfully discerned. The * marks a determined formula that may not be written as a combination of the former.

			M_mi [Da]	181.0375	90.0317	58.0419	46.0419	18.0106	2.0157	43.9898	41.0265	27.9949
m/z [ ]	Intensity (%)	Charge [ ]	Ionization	$C_8H_7NO_4$	$C_3H_6O_3$	C_3H_6O	C_2H_6O	H_2O	H_2	CO_2	C_2H_3N	CO
294.0973	103.84	1	H^+	1	1	1	0	-2	0	0	0	0
199.0941	42.10	1	Na^+	0	1	1	1	-1	0	0	0	0
118.1226	23.19	1	H^+	*	*	*	*	*	*	*	*	*
316.0791	20.77	1	Na^+	1	1	1	0	-2	0	0	0	0
236.0554	20.44	1	H^+	1	1	0	0	-2	0	0	0	0
478.2071	20.06	1										
253.1411	19.87	1										
164.0342	17.78	1	H^+	1	0	0	0	-1	0	0	0	0
392.1704	16.33	1										
295.1005	16.17	1										

Table S13: The 10 most intense MS signals in negative mode of the *Model 2* ethanolous solution, in descending order of intensity. Monoisotopic masses and corresponding probed formulae are shown, according to laxed chemical constraints. Composition is represented by string of integers if successfully discerned.

		M_mi [Da]	181.0375	90.0317	58.0419	46.0419	18.0106	2.0157	43.9898	41.0265	27.9949	1.0079
m/z [ ]	Intensity (%)	Charge [ ]	$C_8H_7NO_4$	$C_3H_6O_3$	C_3H_6O	C_2H_6O	H_2O	H_2	CO_2	C_2H_3N	CO	H^+
180.0303	100.00	-1	1	0	0	0	0	0	0	0	0	-1
324.1090	44.19	-1	1	1	0	2	-2	-1	0	0	0	-1
292.0828	16.48	-1	1	1	1	0	-2	0	0	0	0	-1
364.1398	13.33	-1										
585.1726	13.17	-1										
450.1766	12.46	-1	1	3	0	0	0	0	0	0	0	-1
136.0405	10.32	-1	1	0	0	0	0	0	-1	0	0	-1
181.0335	8.90	-1										
350.1241	7.85	-1										
325.1123	7.29	-1										

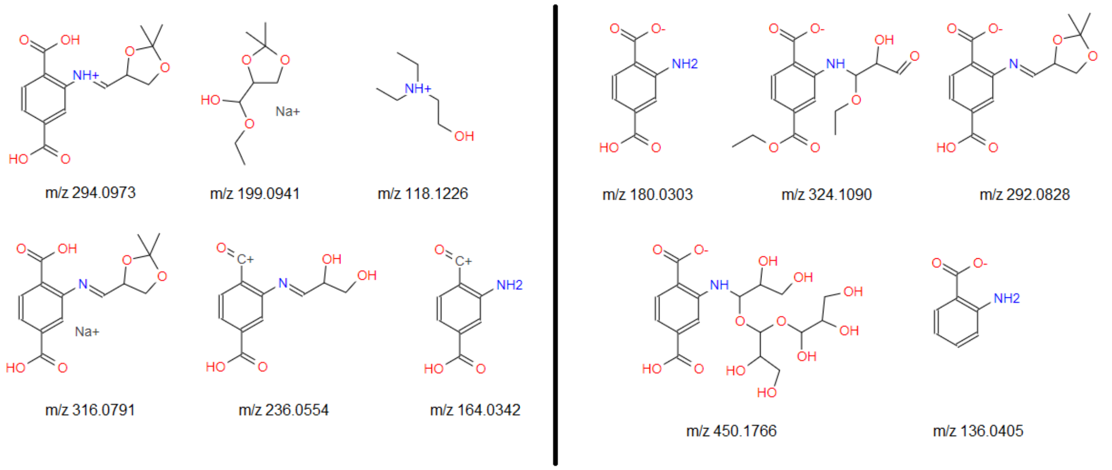

Figure S40: The structure of the ions for the 10 most intense respective MS signals of the *Model 2* ethanolous solution, where formulae were discerned. Shown separately for positive mode (left), and negative mode (right). Respective order of structures corresponds to signal intensity. Representation reflects chemical bias, isomerism is permitted as applicable.

Table S14: The 10 most intense MS signals in positive mode of the *Digestion method 1* solubilized *Model 2* residue, in descending order of intensity. Monoisotopic masses and corresponding probed formulae are shown, according to laxed chemical constraints. Composition is represented by string of integers if successfully discerned.

			M_mi [Da]	181.0375	90.0317	58.0419	46.0419	18.0106	2.0157	43.9898	41.0265	27.9949
m/z [ ]	Intensity (%)	Charge [ ]	Ionization	$C_8H_7NO_4$	$C_3H_6O_3$	C_3H_6O	C_2H_6O	H_2O	H_2	CO_2	C_2H_3N	CO
371.0465	100.76	1										
327.0202	47.58	1										
415.0727	24.71	1	Cs^+	0	2	0	3	-2	0	0	0	0
282.8130	24.44	1										
583.2604	17.66	1										
627.2865	16.84	1										
539.2343	15.83	1										
671.3128	14.50	1										
715.3388	11.47	1										
372.0497	11.11	1										

Table S15: The 10 most intense MS signals in negative mode of the *Digestion method 1* solubilized *Model 2* residue, in descending order of intensity. Monoisotopic masses and corresponding probed formulae are shown, according to laxed chemical constraints. Composition is represented by string of integers if successfully discerned.

		M_mi [Da]	181.0375	90.0317	58.0419	46.0419	18.0106	2.0157	43.9898	41.0265	27.9949	1.0079
m/z [ ]	Intensity (%)	Charge [ ]	$C_8H_7NO_4$	$C_3H_6O_3$	C_3H_6O	C_2H_6O	H_2O	H_2	CO_2	C_2H_3N	CO	H^+
338.1248	100.07	1	1	1	1	1	-2	0	0	0	0	-1
364.1395	93.90	1										
422.1451	81.22	1										
450.1764	70.05	1										
255.2330	67.11	1										
224.5849	55.52	2										
350.1238	44.19	1										
292.0828	38.78	1	1	1	1	0	-2	0	0	0	0	-1
325.1844	34.49	1										
311.1688	31.15	1										

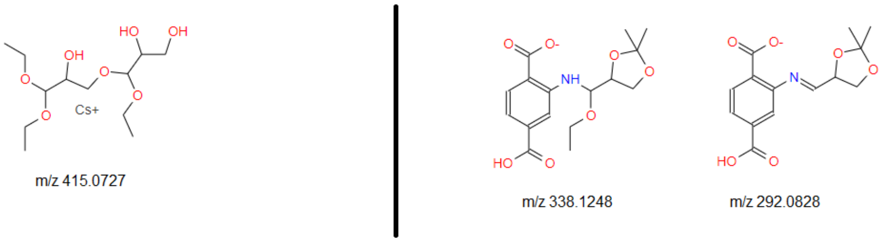

Figure S41: The structure of the ions for the 10 most intense respective MS signals of the *Digestion method 1* solubilized *Model 2* residue, where formulae were discerned. Shown separately for positive mode (left), and negative mode (right). Respective order of structures corresponds to signal intensity. Representation reflects chemical bias, isomerism is permitted as applicable.

Table S16: The 10 most intense MS signals in positive mode of the *Digestion method 1* solubilized UiO-66 NH_2 substrate that was (R)-**1** aldehyde treated, in descending order of intensity. Monoisotopic masses and corresponding probed formulae are shown, according to laxed chemical constraints. Composition is represented by string of integers if successfully discerned.

			M_mi [Da]	181.0375	90.0317	58.0419	46.0419	18.0106	2.0157	43.9898	41.0265	27.9949
m/z [ ]	Intensity (%)	Charge [ ]	Ionization	$C_8H_7NO_4$	$C_3H_6O_3$	C_3H_6O	C_2H_6O	H_2O	H_2	CO_2	C_2H_3N	CO
415.0727	100.43	1	Cs^+	0	2	0	3	-2	0	0	0	0
297.0097	87.84	1	Cs^+	0	1	0	2	-1	0	0	0	0
647.3103	55.45	1										
397.0622	44.78	1										
371.0464	35.65	1										
324.8236	32.96	1										
282.8130	29.15	5										
327.0203	27.11	1										
354.8341	26.89	1										
173.9314	26.51	1	Cs^+	0	0	0	0	0	0	0	1	0

Table S17: The 10 most intense MS signals in negative mode of the *Digestion method 1* solubilized UiO-66 NH_2 substrate that was (R)-**1** aldehyde treated, in descending order of intensity. Monoisotopic masses and corresponding probed formulae are shown, according to laxed chemical constraints. Composition is represented by string of integers if successfully discerned.

		M_mi [Da]	181.0375	90.0317	58.0419	46.0419	18.0106	2.0157	43.9898	41.0265	27.9949	1.0079
m/z [ ]	Intensity (%)	Charge [ ]	$C_8H_7NO_4$	$C_3H_6O_3$	C_3H_6O	C_2H_6O	H_2O	H_2	CO_2	C_2H_3N	CO	H^+
180.0302	102.99	1	1	0	0	0	0	0	0	0	0	-1
208.0251	87.46	1	1	0	0	0	0	0	0	0	1	-1
293.1759	29.98	1										
255.2330	28.67	1										
296.1140	13.96	1										
208.0614	13.76	1	1	0	0	1	-1	0	0	0	0	-1
222.0408	12.42	1										
171.1391	11.99	1										
283.2643	11.92	1										
325.1843	11.88	1										

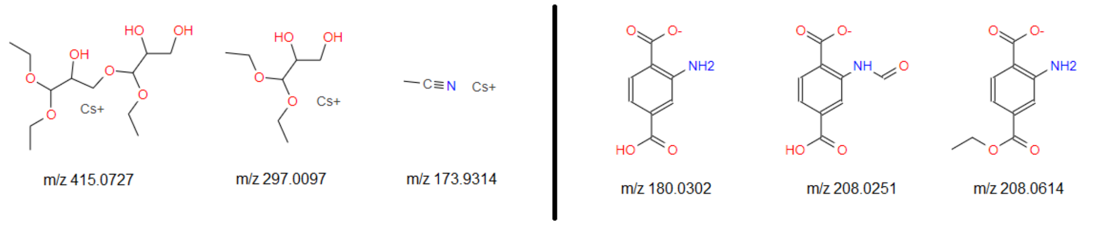

Figure S42: The structure of the ions for the 10 most intense respective MS signals of the *Digestion method 1* solubilized UiO-66 NH_2 substrate that was (R)-**1** aldehyde treated, where formulae were discerned. Shown separately for positive mode (left), and negative mode (right). Respective order of structures corresponds to signal intensity. Representation reflects chemical bias, isomerism is permitted as applicable.

The statistical set was constructed from the building blocks, listed in Table S18, after weighing relevance to the experimental context. Using the same letter code notation, pertaining properties are detailed in Figure S43, including bounds for respective integers. Such bounds were delimited by maximizing the incorporation of the modifying agent to two units, and then interpreting underlying chemistry to determine them for ethanol and water.

Table S18: The letter code denoted list of the systematic names of building block molecules.

Letter	Systematic name
a	2-aminobenzene-1,4-dicarboxylic acid
b	2,3-dihydroxypropanal
c	2-propanone
d	water
e	ethanol

Letter	Minimum	Maximum	Structure	Formula	M_{mi} [Da]
**a**	1	1	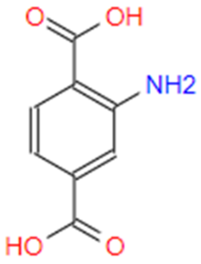	$C_8H_7NO_4$	181.0375
**b**	1	2	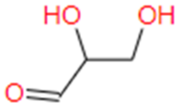	$C_3H_6O_3$	90.0317
**c**	0	2	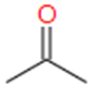	C_3H_6O	58.0419
**d**	-3	0	OH2	H_2O	18.0106
**e**	0	1	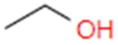	C_2H_6O	46.0419

Figure S43: The properties of the building blocks of the statistical set. The established letter codes, and the limits for the thereby represented integers. Negative values signify condensation. Structure, formula, and monoisotopic mass are shown in successive columns.

Only permitting the incorporation of acetone as an acetonide adduct, Equation (11) arises, as number of the former is thereby limited to the number of aldehyde units. Letters signify integers denoted by them. Considerations for degree of condensation further constrain the test set to either of the subcases listed in Table S19.

$$b \geq c \tag{11}$$

Table S19: Chemically interpretable subcases of the test set, following the introduction further constraints. The b+c+d+e sum of integers signifies the degree of condensation. Subcases are further categorized by the monomeric or dimeric nature of the aldehyde modification, and by the presence of ethanol, each indicated by the applicable letter code. Respective end groups, and polymer backbone linkage chemistry for dimers are indicated for the subcases. The number of resulting molecules in each subset is shown in the rightmost column.

b+c+d+e	b	e	End group	Linkage	Targets
1	1	0	hemiaminal	-	2
0	1	0	imine	-	2
1	1	1	ether	-	2
1	2	0	hemiaminal	acetal	2
0	2	0	imine	acetal	2
1	2	1	ether	acetal	2
2	2	0	hemiacetal	oxomethylene	3
2	2	1	ether	oxomethylene	3

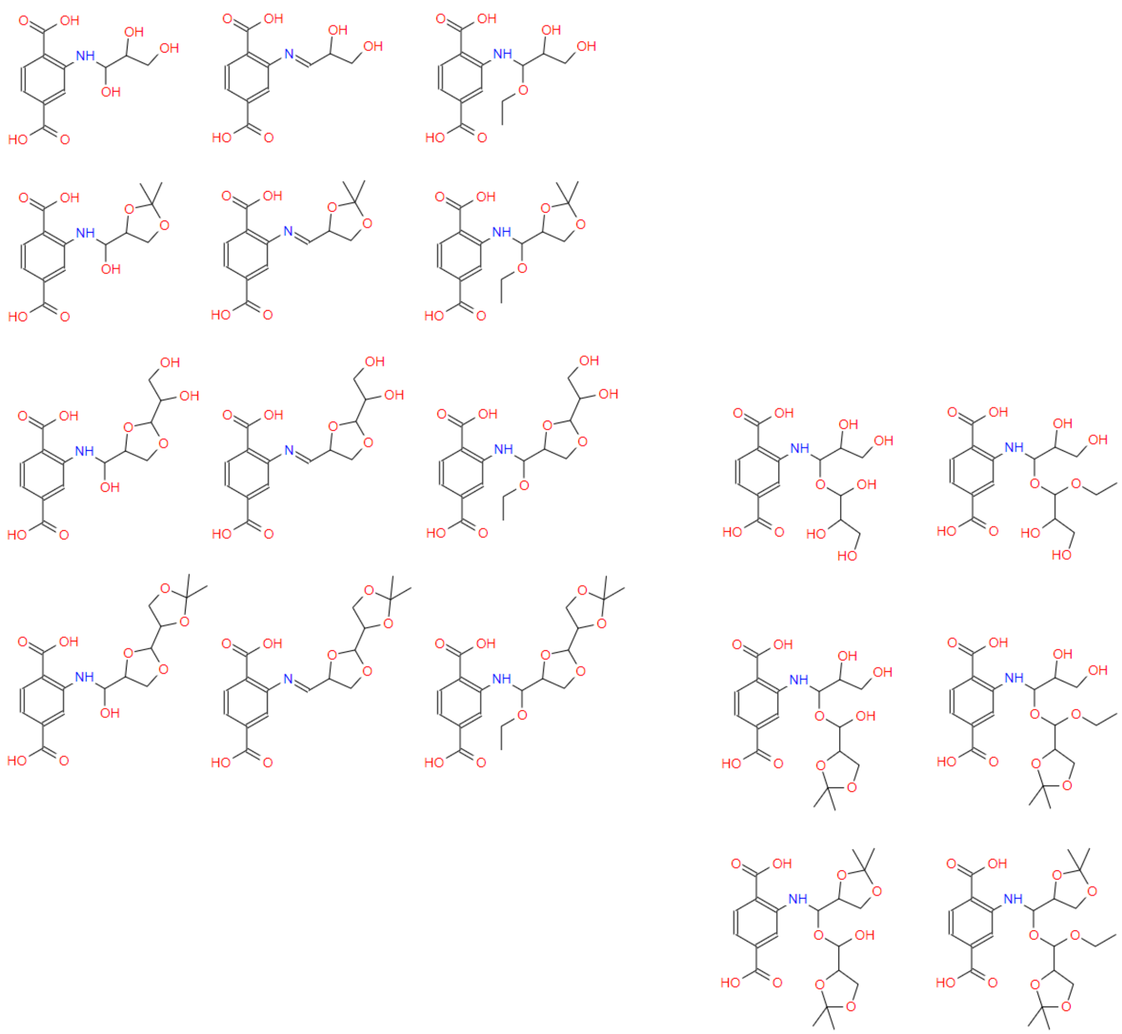

Figure S44: Structure of the 18 resulting molecules of the statistical test set, arranged according to chemical patterns. Admissible isomerism is limited to the position of the single acetonide adduct, if two pertaining vicinal diol sites are available. Thereby obtained constitutional isomers are not plotted separately.

Table S20: Found MS signals corresponding to the test set in positive mode of the *Model 2* ethanolous solution, in ascending order of m/z ratio. Monoisotopic masses and corresponding probed formulae are shown for test set building blocks. Composition is represented by string of integers.

		M_mi [Da]	181.0375	90.0317	58.0419	46.0419	18.0106
m/z [ ]	Charge [ ]	Ionization	$C_8H_7NO_4$	$C_3H_6O_3$	C_3H_6O	C_2H_6O	H_2O
254.0659	1	H^+	1	1	0	0	-1
276.0479	1	Na^+	1	1	0	0	-1
293.0894	1	+	1	1	1	0	-2
294.0972	1	H^+	1	1	1	0	-2
299.1000	1	+	1	1	0	1	-1
300.1078	1	H^+	1	1	0	1	-1
312.1078	1	H^+	1	1	1	0	-1
316.0792	1	Na^+	1	1	1	0	-2
322.0897	1	Na^+	1	1	0	1	-1
326.0870	1	H^+	1	2	0	0	-2
332.0531	1	K^+	1	1	1	0	-2
334.0897	1	Na^+	1	1	1	0	-1
340.1391	1	H^+	1	1	1	1	-2
344.0976	1	H^+	1	2	0	0	-1
362.1210	1	Na^+	1	1	1	1	-2
366.0796	1	Na^+	1	2	0	0	-1
366.1183	1	H^+	1	2	1	0	-3
372.1289	1	H^+	1	2	0	1	-2
378.0950	1	K^+	1	1	1	1	-2
384.1289	1	H^+	1	2	1	0	-2
388.1003	1	Na^+	1	2	1	0	-3
390.1359	1	H^+	1	2	0	1	-1
402.1395	1	+	1	2	1	0	-1
406.1109	1	Na^+	1	2	1	0	-2
412.1602	1	H^+	1	2	1	1	-3
422.0848	1	K^+	1	2	1	0	-2
423.1524	1	+	1	2	2	0	-3
429.1629	1	+	1	2	1	1	-2
434.1422	1	Na^+	1	2	1	1	-3
440.0954	1	K^+	1	2	1	0	-1
442.1708	1	H^+	1	2	2	0	-2
446.1422	1	Na^+	1	2	2	0	-3
452.1527	1	Na^+	1	2	1	1	-2
464.1527	1	Na^+	1	2	2	0	-2
468.1267	1	K^+	1	2	1	1	-2
469.1942	1	+	1	2	2	1	-3
470.2021	1	H^+	1	2	2	1	-3
492.1840	1	Na^+	1	2	2	1	-3
508.1580	1	K^+	1	2	2	1	-3

Table S21: Found MS signals corresponding to the test set in negative mode of the *Model 2* ethanolous solution, in ascending order of m/z ratio. Monoisotopic masses and corresponding probed formulae are shown for test set building blocks. Composition is represented by string of integers.

	M_mi [Da]	181.0375	90.0317	58.0419	46.0419	18.0106	1.0078
m/z [ ]	Charge [ ]	$C_8H_7NO_4$	$C_3H_6O_3$	C_3H_6O	C_2H_6O	H_2O	H^+
190.5535	-2	1	2	1	0	-2	-2
204.5692	-2	1	2	1	1	-3	-2
252.0514	-1	1	1	0	0	-1	-1
292.0827	-1	1	1	1	0	-2	-1
298.0932	-1	1	1	0	1	-1	-1
310.0932	-1	1	1	1	0	-1	-1
338.1245	-1	1	1	1	1	-2	-1
364.1038	-1	1	2	1	0	-3	-1
370.1144	-1	1	2	0	1	-2	-1
382.1144	-1	1	2	1	0	-2	-1
388.1249	-1	1	2	0	1	-1	-1
400.1249	-1	1	2	1	0	-1	-1
410.1457	-1	1	2	1	1	-3	-1
440.1562	-1	1	2	2	0	-2	-1
468.1875	-1	1	2	2	1	-3	-1

Table S22: Found MS signals corresponding to the test set in positive mode of the *Digestion method 1* solubilized *Model 2* residue, in ascending order of m/z ratio. Monoisotopic masses and corresponding probed formulae are shown for test set building blocks. Composition is represented by string of integers.

		M_mi [Da]	181.0375	90.0317	58.0419	46.0419	18.0106
m/z [ ]	Charge [ ]	Ionization	$C_8H_7NO_4$	$C_3H_6O_3$	C_3H_6O	C_2H_6O	H_2O
272.0765	1	H^+	1	1	0	0	0
292.0218	1	K^+	1	1	0	0	-1
338.0637	1	K^+	1	1	0	1	-1
350.0637	1	K^+	1	1	1	0	-1
362.1082	1	H^+	1	2	0	0	0
364.0429	1	K^+	1	2	0	0	-2
378.0950	1	K^+	1	1	1	1	-2
382.0535	1	K^+	1	2	0	0	-1
384.0901	1	Na^+	1	2	0	0	0
389.1316	1	$+$	1	2	0	1	-1
390.1395	1	H^+	1	2	0	1	-1
400.0641	1	K^+	1	2	0	0	0
404.0742	1	K^+	1	2	1	0	-3
406.1109	1	Na^+	1	2	1	0	-2
410.0848	1	K^+	1	2	0	1	-2
411.1524	1	$+$	1	2	1	1	-3
412.1602	1	H^+	1	2	1	1	-3
422.0848	1	K^+	1	2	1	0	-2
425.9948	1	Cs^+	1	1	1	0	-2
428.0954	1	K^+	1	2	0	1	-1
430.1708	1	H^+	1	2	1	1	-2
434.1422	1	Na^+	1	2	1	1	-3
440.0954	1	K^+	1	2	1	0	-1
450.1161	1	K^+	1	2	1	1	-3
464.1527	1	Na^+	1	2	2	0	-2
468.1267	1	K^+	1	2	1	1	-2
472.0367	1	Cs^+	1	1	1	1	-2
480.1267	1	K^+	1	2	2	0	-2
492.1840	1	Na^+	1	2	2	1	-3
516.0265	1	Cs^+	1	2	1	0	-2
556.0578	1	Cs^+	1	2	2	0	-3

Table S23: Found MS signals corresponding to the test set in negative mode of the *Digestion method 1* solubilized *Model 2* residue, in ascending order of m/z ratio. Monoisotopic masses and corresponding probed formulae are shown for test set building blocks. Composition is represented by string of integers.

	M_mi [Da]	181.0375	90.0317	58.0419	46.0419	18.0106	1.0078
m/z [ ]	Charge [ ]	$C_8H_7NO_4$	$C_3H_6O_3$	C_3H_6O	C_2H_6O	H_2O	H^+
190.5535	-2	1	2	1	0	-2	-2
204.5692	-2	1	2	1	1	-3	-2
252.0514	-1	1	1	0	0	-1	-1
270.0619	-1	1	1	0	0	0	-1
292.0827	-1	1	1	1	0	-2	-1
310.0932	-1	1	1	1	0	-1	-1
324.0725	-1	1	2	0	0	-2	-1
338.1245	-1	1	1	1	1	-2	-1
342.0831	-1	1	2	0	0	-1	-1
364.1038	-1	1	2	1	0	-3	-1
370.1144	-1	1	2	0	1	-2	-1
382.1144	-1	1	2	1	0	-2	-1
388.1249	-1	1	2	0	1	-1	-1
400.1249	-1	1	2	1	0	-1	-1
410.1457	-1	1	2	1	1	-3	-1
428.1562	-1	1	2	1	1	-2	-1
440.1562	-1	1	2	2	0	-2	-1
468.1875	-1	1	2	2	1	-3	-1

Table S24: Found MS signals corresponding to the test set in positive mode of the *Digestion method 1* solubilized UiO-66 NH_2 substrate that was (*R*)-**1** aldehyde treated, in ascending order of m/z ratio. Monoisotopic masses and corresponding probed formulae are shown for test set building blocks. Composition is represented by string of integers.

		M_mi [Da]	181.0375	90.0317	58.0419	46.0419	18.0106
m/z [ ]	Charge [ ]	Ionization	$C_8H_7NO_4$	$C_3H_6O_3$	C_3H_6O	C_2H_6O	H_2O
254.0659	1	H^+	1	1	0	0	-1
272.0765	1	H^+	1	1	0	0	0
292.0218	1	K^+	1	1	0	0	-1
310.0324	1	K^+	1	1	0	0	0
332.0531	1	K^+	1	1	1	0	-2
338.0637	1	K^+	1	1	0	1	-1
350.0637	1	K^+	1	1	1	0	-1
361.1003	1	+	1	2	0	0	0
364.0429	1	K^+	1	2	0	0	-2
378.0950	1	K^+	1	1	1	1	-2
382.0535	1	K^+	1	2	0	0	-1
383.1211	1	+	1	2	1	0	-2
384.0901	1	Na^+	1	2	0	0	0
388.1003	1	Na^+	1	2	1	0	-3
389.1316	1	+	1	2	0	1	-1
390.1395	1	H^+	1	2	0	1	-1
400.0641	1	K^+	1	2	0	0	0
404.0742	1	K^+	1	2	1	0	-3
410.0848	1	K^+	1	2	0	1	-2
411.1524	1	+	1	2	1	1	-3
412.1602	1	H^+	1	2	1	1	-3
422.0848	1	K^+	1	2	1	0	-2
424.1214	1	Na^+	1	2	1	0	-1
428.0954	1	K^+	1	2	0	1	-1
429.1629	1	+	1	2	1	1	-2
430.1708	1	H^+	1	2	1	1	-2
434.1422	1	Na^+	1	2	1	1	-3
440.0954	1	K^+	1	2	1	0	-1
464.1527	1	Na^+	1	2	2	0	-2
468.1267	1	K^+	1	2	1	1	-2
472.0367	1	Cs^+	1	1	1	1	-2
480.1267	1	K^+	1	2	2	0	-2
516.0265	1	Cs^+	1	2	1	0	-2
544.0578	1	Cs^+	1	2	1	1	-3

Table S25: Found MS signals corresponding to the test set in negative mode of the *Digestion method 1* solubilized UiO-66 NH_2 substrate that was (*R*)-**1** aldehyde treated, in ascending order of m/z ratio. Monoisotopic masses and corresponding probed formulae are shown for test set building blocks. Composition is represented by string of integers.

	M_mi [Da]	181.0375	90.0317	58.0419	46.0419	18.0106	1.0078
m/z [ ]	Charge [ ]	$C_8H_7NO_4$	$C_3H_6O_3$	C_3H_6O	C_2H_6O	H_2O	H^+
168.5586	-2	1	1	1	1	-2	-2
181.5483	-2	1	2	1	0	-3	-2
184.5535	-2	1	2	0	1	-2	-2
190.5535	-2	1	2	1	0	-2	-2
204.5692	-2	1	2	1	1	-3	-2
252.0514	-1	1	1	0	0	-1	-1
270.0619	-1	1	1	0	0	0	-1
292.0827	-1	1	1	1	0	-2	-1
310.0932	-1	1	1	1	0	-1	-1
338.1245	-1	1	1	1	1	-2	-1
364.1038	-1	1	2	1	0	-3	-1
370.1144	-1	1	2	0	1	-2	-1
382.1144	-1	1	2	1	0	-2	-1
400.1249	-1	1	2	1	0	-1	-1
410.1457	-1	1	2	1	1	-3	-1

Table S26: Subset of the 100 most intense MS signals corresponding to the test set, in positive mode, of the *Model 2* ethanolous solution, in descending order of intensity. Monoisotopic masses and corresponding probed formulae are shown for test set building blocks. Composition is represented by string of integers.

			M_mi [Da]	181.0375	90.0317	58.0419	46.0419	18.0106
m/z [ ]	Intensity (%)	Charge [ ]	Ionization	$C_8H_7NO_4$	$C_3H_6O_3$	C_3H_6O	C_2H_6O	H_2O
294.0973	103.84	1	H^+	1	1	1	0	-2
316.0791	20.77	1	Na^+	1	1	1	0	-2
332.0531	6.81	1	K^+	1	1	1	0	-2
470.2020	5.78	1	H^+	1	2	2	1	-3
340.1391	3.68	1	H^+	1	1	1	1	-2
366.1184	3.31	1	H^+	1	2	1	0	-3

Table S27: Subset of the 100 most intense MS signals corresponding to the test set, in negative mode, of the *Model 2* ethanolous solution, in descending order of intensity. Monoisotopic masses and corresponding probed formulae are shown for test set building blocks. Composition is represented by string of integers.

		M_mi [Da]	181.0375	90.0317	58.0419	46.0419	18.0106	1.0078
m/z [ ]	Intensity (%)	Charge [ ]	$C_8H_7NO_4$	$C_3H_6O_3$	C_3H_6O	C_2H_6O	H_2O	H^+
292.0828	16.48	-1	1	1	1	0	-2	-1
338.1247	5.18	-1	1	1	1	1	-2	-1
468.1872	1.31	-1	1	2	2	1	-3	-1
382.1140	0.74	-1	1	2	1	0	-2	-1
310.0933	0.57	-1	1	1	1	0	-1	-1

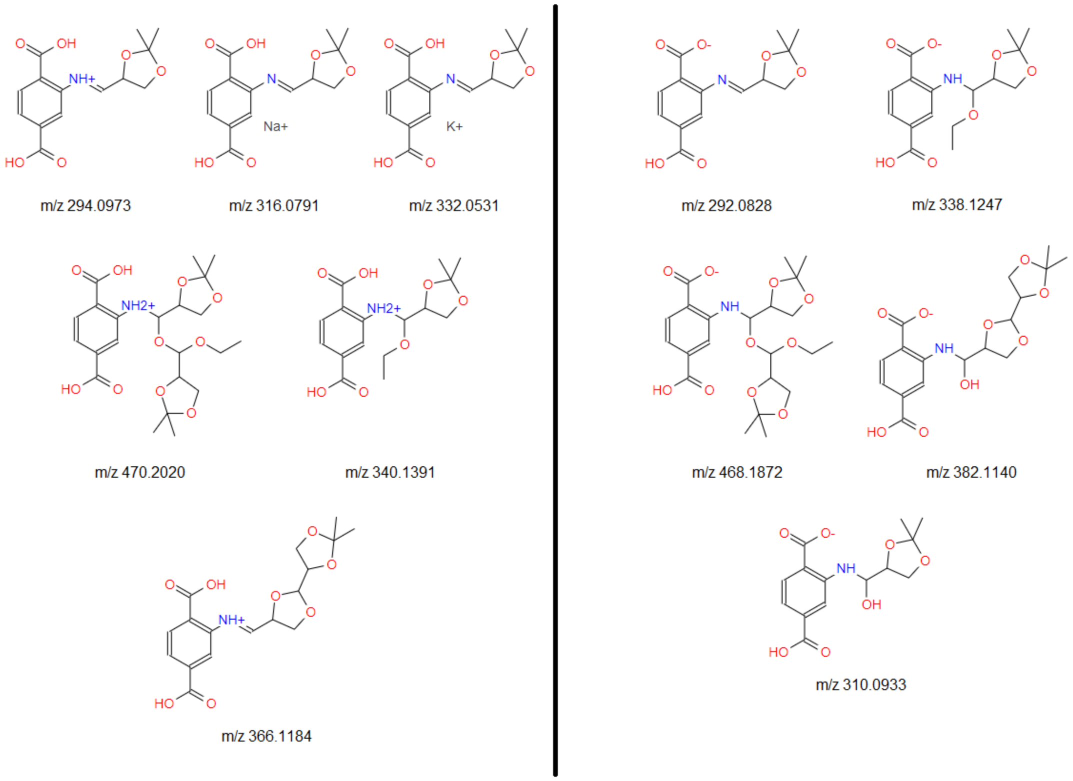

Figure S45: The structure of the ions for the subset of the 100 most intense respective MS signals corresponding to the test set, of the *Model 2* ethanolous solution. Shown separately for positive mode (left), and negative mode (right). Respective order of structures corresponds to signal intensity.

Table S28: Subset of the 100 most intense MS signals corresponding to the test set, in negative mode, of the *Digestion method 1* solubilized *Model 2* residue, in descending order of intensity. Monoisotopic masses and corresponding probed formulae are shown for test set building blocks. Composition is represented by string of integers.

		M_mi [Da]	181.0375	90.0317	58.0419	46.0419	18.0106	1.0078
m/z [ ]	Intensity (%)	Charge [ ]	$C_8H_7NO_4$	$C_3H_6O_3$	C_3H_6O	C_2H_6O	H_2O	H^+
338.1248	100.07	-1	1	1	1	1	-2	-1
292.0828	38.78	-1	1	1	1	0	-2	-1

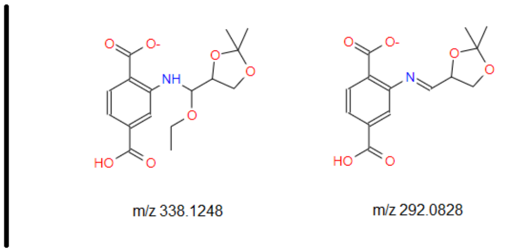

Figure S46: The structure of the ions for the subset of the 100 most intense respective MS signals corresponding to the test set, of the *Digestion method 1* solubilized *Model 2* residue. Shown separately for positive mode (left), and negative mode (right). Respective order of structures corresponds to signal intensity.

Table S29: Subset of the 100 most intense MS signals corresponding to the test set, in negative mode, of *Digestion method 1* solubilized UiO-66 NH_2 substrate that was (R)-**1** aldehyde treated, in descending order of intensity. Monoisotopic masses and corresponding probed formulae are shown for test set building blocks. Composition is represented by string of integers.

		M_mi [Da]	181.0375	90.0317	58.0419	46.0419	18.0106	1.0078
m/z [ ]	Intensity (%)	Charge [ ]	$C_8H_7NO_4$	$C_3H_6O_3$	C_3H_6O	C_2H_6O	H_2O	H^+
338.1246	11.21	-1	1	1	1	1	-2	-1
252.0514	3.79	-1	1	1	0	0	-1	-1
168.5586	3.16	-2	1	1	1	1	-2	-2
292.0828	1.71	-1	1	1	1	0	-2	-1

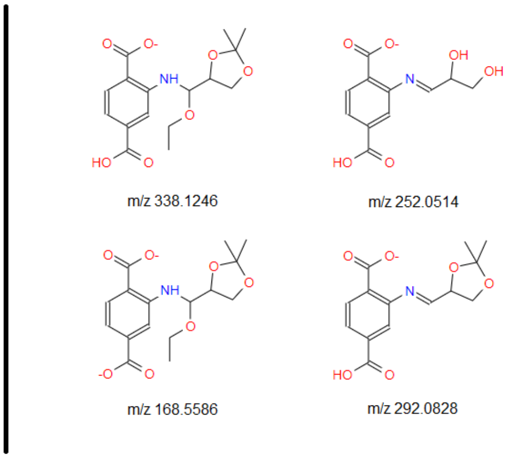

Figure S47: The structure of the ions for the subset of the 100 most intense respective MS signals corresponding to the test set, of the *Digestion method 1* solubilized UiO-66 NH_2 substrate that was (R)-**1** aldehyde treated. Shown separately for positive mode (left), and negative mode (right). Respective order of structures corresponds to signal intensity.

3.6 Circular dichroism analyses

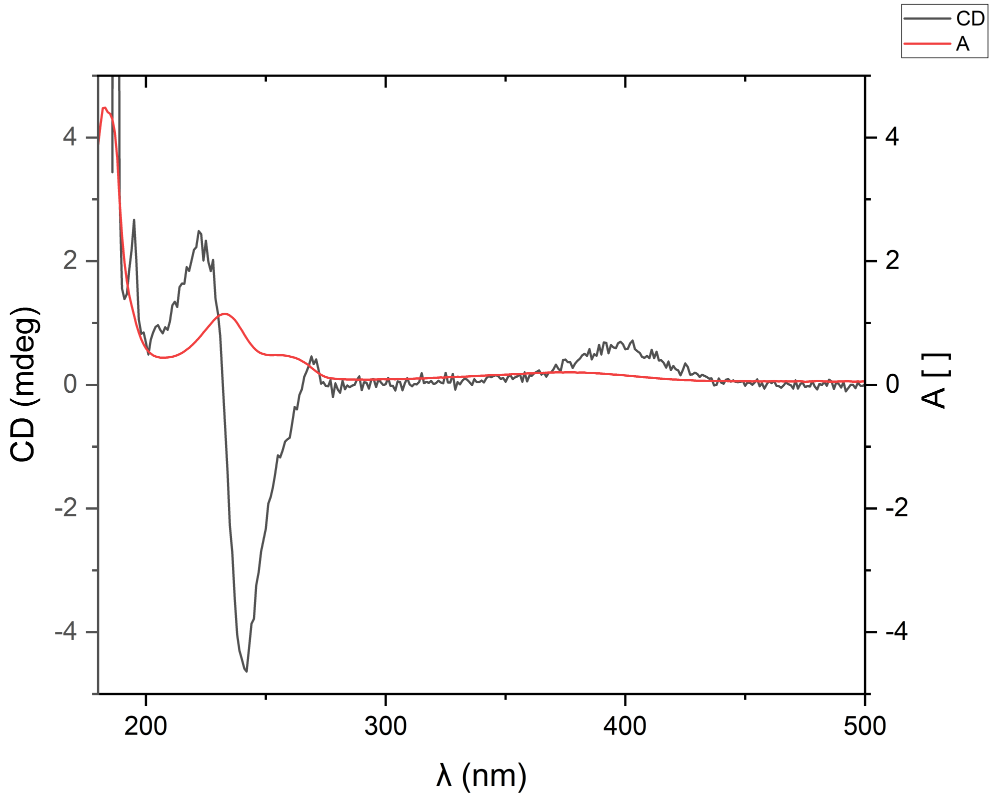

Figure S48: The overlaid CD and absorbance spectra of the methanolous solution of *Model 1* experiment, diluted 100-fold into ethanol. Retention of enantiomeric excess is demonstrated. Cotton effect is apparent.[12]

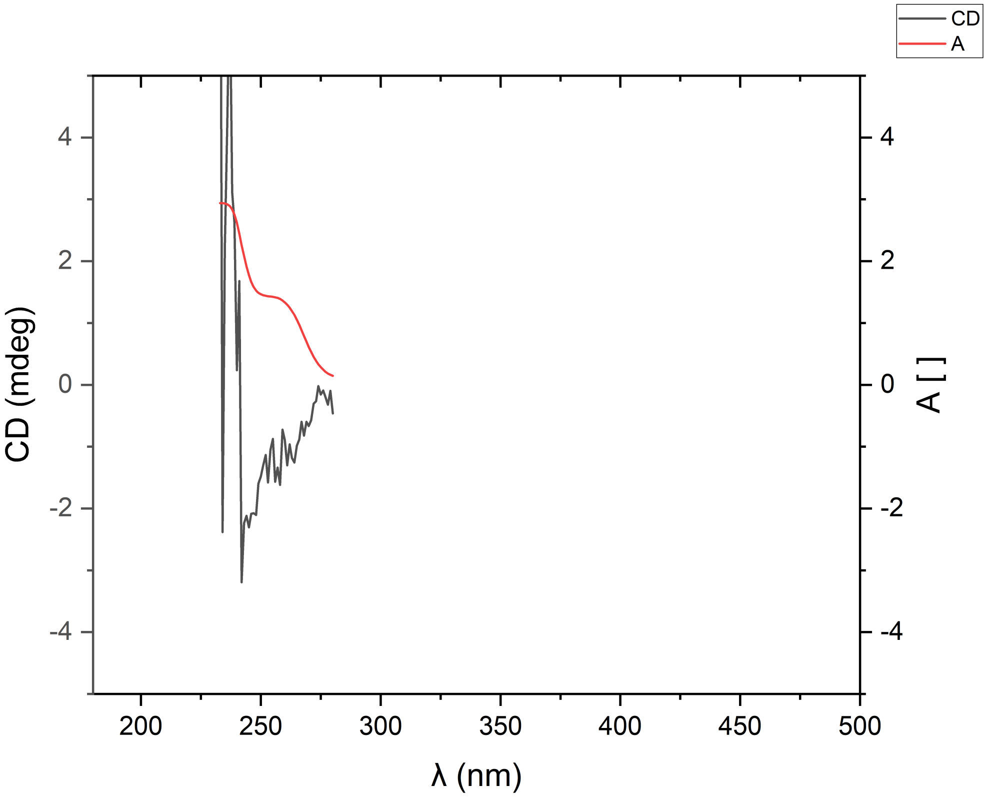

Figure S49: The overlaid CD and absorbance spectra of the ethanolous solution of *Model 3* experiment, diluted 5-fold into ethanol. Retention of enantiomeric excess is demonstrated. Cotton effect is apparent.[12]

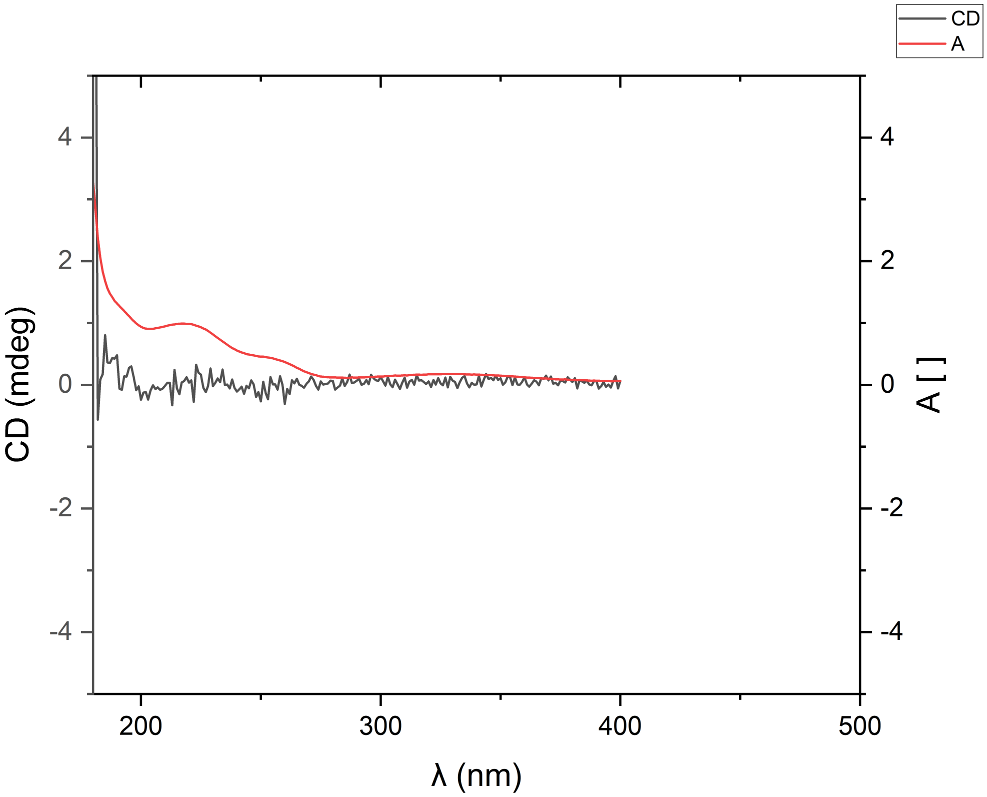

Figure S50: The overlaid CD and absorbance spectra of the *Digestion method 1* solubilized UiO-66 NH_2 substrate that was (R)-**1** aldehyde treated, diluted 70-fold into water. No clear indication of enantiomeric excess found, despite the absorbance spectrum.

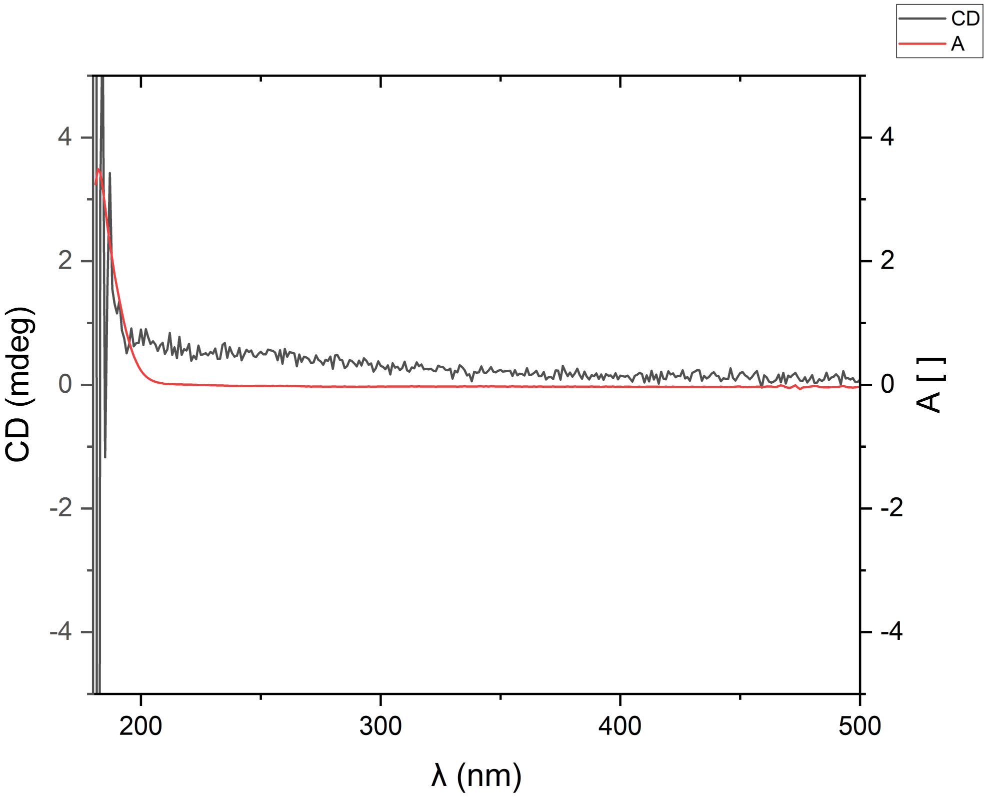

Figure S51: The overlaid CD and absorbance spectra of the *Digestion method 2* solubilized UiO-66 NH_2 substrate that was (*R*)-**1** aldehyde treated, no further dilution was applied. Solubilization was found to be insufficient based on the absorbance spectrum.

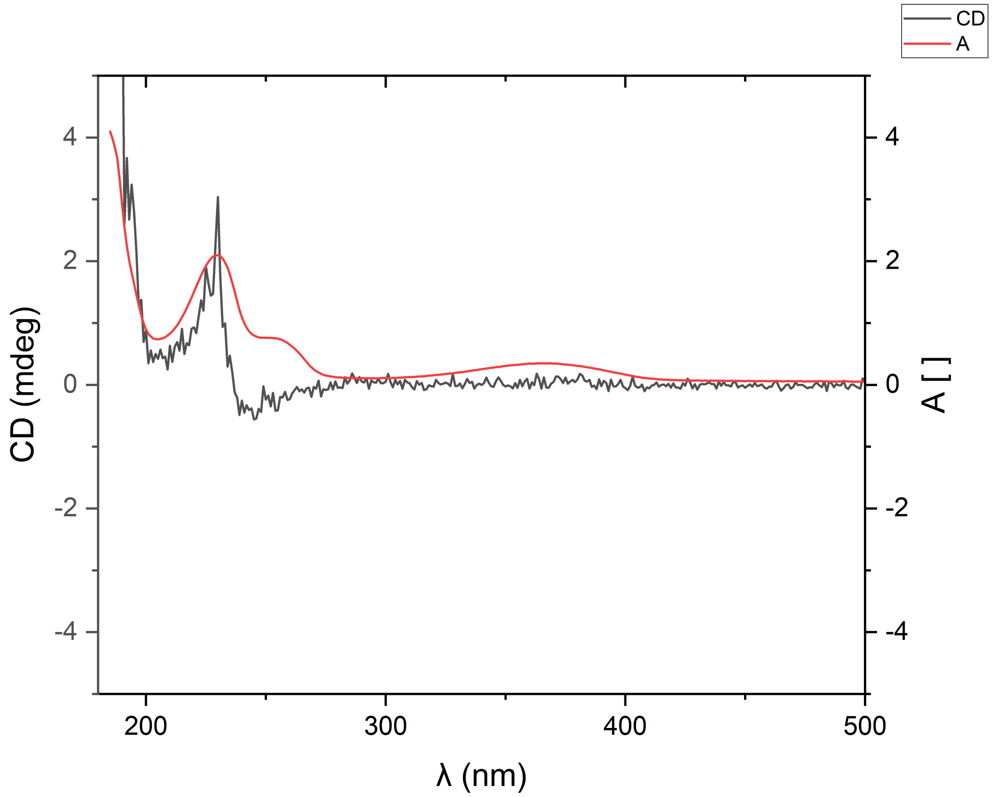

Figure S52: The overlaid CD and absorbance spectra of the ethanolous solution of *Model 2* experiment, diluted 150-fold into ethanol. No unequivocal indication of enantiomeric excess found, in light of the absorbance spectrum.

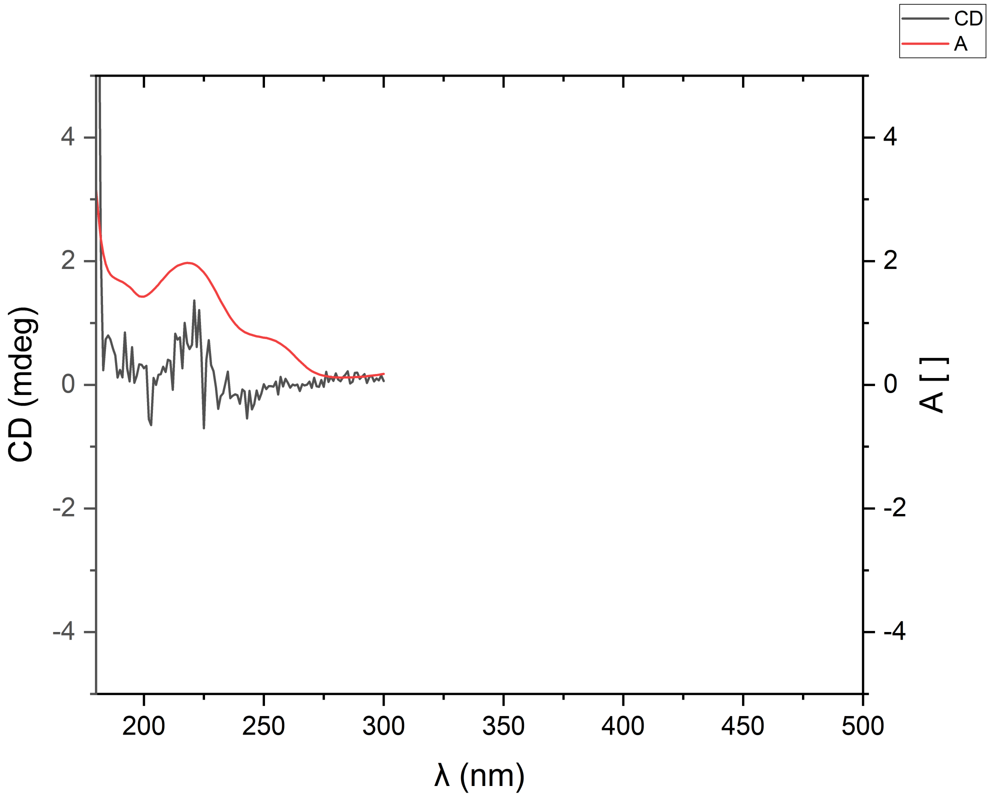

Figure S53: The overlaid CD and absorbance spectra of the *Digestion method 2* solubilized residue of *Model 2* experiment, diluted 180-fold into water. No clear indication of enantiomeric excess found, despite the absorbance spectrum.

Quantitative determination of the enantiomeric excess would require the isolation of the enantiomeric mixture of a single species. Furthermore, it would require a resolved calibration sample of one of the enantiomers of the said species. Given the chemical non-uniformity of the system, this was impeded, and thus not pursued.

4 Computational methods

4.1 Models of prospective chiralized structures

The 12-connected Zr metal node of UiO-66 NH_2 and Ti metal node of MIL-125 NH_2 along with the unmodified and modified ligands were used to build the *in silico* MOFs in the *fcu* topology, using the ToBaCCo algorithm.[13,14] The *in silico* generated structures were then optimized using the Universal Forcefield (UFF)[15] with the LAMMPS package.[16,17] The input files for the forcefield optimization were generated using LAMMPS interface.[18,19]

4.2 The DFT optimization of structures and ligands

4.2.1 Molecular systems

The modified ligands were optimized using DFT with the B3LYP exchange-correlated functional implemented in Gaussian 16.[20] The atoms were represented with a 6-31G* basis set. The calculations were performed using a fine grid for the two-electron integrals.

4.2.2 Periodic systems

The *in silico* periodic structures were further fully optimized using DFT as implemented in the CP2K software package.[21] Calculations with CP2K used the QUICKSTEP program and mixed Gaussian and plane wave basis sets in combination with Goedecker-Teter-Hutter (GTH) pseudopotentials.[22] The efficient orbital transformation (OT) method[23] was used for the optimization of the wave function. The double-ζ polarization MOLOPT basis sets were used to describe organic element atoms, while a triple-ζ was used for Zr atoms.

4.3 Pore analyses

The pore diameters of the DFT optimized structures (before and after ligand modification) were computed using the Zeo++ software package,[24] and are summarized below.

Table S30: Pore diameters (in Å) for the (a) unmodified UiO-66 NH_2 and the (b) uniformly (R)-**1** aldehyde modified UiO-66 NH_2 MOF structures. Di stands for the largest cavity diameter, Df is the pore limiting diameter, and Dif is the largest cavity diameter along the free sphere path

MOF	Di	Df	Dif
(a)	7.288	3.654	7.215
(b)	3.646	1.387	3.642

References

(1) Jablonka, K. M.; Moosavi, S. M.; Asgari, M.; Ireland, C.; Patiny, L.; Smit, B. A data-driven perspective on the colours of metal–organic frameworks. *Chemical science* **2021**, *12*, 3587–3598.

(2) Patiny, L.; Zasso, M.; Kostro, D.; Bernal, A.; Castillo, A. M.; Bolaños, A.; Asencio, M. A.; Pellet, N.; Todd, M.; Schloerer, N.; others The C6H6 NMR repository: an integral solution to control the flow of your data from the magnet to the public. *Magnetic Resonance in Chemistry* **2018**, *56*, 520–528.

(3) Hotelling, H. The Generalization of Student's Ratio. *Ann. Math. Statist.* **1931**, *2*, 360–378.

(4) Johnson, R.; Wichern, D. *Applied Multivariate Statistical Analysis*; Applied Multivariate Statistical Analysis; Pearson Prentice Hall, 2007.

(5) Bradski, G. The OpenCV Library. *Dr. Dobb's Journal of Software Tools* **2000**,

(6) Origin(Pro), Version 2021b (Academic). 2021.

(7) Pascale, D. RGB coordinates of the Macbeth ColorChecker. *The BabelColor Company* **2006**, *6*, 6.

(8) Bruce Lindbloom. `http://www.brucelindbloom.com/index.html?Eqn_RGB_XYZ_Matrix.html`, accessed December 19, 2024.

(9) Demir, H. V.; Nizamoglu, S.; Erdem, T.; Mutlugun, E.; Gaponik, N.; Eychmüller, A. Quantum dot integrated LEDs using photonic and excitonic color conversion. *Nano Today* **2011**, *6*, 632–647.

(10) Kampouri, S.; Ebrahim, F. M.; Fumanal, M.; Nord, M.; Schouwink, P. A.; Elzein, R.; Addou, R.; Herman, G. S.; Smit, B.; Ireland, C. P.; others Enhanced visible-light-driven

hydrogen production through MOF/MOF heterojunctions. *ACS Applied Materials & Interfaces* **2021**, *13*, 14239–14247.

(11) Jablonka, K. M.; Patiny, L.; Smit, B. Making molecules vibrate: Interactive web environment for the teaching of infrared spectroscopy. *Journal of Chemical Education* **2022**, *99*, 561–569.

(12) Cotton, A. Recherches sur l'absorption et la dispersion de la lumière par les milieux doués du pouvoir rotatoire. *Journal de Physique Théorique et Appliquée* **1896**, *5*, 237–244.

(13) Colón, Y. J.; Gómez-Gualdrón, D. A.; Snurr, R. Q. Topologically Guided, Automated Construction of Metal-Organic Frameworks and Their Evaluation for Energy-Related Applications. *Cryst. Growth Des.* **2017**, *17*, 5801–5810.

(14) tobacco_3.0. `https://github.com/tobacco-mofs/tobacco_3.0`, using April 8, 2021 release throughout this study.

(15) Rappé, A. K.; Casewit, C. J.; Colwell, K.; Goddard III, W. A.; Skiff, W. M. UFF, a Full Periodic Table Force Field for Molecular Mechanics and Molecular Dynamics Simulations. *J. Am. Chem. Soc.* **1992**, *114*, 10024–10035.

(16) Thompson, A. P.; Aktulga, H. M.; Berger, R.; Bolintineanu, D. S.; Brown, W. M.; Crozier, P. S.; in't Veld, P. J.; Kohlmeyer, A.; Moore, S. G.; Nguyen, T. D.; others LAMMPS-a flexible simulation tool for particle-based materials modeling at the atomic, meso, and continuum scales. *Comput. Phys. Commun.* **2022**, *271*, 108171.

(17) LAMMPS. `https://github.com/lammps/lammps`, using January 7, 2022 release throughout this study.

(18) Boyd, P. G.; Mohamad Moosavi, S.; Witman, M.; Smit, B. Force-Field Prediction

of Materials Properties in Metal-Organic Frameworks. *J. Phys. Chem. Lett.* **2017**, *8*, 357–363.

(19) LAMMPS Interface. `https://github.com/peteboyd/lammps_interface`, using May 8, 2021 release throughout this study.

(20) Frisch, M. J. et al. Gaussian˜16 Revision C.01. 2016; Gaussian Inc. Wallingford CT.

(21) Kühne, T. D. et al. CP2K: An electronic structure and molecular dynamics software package - Quickstep: Efficient and accurate electronic structure calculations. *J. Chem. Phys.* **2020**, *152*, 194103.

(22) Goedecker, S.; Teter, M.; Hutter, J. Separable dual-space Gaussian pseudopotentials. *Phys. Rev. B* **1996**, *54*, 1703–1710.

(23) VandeVondele, J.; Hutter, J. An efficient orbital transformation method for electronic structure calculations. *J. Chem. Phys.* **2003**, *118*, 4365–4369.

(24) Willems, T. F.; Rycroft, C. H.; Kazi, M.; Meza, J. C.; Haranczyk, M. Algorithms and Tools for High-Throughput Geometry-Based Analysis of Crystalline Porous Materials. *Microporous Mesoporous Mater.* **2012**, *149*, 134–141.
